# Beclin1 restricts RNA virus infection in plants through suppression and degradation of the viral polymerase

**DOI:** 10.1038/s41467-018-03658-2

**Published:** 2018-03-28

**Authors:** Fangfang Li, Changwei Zhang, Yinzi Li, Guanwei Wu, Xilin Hou, Xueping Zhou, Aiming Wang

**Affiliations:** 1 London Research and Development Centre, Agriculture and Agri-Food Canada, London, ON N5V 4T3 Canada; 2 Department of Biology, Western University, London, ON N6A 5B7 Canada; 3 State Key Laboratory of Crop Genetics and Germplasm Enhancement, Nanjing Agricultural University, 210095 Nanjing, China; 4 State Key Laboratory for Biology of Plant Diseases and Insect Pests, Institute of Plant Protection, Chinese Academy of Agricultural Sciences, 100193 Beijing, China

## Abstract

Autophagy emerges as an essential immunity defense against intracellular pathogens. Here we report that turnip mosaic virus (TuMV) infection activates autophagy in plants and that Beclin1 (ATG6), a core component of autophagy, inhibits virus replication. Beclin1 interacts with NIb, the RNA-dependent RNA polymerase (RdRp) of TuMV, via the highly conserved GDD motif and the interaction complex is targeted for autophagic degradation likely through the adaptor protein ATG8a. Beclin1-mediated NIb degradation is inhibited by autophagy inhibitors. Deficiency of Beclin1 or ATG8a enhances NIb accumulation and promotes viral infection and vice versa. These data suggest that Beclin1 may be a selective autophagy receptor. Overexpression of a Beclin1 truncation mutant that binds to NIb but lacks the ability to mediate NIb degradation also inhibits virus replication. The Beclin1–RdRp interaction further extends to several RNA viruses. Thus Beclin1 restricts viral infection through suppression and also likely autophagic degradation of the viral RdRp.

## Abstract (web summary)

Plant DNA virus replication is inhibited by autophagy, but the interplay between plant RNA viruses and autophagy is less clear. Here, Li et al. show that turnip mosaic virus infection activates autophagy and that Beclin1, a core autophagy component, binds the viral polymerase and inhibits virus replication.

## Introduction

Macroautophagy (hereafter referred to as autophagy) is an evolutionarily conserved intracellular process in which double membrane-bound sack-like vesicles, termed autophagosomes, are biosynthesized to enclose and deliver cytoplasmic materials including macromolecular complexes and organelles to the lysosome/vacuole for degradation/recycling^1–5^. Autophagy may be divided into several major steps, including induction, nucleation and expansion of the phagophore, maturation of the autophagosomes, and docking/fusion with the vacuole or lysosome, followed by degradation and breakdown^6–8^. Extensive genetic screens of yeast mutants have led to the identification of ~40 autophagy-related genes (*ATG*s), and approximately half of them are indispensable for the formation of autophagosomes^3, 5, 8^. Most of these key *ATG*s in yeast have orthologs in mammals and plants^1,2,5,6,9–11^. During canonical autophagy, the corresponding gene products of the key *ATG*s form several functionally distinct groups to assemble the core autophagy machinery^5–8^. One of them is the phosphatidylinositol 3-kinase (PI3K) complex containing PI3K/VPS34, VPS15/p150, and Beclin1/ATG6/VPS30.

Initially, autophagy was assumed to be a rather unspecific, adaptive bulk catabolic process to provide energy and recycle nutrients for survival upon starvation stress^12^. It is now well established that autophagy can operate either in a non-selective or a highly selective mode, depending on the type of cargo to be degraded. Selective autophagy can target a variety of substrates ranging from individual proteins and protein complexes to entire organelles and invading microbes^13,14^. It involves the recruitment of specific receptor and adaptor proteins that simultaneously interact with specific cargoes and autophagy modifiers.

Autophagy is activated during infection by diverse viruses in metazoans^15,16^. Although autophagy has been suggested to play both antiviral and proviral roles, compelling evidence suggests that autophagy participates in innate and adaptive immune responses to eliminate pathogenic viruses^17^. For instance, in *Drosophila*, vesicular stomatitis virus infection induces autophagy, and depletion of *ATGs* by RNAi leads to increased virus replication and fly mortality^18^. In mice, ATG5 plays an essential role against lethal infection of the mouse central nervous system by *Sindbis virus*^19^. In plants, Liu and colleagues pioneered the research by showing that the autophagy pathway is required for *N-*gene mediated resistance to *Tobacco mosaic virus* (TMV)^20^. In contrast to the wild-type plants showing typical hypersensitive response (HR) upon TMV infection, plants deficient in the autophagy genes, *Beclin1*, *PI3K*/*VPS34*, *ATG3*, and *ATG7*, exhibit an unrestricted HR^20^. The suggestion of autophagy as an important component of plant immunity has been consolidated by two recent studies on the role of autophagy against two DNA viruses *Cotton leaf curl Multan virus* (CLCuMuV) and *Cauliflower mosaic virus* (CaMV)^21–23^. Hafréd and colleagues showed that autophagosomes are induced in CaMV-infected Arabidopsis, and the autophagy cargo receptor Neighbor of BRCA1 (NBR1) interacts with capsid proteins and mediates their degradation^21^. Consistently, Haxim el al. found that CLCuMuV infection induces autophagy in *Nicotiana benthamiana*^22^. In this case, the cargo acceptor is NbATG8f, an ATG8 family protein, which interacts with the satellite-encoded protein βC1, a major pathogenicity factor of CLCuMuV, and the interaction directs βC1 to autophagosomes for degradation^22^.

The majority of viruses infecting animals and plants are positive-sense RNA viruses. The molecular interactions between RNA viruses and plant autophagy still remain poorly understood. Potyviruses represent the largest group of known plant viruses with a positive-sense, single-stranded RNA as its genome and include many agriculturally important viruses such as *Turnip mosaic virus* (TuMV), *Tobacco etch virus* (TEV), *Plum pox virus* (PPV), and *Soybean mosaic virus* (SMV)^24^. A typical potyviral genome encodes a long open reading frame (ORF) and another relatively short ORF that results from RNA polymerase slippage in the P3 coding sequence. The resulting two polyproteins are proteolytically processed by three viral protease domains into 11 mature viral proteins, among which NIb is the only viral RNA-dependent RNA polymerase (RdRp), and HC-Pro and VPg are two known viral suppressors of RNA silencing (VSRs).

In this study, we show that TuMV infection activates autophagy and that Beclin1, one of the core ATGs that are upregulated by TuMV infection, interacts specifically with TuMV NIb, and targets NIb to autophagosomes for degradation likely via the key autophagic protein ATG8a. The interaction of NIb with Beclin1 also suppresses NIb RdRp activity in an autophagy-independent manner. Moreover, Beclin1 interacts with the RdRps of several other potyviruses as well as distinct RNA viruses via the GDD motif. These results suggest that Beclin1 restricts viral infection through suppression and also likely autophagic degradation of the viral RdRp.

## Results

### TuMV infection activates autophagy

**Fig. 1:**
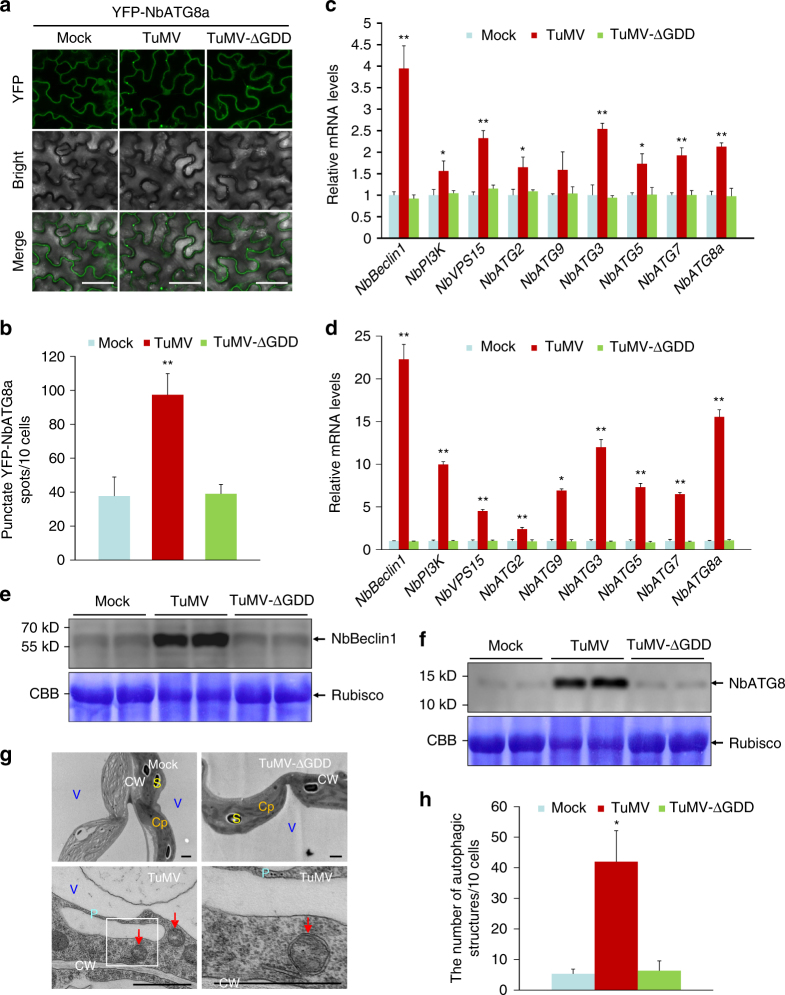
TuMV infection activates autophagy. **a** Confocal micrographs showing *N*. *benthamiana* leaf cells co-infiltrated with *Agrobacterium* harboring a YFP-NbATG8a expression construct and *Agrobacterium* carrying an empty vector (mock) or a TuMV infectious clone (TuMV) or a TuMV replication-defective mutant (TuMV-ΔGDD) at 60 h post infiltration (hpi). Bars, 50 μm. **b** The average number of YFP-NbATG8a spots per 10 cells. Infiltration experiments were repeated three times and 60 cells in total were counted for the punctate spots. The average number was calculated using 10 cells as a unit. Values represent the mean spots ±standard deviation (SD) per 10 cells. **c**, **d** Effects of viral infection on the expression of autophagy components at 3 and 7 days post infiltration (dpi). *N*. *benthamiana* leaves were agroinfiltrated with mock, TuMV, or TuMV-ΔGDD. Total RNAs were extracted from infiltrated zones at 3 dpi (**c**) or from newly emerged leaves at 7 dpi (**d**). Values represent the mean relative to the mock-treated plants (*n* = 3 biological replicates) and were normalized with *NbActin* as an internal reference. **e**, **f** Immunoblotting analysis of total protein isolated from upper non-inoculated leaves of plants agroinfiltrated with mock, TuMV, and TuMV-ΔGDD at 7 dpi with anti-Beclin1 (**e**) or anti-ATG8 (**f**) antibody. Coomassie Brilliant Blue R-250 (CBB)-stained Rubisco large subunit serves as a loading control. **g** Representative TEM images from upper non-inoculated leaves of *N*. *benthamiana* plants infected with mock, TuMV, and TuMV-ΔGDD at 7 dpi. Obvious autophagic structures (red arrows) were observed in TuMV-infected samples and the corresponding region in the white box in the left panel is magnified in the right panel. Cp chloroplast, CW cell wall, P particle, S starch, V vacuole. Bars = 1 μm. **h** The number of typical double-membrane autophagosomes in different samples in **g**. Experiments were repeated twice and typical autophagic structures were counted in 30 cells in each treatment. Values represent the mean number of autophagosomes ±SD per 10 cells. **b**, **c**, **d**, **h** Data were analyzed using Student’s *t*-test and asterisks denote significant differences between mock (or TuMV-ΔGDD) and TuMV-infected leaves (two-sided, *P < 0.05, **P < 0.01)

The autophagy pathway genes are constitutively expressed at the basal level to maintain homeostasis and are upregulated in response to abiotic and biotic stress^13,14^. To determine whether TuMV infection induces autophagy, we used yellow fluorescent protein (YFP)-tagged *N*. *benthamiana* ATG8a (YFP-NbATG8a) as an autophagosome marker to monitor autophagy^25,26^. Compared to mock-infiltrated *N*. *benthamiana* plants, the number of the punctate YFP fluorescent structures representing pre-autophagosomal or autophagosomal structures in TuMV-infected plants increased by more than two-folds (Fig. 1a, b and Supplementary Fig. 1a,b). A TuMV replication-defective mutant (TuMV-ΔGDD), in which the highly conserved GDD motif of NIb was deleted, was included in this assay. This deletion abolishes viral replication but still allows the production of viral proteins, expression of which is controlled by a 35S promoter. No significant changes were detected between the numbers of the punctate YFP-ATG8a fluorescent structures in mock- and TuMV-ΔGDD-inoculated plants (Fig. 1a, b and Supplementary Fig. 1a,b). These data indicate that TuMV infection activates autophagy.

We further examined the expression of *ATG* genes in these plants using quantitative real-time reverse-transcription PCR (qRT-PCR). The expression levels of all tested nine *ATG* genes, including *NbBeclin1*, *NbPI3K*, *NbVPS15*, *NbATG2*, *NbATG9*, *NbATG3*, *NbATG5*, *NbATG7*, and *NbATG8a*, were significantly upregulated in TuMV-inoculated leaves (Fig. 1c) or upper non-inoculated leaves (Fig. 1d), compared to their respective counterparts in mock- or TuMV-ΔGDD-inoculated plants. Transient expression of each of the 11 potyviral proteins alone under the control of 35S promoter failed to significantly upregulate these 9 *ATG* genes (Supplementary Fig. 2a, NIb used an example). As NIb is the only viral RdRp, we further confirmed that there were no significant differences in NbBeclin1 and NbATG8a induction and the amount of YFP-NbATG8a puncta at different NIb levels (Supplementary Fig. 2b–f). Consistently, high levels of Beclin1 and ATG8 proteins were detected only in TuMV-infected plants by immunoblotting (Fig. 1e, f). Transmission electron microscopy (TEM) was employed to further monitor autophagic activity induced by TuMV infection. In comparison with mock or TuMV-ΔGDD systemically infected leaves, TuMV infection induced an approximate eight-fold greater number of double-membrane structures typical of autophagosomes in the cytoplasm^27^ (Fig. 1g, h). Taken together, these results demonstrate that TuMV infection induces autophagy likely in a viral replication-dependent manner.

### NbBeclin1 interacts with the viral RdRp

**Fig. 2:**
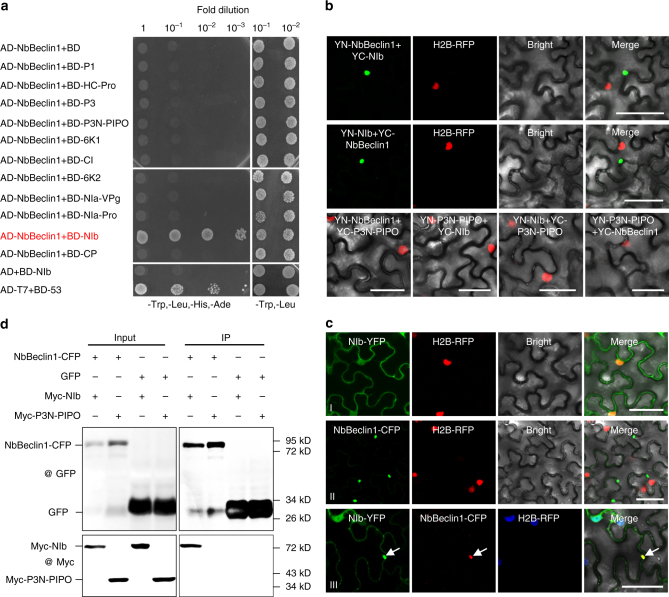
NbBeclin1 interacts with NIb. **a** Yeast-two hybrid (Y2H) assays for possible interactions between NbBeclin1 and each of the 11 TuMV proteins. NbBeclin1 and 11 viral proteins were fused with a GAL4 activation domain (AD-NbBeclin1) and a GAL4-binding domain (BD-P1, BD-HC-Pro, BD-P3, BD-P3N-PIPO, BD-6K1, BD-CI, BD-6K2, BD-NIa-VPg, BD-NIa-Pro, BD-NIb, BD-CP), respectively. Y2H Gold yeast cells co-transformed with the indicated plasmids were subjected to 10-fold serial dilutions and plated on synthetic dextrose (SD)/-Trp, -Leu, -His, -Ade or SD/-Trp, -Leu medium to screen for positive interactions at 3 days after transformation. Yeast co-transformed with AD-T7-T+BD-T7-53 serves as a positive control; yeast cells co-transformed with AD-NbBeclin1 and the empty BD or with the empty AD and BD-NIb are negative controls. **b** BiFC assays between NbBeclin1 and NIb in the leaves of H2B-RFP transgenic *N*. *benthamiana*. Confocal imaging was performed at 48 hpi. NbBeclin1 and NIb were fused to the N (YN) and C-terminal (YC) fragments of yellow fluorescent protein (YFP). The NbBeclin1-NIb interaction led to the reconstituted fluorescence-competent structure and restoration of yellow fluorescence (green). Nuclei of tobacco leaf epidermal cells are indicated by the expression of H2B-RFP transgene (red). Bars, 50 μm. **c** Co-localization of NIb-YFP with NbBeclin1-CFP in the leaf cells of H2B-RFP transgenic *N*. *benthamiana* by confocal microscopy at 48 hpi. Arrow indicates yellow fluorescence, which was produced from the overlapping of NIb-YFP (green) and NbBeclin1-CFP (red). Bars, 50 μm. **d** Co-immunoprecipitation (Co-IP) analysis of NbBeclin1-CFP and Myc-NIb in vivo. *N*. *benthamiana* leaves were co-infiltrated with *A*. *tumefaciens* cells harboring expression vectors to express NbBeclin1-CFP and Myc-NIb (Lane 1), NbBeclin1-CFP and Myc-P3N-PIPO (Lane 2), Myc-NIb and GFP (Lane 3), and GFP and Myc-P3N-PIPO (Lane 4). Leaf protein extracts were incubated with GFP-Trap®_MA magnetic agarose beads (ChromoTek). Samples before (Input) and after (IP) immunopurification were analyzed by immunoblotting using GFP or Myc antibody

As *NbBeclin1* (*ATG6* in yeast or Arabidopsis) was the most upregulated gene (Fig. 1c, d) and its mammalian orthologous gene *Beclin1* has been implicated in viral infection in mammalian cells^28,29^, possible interactions between NbBeclin1 and each of the 11 TuMV proteins^30^ were screened for by yeast two-hybrid (Y2H) assays. Positive protein–protein interaction was found between NbBeclin1 and TuMV NIb, and no interaction was evident between NbBeclin1 with any other TuMV proteins (Fig. 2a). The NbBeclin1–NIb interaction was confirmed by bimolecular fluorescence complementation (BiFC) in transgenic *N*. *benthamiana* leaves that express H2B-RFP, a nuclear marker (Fig. 2b). NIb-YFP was present in the cytoplasm and nucleus^31–33^. NbBeclin1-CFP was localized to a single or a few bright dots in the cytoplasm (Fig. 2c and Supplementary Fig. 3a), consistent with the distribution pattern of Arabidopsis AtATG6/VPS30^34^ (Supplementary Fig. 3a,b). When NIb-YFP and NbBeclin1-CFP were co-expressed, some NIb-YFP was re-distributed to the NbBeclin1-CFP-labeled punctate structures in the cytoplasm (Fig. 2c). Co-immunoprecipitation (Co-IP) assay showed that green fluorescent protein (GFP) antibody could specifically co-purify NbBeclin1-CFP and Myc-NIb (Fig. 2d), confirming the presence of the NbBeclin1-NIb complex in planta.

**Fig. 3:**
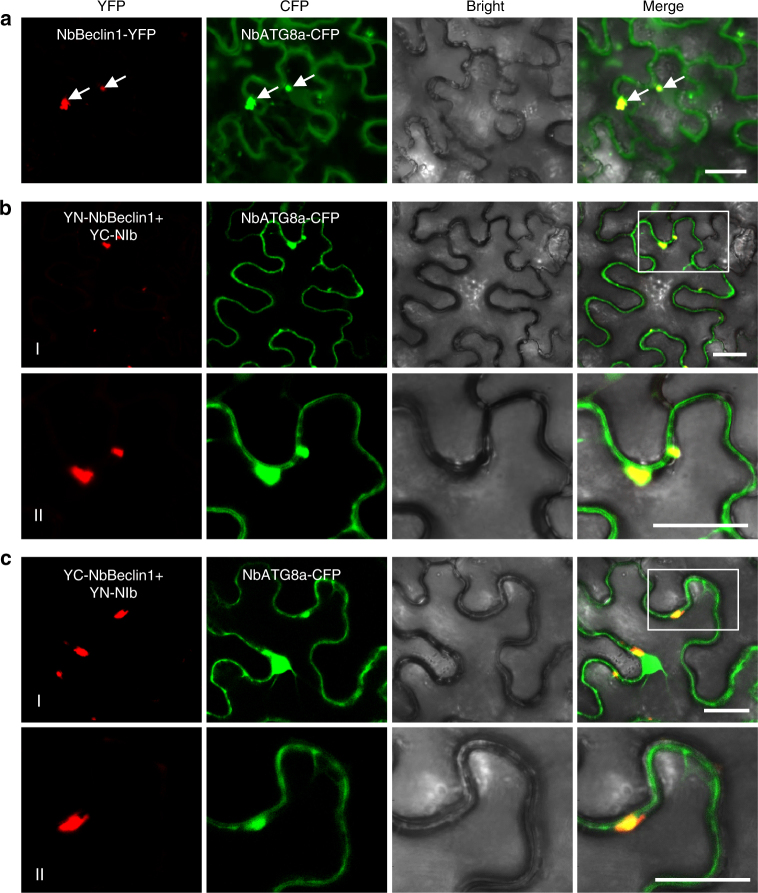
NbBeclin1 or the NbBeclin1–NIb interaction complex co-localizes with the autophagosome marker NbATG8a in *N*. *benthamiana*. **a** Transient co-expression of NbBeclin1-YFP and NbATG8a-CFP. Arrows indicate yellow fluorescence, which resulted from the overlapping of NbBeclin1-YFP (red) and NbATG8a-CFP (green) fluorescence. **b** Co-localization of YN-NbBeclin1 and YC-NIb with NbATG8a-CFP. **c** Co-localization of YC-NbBeclin1 and YN-NIb with NbATG8a-CFP. All confocal micrographs in this figure were taken at 48 hpi. The corresponding region in the white box in the panel I of **b**, **c** is magnified in panel II. Bars, 25 µm

AtATG6/VPS30-labeled punctate structures are co-distributed with ATG8, an established autophagosome marker^34^. We thus tested the possible association of the NIb–NbBeclin1 complex with ATG8a. Co-expression and BiFC analyses showed that NbBeclin1-YFP and the NIb-NbBeclin1 complex indeed co-localized with NbATG8a-CFP to the punctate structure within the cytoplasm (Fig. 3a–c). These results indicate that the NbBeclin1-NIb complex is physically associated with autophagosomes.

**Fig. 4:**
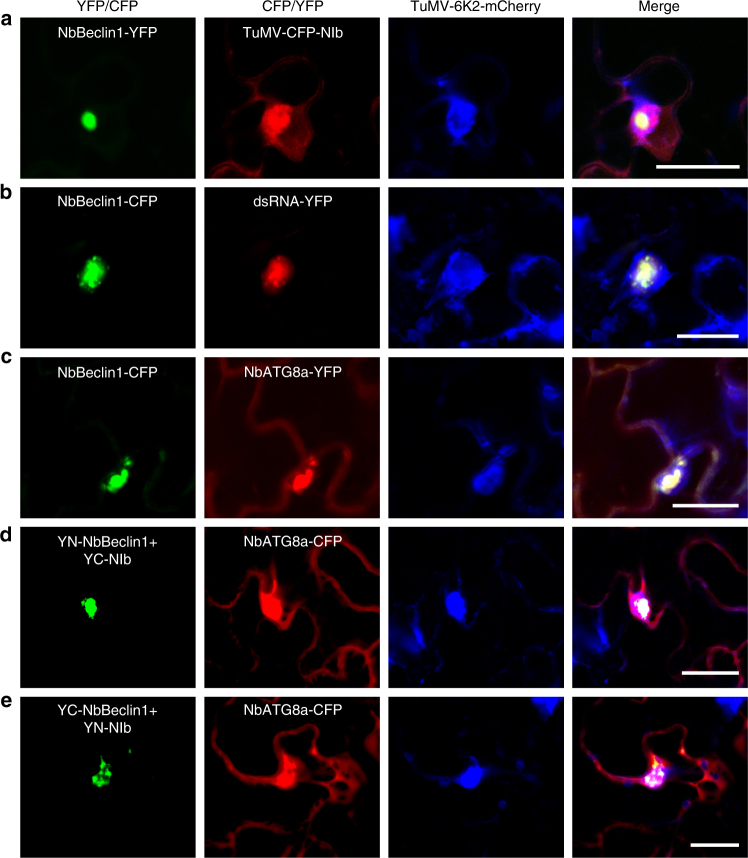
NbBeclin1 or the NbBeclin1–NIb complex co-localizes with the TuMV viral replication complex (VRC) in autophagosomes in *N*. *benthamiana*. **a** NbBeclin1-YFP was transiently expressed in leaf cells infected by TuMV-6K2-mCherry-CFP-NIb. NbBeclin1-YFP co-localized with CFP-NIb and 6K2-mCherry-stained aggregations. **b** Transient expression of NbBeclin1-CFP and a dsRNA marker (B2-YN+VP35-YC) in *N*. *benthamiana* leaves infected by TuMV-6K2-mCherry. NbBeclin1 co-localized with dsRNA in TuMV VRCs. **c** Co-localization of NbBeclin1 with NbATG8a and the TuMV VRC. **d**, **e** Co-localization of the interaction complex of NbBeclin1 and NIb with NbATG8a and the TuMV VRC. All confocal micrographs shown in this figure were taken at 72 hpi. Bars, 25 µm

Since NIb is an essential component of the viral replication complex (VRC), we hypothesized that NbBeclin1 is associated with the VRC through its interaction with NIb. We thus conducted a co-localization assay using NbBeclin1-YFP and a modified TuMV infectious clone TuMV-CFP-NIb^35^ (by insertion of cyan fluorescent protein (CFP) to the N-terminus of NIb in the infectious clone TuMV-6K2-mCherry^36^). The 6K2-induced structure derived from viral expression is an established marker of the VRC^35^. NbBeclin1-YFP co-localized with the 6K2-induced, CFP-NIb-containing irregular structures in the cytoplasm in TuMV-infected leaf cells (Fig. 4a). A dsRNA reporter^35^ that recognizes dsRNA such as replicative RNA intermediates also bound to the NbBeclin1-interacting, 6K2-induced structures, further supporting that they were active VRCs (Fig. 4b). To test whether the NbBeclin1-bound VRC interacts with autophagosomes, NbATG8a-YFP or NbATG8a-CFP was transiently co-expressed with NbBeclin1-CFP or the pair of YN-NbBeclin1/YC-NIb or YC-NbBeclin1/YN-NIb in *N*. *benthamiana* infected by TuMV-6K2-mCherry. NbATG8a-YFP or NbATG8a-CFP was indeed associated with the 6K2-induced VRC, which co-localized with NbBeclin1 or the NbBeclin1–NIb interaction complex (Fig. 4c–e). It was worthy to note that there was no co-localization between 6K2 and NbATG8a or NbBeclin1 when they were co-expressed together in the absence of viral replication in *N*. *benthamiana* leaves (Supplementary Fig. 4a–c). These results indicate that NbBeclin1 co-localizes with the VRC by binding to NIb.

### NbBeclin1 mediates NIb degradation likely via autophagy

**Fig. 5:**
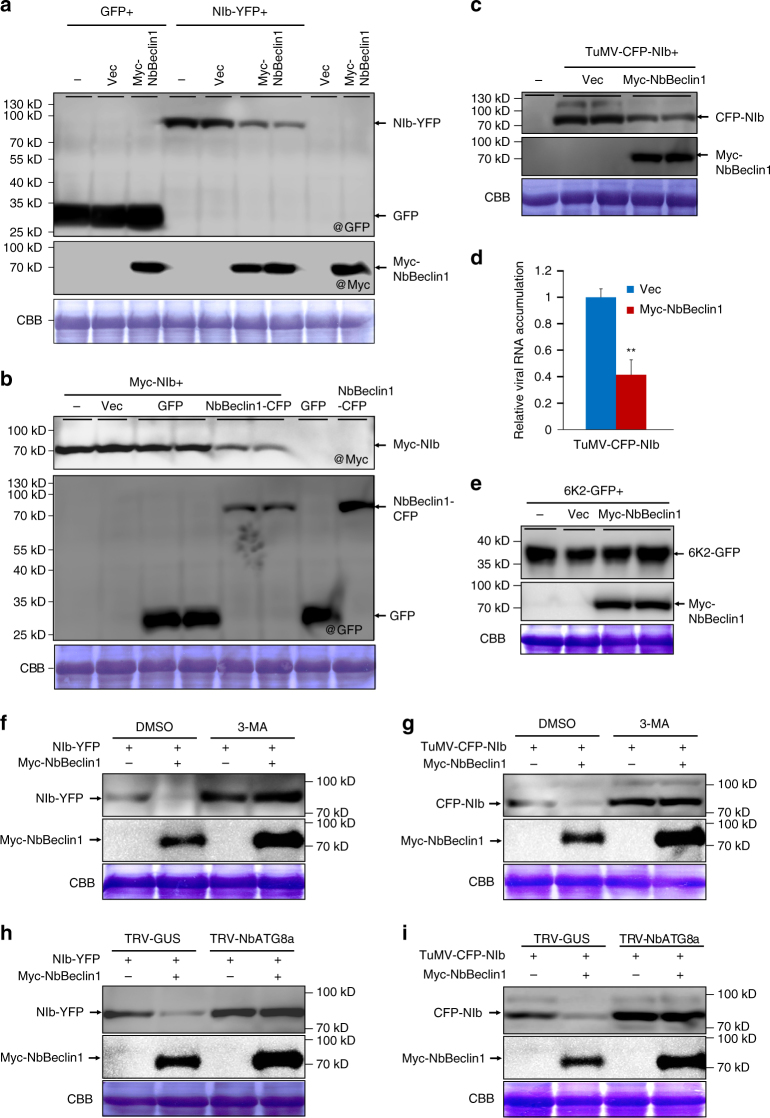
Overexpression of NbBeclin1 promotes autophagy-dependent degradation of NIb and inhibits TuMV replication. **a**, **b** Immunoblotting of total protein extracts from the *N*. *benthamiana* leaves agroinfiltrated with buffer (−) or the plasmids indicated. The membrane was probed with GFP (@GFP), or Myc antibodies (@Myc). **c** Immunoblotting analysis of total protein extracts from leaves infiltrated with buffer (−) or TuMV-CFP-NIb together with an empty vector (Vec) or Myc-NbBeclin1 with antibodies against GFP or Myc. **d** Quantification of TuMV RNA levels by qRT-PCR. RNA was extracted from leaves agroinfiltrated with TuMV-CFP-NIb together with Vec or Myc-NbBeclin1 at 60 hpi. Values represent means ± SD relative to plants infiltrated with TuMV-CFP-NIb and Vec (*n* = 3 biological replicates). The data were analyzed using Student’s *t*-test and asterisks denote significant differences between the two treatments (two-sided, **P < 0.01). **e** Immunoblotting analysis of total protein extracts from leaves co-infiltrated with 6K2-GFP and buffer (−), empty vector (Vec), or Myc-NbBeclin1. Antibodies against GFP or Myc were used as a primary antibody. **f**, **g** The effect of the autophagy inhibitor 3-MA on the NbBeclin1-mediated degradation of NIb-YFP or TuMV-CFP-NIb. Total proteins were isolated from plant leaves agroinfiltrated with NIb-YFP alone or with Myc-NbBeclin1 (**f**) or TuMV-CFP-NIb alone or with Myc-NbBeclin1 (**g**) followed by DMSO or 3-MA treatment. **h**, **i** The effect of silencing of *NbATG8a* on the NbBeclin1-mediated degradation of NIb-YFP or CFP-NIb. Plants inoculated with TRV-GUS or TRV-NbATG8a at 14 dpi were agroinfiltrated with NIb-YFP alone or with Myc-NbBeclin1 (**h**) or TuMV-CFP-NIb alone or with Myc-NbBeclin1 (**i**). Total protein was extracted from infiltrated leaves at 3 dpi. Immunoblotting was performed using GFP or Myc antibodies. All immunoblotting assays in this figure were repeated at least three times, and one representative blot was shown. CBB staining of Rubisco large subunit serves as a loading control

As NbBeclin1 may serve as a receptor protein for the autophagic clearance of viral proteins, we investigated whether NbBeclin1 has a role in the degradation of NIb. Immunoblotting analysis revealed that the levels of NIb-YFP markedly decreased when co-expressed with Myc-NbBeclin1 (Fig. 5a). A similar reduction was also observed when Myc-NIb was co-expressed with NbBeclin1-CFP (Fig. 5b). No obvious change in *NIb* or *NbBeclin1* mRNA was observed when they were expressed alone or together (Supplementary Fig. 5a,b). To determine whether NbBeclin1-mediated NIb degradation holds true in the context of viral infection, we co-infiltrated *N*. *benthamiana* leaves with the infectious clone TuMV-CFP-NIb and an empty vector (Vec) or TuMV-CFP-NIb and Myc-NbBeclin1. Immunoblotting analysis confirmed that the levels of CFP-NIb from viral expression markedly decreased when co-expressed with Myc-NbBeclin1 (Fig. 5c). In contrast, overexpression of NbBeclin1 did not affect 6K2-GFP accumulation from non-viral expression (Fig. 5e). It is well known that virus intercellular movement does not occur for potyviruses until 72 hpi^36^. We thus determined the viral RNA level in the primarily infected cells at 60 hpi by qRT-PCR. We found that overexpression of NbBeclin1 significantly inhibited viral RNA accumulation (Fig. 5d). These results suggest that NbBeclin1-mediated degradation of NIb and its complexes including VRCs inhibits viral replication.

Drug treatment with 3-methyladenine (3-MA) and E64d, two well-known autophagy inhibitors, was performed to test whether the autophagy pathway is responsible for NbBeclin1-mediated degradation of NIb. 3-MA treatment enhanced the accumulations of NIb-YFP or CFP-NIb derived from viral expression (Supplementary Fig. 5c,d). In 3-MA-treated plants, the TuMV RNA level also increased (Supplementary Fig. 5e). E64d treatment showed similar results (Supplementary Fig. 5f,g). To observe the effect of impaired autophagy flux on the accumulation of autophagic bodies and viral genomic RNA, we conducted another drug treatment with the specific vacuolar ATPase inhibitor concanamycin A (Con A). Con A treatment led to the accumulation of significantly more autophagic bodies associated with VRCs and of enhanced levels of TuMV RNA at 72 hpi, compared to DMSO treatment, supporting the antiviral role of autophagy in viral infection (Supplementary Fig. 6).

Next, we determined whether NbBeclin1-mediated degradation of NIb was affected by autophagy inhibitors or by knockdown of *NbATG8*. Regardless of co-expression with NbBeclin1 or not, the levels of NIb-YFP or viral expression-derived CFP-NIb increased in 3-MA- or E64d-treated plants (Fig. 5f, g and Supplementary Fig. 5c,d,f,g). A TRV-based virus-induced gene silencing (VIGS) vector was employed to silence *NbATG8a* and *NbATG8f*, and knockdown of *NbATG8a* rather than *NbATG8f* remarkably inhibited NbBeclin1-mediated degradation of NIb-YFP or CFP-NIb (Fig. 5h, i and Supplementary Fig. 7a–c). A GFP protein that is relatively resistant to the autophagy pathway was used as a control (Fig. 5a, b and Supplementary Fig. 5h–k).

### Mapping of the interacting domains

**Fig. 6:**
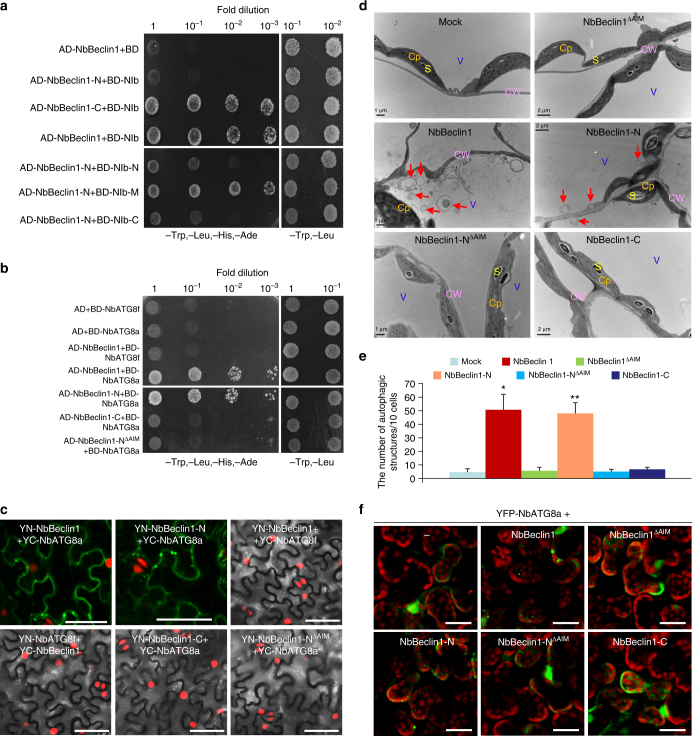
The GDD motif of NIb is required for the NbBeclin1–NIb interaction and NbBeclin1, via its AIM, interacts with NbATG8a to facilitate the formation of autophagosomes. **a** Y2H assays to detect possible interactions between NbBeclin1 truncated proteins (NbBeclin1-N and NbBeclin1-C) and NIb and between NbBeclin1 and NIb truncated proteins (NIb-N, NIb-M, and NIb-C). **b** Y2H assays to detect possible interactions between NbBeclin1 and NbATG8f or NbATG8a and between NbATG8a and NbBeclin1-N, NbBeclin1-C, or NbBeclin1-N AIM mutant (NbBeclin1- N^ΔAIM^). **c** BiFC assays in H2B-RFP (red) transgenic *N*. *benthamiana* leaves at 48 hpi. Yellow fluorescence (green) was observed as a consequence of the complementation of the YN and YC tagged with NbBeclin1 and NbATG8a or NbBeclin1-N and NbATG8a. Bars, 50 μm. **d** Representative TEM images from *N*. *benthamiana* leaf cells agroinfiltrated with buffer (mock), NbBeclin1, NbBeclin1^ΔAIM^, NbBeclin1-N, NbBeclin1-N^ΔAIM^, or NbBeclin1-C at 60 hpi. Typical autophagic structures (red arrows) were observed in NbBeclin1- or NbBeclin1-N-expressing leaves in the cytoplasm. Cp chloroplast, CW cell wall, S starch, V vacuole. Bars mean 1 μm or 2 μm as indicated. **e** The number of typical double-membrane autophagic structures in mock, NbBeclin1-, NbBeclin1^ΔAIM^-, NbBeclin1-N-, NbBeclin1-N^ΔAIM^-, or NbBeclin1-C-infiltrated leaves. Experiments were repeated three times and typical autophagic structures were counted in 20 cells in each treatment. Values represent the mean number of autophagosomes ±SD per 10 cells. Single asterisk indicates statistically significant difference (*P* < 0.05) between mock and NbBeclin1-infiltrated leaves, and double asterisks indicate *P* < 0.01 between mock and NbBeclin1-N (Student’s *t*-test, two-sided). **f** Confocal micrographs showing *N*. *benthamiana* leaf cells co-infiltrated with *Agrobacterium* harboring a YFP-NbATG8a expression construct and *Agrobacterium* carrying NbBeclin1, NbBeclin1^ΔAIM^, NbBeclin1-N, NbBeclin1-N^ΔAIM^ or NbBeclin1-C at 48 hpi. Bars, 25 μm

The protein domains required for the interaction between NbBeclin1 and NIb were mapped by Y2H assays (Fig. 6a and Supplementary Fig. 8a–d). The C-terminal portion (containing the APG6 domain) of NbBeclin1 (NbBeclin1-C) and the RdRp domain of NIb (NIb-M) were identified to be responsible for the NbBeclin1 and NIb interaction (Fig. 6a and Supplementary Fig. 8a–d). This observation was consistent with results from BiFC assays in planta (Supplementary Fig. 8e). The NIb-interacting domain NbBeclin1-C was also essential for the punctate localization of NbBeclin1 (Supplementary Fig. 8f).

Since the conserved GDD motif is located in NIb-M, we tested the importance of this motif with respect to the NIb–NbBeclin1 interaction. Deletion of GDD abolished the interaction (Supplementary Fig. 8b,d,g). As NIb-M is highly conserved among potyviruses^32^, we investigated the potential interaction between NbBeclin1 and NIbs from other potyviruses, including PPV, SMV, and TEV. Positive interactions were detected by Y2H assays (Supplementary Fig. 8g); consistently, GDD deletion extirpated the interaction (Supplementary Fig. 8g).

For selective autophagy, receptor proteins require binding to adaptors to facilitate docking of autophagy substrates to the autophagosomes^37^. To test whether NbBeclin1 binds to the autophagy adaptors ATG8 family proteins, the possible interaction between NbBeclin1 and NbATG8a or NbATG8f was analyzed. Y2H, BiFC, and Co-IP experiments consistently showed that NbBeclin1 interacted strongly with NbATG8a but not NbATG8f (Fig. 6b, c and Supplementary Fig. 9). The NbATG8-interacting domain was mapped to the N-terminal region of NbBeclin1 (NbBeclin1-N) (Fig. 6b, c and Supplementary Fig. 9). A web-based analysis (http://repeat.biol.ucy.ac.cy/iLIR)^38^ identified a potential ATG8 interacting motif (AIM) in NbBeclin1-N. The NbBeclin1-N AIM mutant (NbBeclin1-N^ΔAIM^) resulting from the replacement of EESFVVL with EESAVVA lost the ability to interact with NbATG8a (Fig. 6b, c and Supplementary Fig. 9). NbBeclin1^ΔAIM^ was still able to form bright granules, interact with, and co-localize with NIb in the cytoplasm (Supplementary Fig. 10a–c), consistent with the observation that the C-domain of NbBeclin1 (NbBeclin1-C) was required for NIb interaction and its punctate localization (Fig. 6a and Supplementary Fig. 8e,f). Autophagic structures were analyzed by TEM in *N*. *benthamiana* leaves transiently expressing NbBeclin1, NbBeclin1^ΔAIM^, NbBeclin1-N, NbBeclin1-N^ΔAIM^, or NbBeclin1-C. Compared to buffer treatment (mock) and the expression of NbBeclin1^ΔAIM^, NbBeclin1-N^ΔAIM^, or NbBeclin1-C, the expression of NbBeclin1 or NbBeclin1-N induced the formation of the typical double-membraned autophagosomes (Fig. 6d–f). Further, an autophagic flux assay on NbBeclin1 and its truncated mutants was conducted. Treatment of YFP:NbATG8a-expressing *N*. *benthamiana* leaves with Con A increased the number of NbATG8a-labeled puncta in the presence of NbBeclin1 or NbBeclin1-N but not other NbBeclin1 mutants (including NbBeclin1^ΔAIM^, NbBeclin1-N^ΔAIM^, or NbBeclin1-C) (Supplementary Fig. 11a,b), indicating that NbBeclin1-N and its AIM was required to induce and enhance autophagy flux. These observations were also confirmed using western blot analyses (Supplementary Fig. 11c). The expression of NbBeclin1 or NbBeclin1-N, rather than NbBeclin1^ΔAIM^, NbBeclin1-N^ΔAIM^, or NbBeclin1-C, decreased the levels of YFP-NbATG8a protein (Supplementary Fig. 11c). Treatment of these samples with E64d, an inhibitor of vacuolar cysteine proteases, blocked NbBeclin1- or NbBeclin1-N-mediated YFP-NbATG8a degradation (Supplementary Fig. 11c). These data suggest that NbBeclin1 induces autophagy through interacting with the autophagy adaptor NbATG8a via AIM (located at the N-terminus) and serves as an autophagy receptor by interacting with cargo proteins via its C-terminus.

### NbBeclin1 also suppresses NIb independent of autophagy

**Fig. 7:**
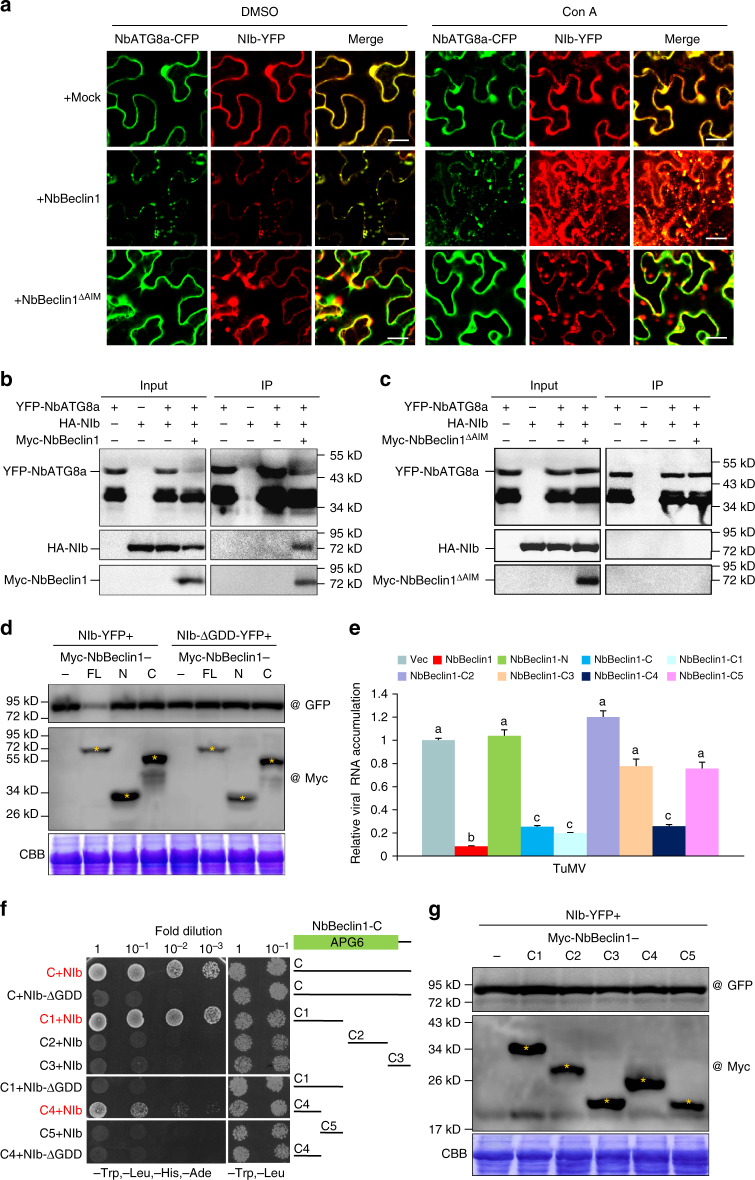
NbBeclin1 via its AIM interacting with NbATG8a mediates NIb degradation to repress viral replication, and one C-terminal fragment of NbBeclin1 also inhibits viral replication by binding to NIb independent of autophagy degradation of NIb. **a** Co-localization of NbATG8a-CFP with NIb-YFP in the expression of empty vector (+mock), Myc-tagged NbBeclin1 (+NbBeclin1) or Myc-tagged NbBeclin1 AIM mutant (+NbBeclin1^ΔAIM^) in *N*. *benthamiana* leaf cells. Infiltrated leaves were treated with DMSO or concanamycin A (Con A) after 48 hpi and confocal images were taken at 10 h after treatment. Bars, 25 μm. **b**, **c** Co-IP analysis of the association of NIb with NbATG8a in the presence of Myc-NbBeclin1 or Myc-NbBeclin1^ΔAIM^ in planta. *N*. *benthamiana* leaves were agroinfiltrated with the plasmids indicated. Leaf protein extracts were incubated with GFP-Trap®_MA magnetic agarose beads (ChromoTek). Samples before (Input) and after (IP) immunopurification were analyzed by immunoblotting using GFP, HA, or Myc antibody. **d** Total protein extracts from the *N*. *benthamiana* leaves expressing the indicated recombinant plasmids were subjected to immunoblotting analysis using GFP (@GFP) or Myc antibody (@Myc). Minus sign (−) means that NIb-YFP or NIb-ΔGDD-YFP was expressed alone. All immunoblotting assays in this figure were repeated at least three times, and one representative blot was shown. CBB staining of Rubisco large subunit serves as a loading control. Yellow asterisks indicate the expected sizes. **e** Quantification of TuMV RNA levels by qRT-PCR. RNA was extracted from leaves infiltrated with TuMV together with Vec, NbBeclin1, NbBeclin1-N, NbBeclin1-C, NbBeclin1-C1, NbBeclin1-C2, NbBeclin1-C3, NbBeclin1-C4, or NbBeclin1-C5 at 60 hpi. Values represent means ±SD (*n* = 3 biological replicates) and are presented as arbitrary units relative to Vec. According to Duncan’s multiple range test (*P* = 0.01), the means do not differ significantly if they are indicated with the same letter. **f** The C1 domain and C4 domain in the C-terminal region of NbBeclin1 interact with NIb in the Y2H assay. A series of truncated NbBeclin1 proteins from NbBeclin1-C are indicated. **g** The truncated proteins of NbBeclin1-C fail to degrade NIb. Immunoblotting analysis of the total protein extracts from the *N*. *benthamiana* leaves expressing the plasmids indicated. Minus sign (−) means that NIb-YFP was expressed alone. Yellow asterisks indicate the expected sizes

NbBeclin1 co-localized and interacted with NbATG8a and NIb to form puncta and induce autophagy flux in the cytoplasm (Fig. 7a, b), and mediated their degradation (Fig. 7b and Supplementary Fig. 11d). The AIM deletion mutant NbBeclin1^ΔAIM^ lost this capacity (Fig. 7a, b and Supplementary Fig. 11d). Western blots showed that NbBeclin1-mediated degradation of NbATG8a and NIb was obviously suppressed by the treatment of E64d or Con A (Supplementary Fig. 11d). These data support that NbBeclin1, as an autophagy cargo receptor to form autophagosomes and mediate cargo protein degradation, requires the AIM. Full-length NbBeclin1 (NbBeclin1-FL) and its truncated proteins (NbBeclin1-N and NbBeclin1-C) were compared for their ability to mediate NIb degradation. Both NbBeclin1-N and NbBeclin1-C lost this ability (Fig. 7d and Supplementary Fig. 11d), suggesting that NbBeclin1-N (which binds to NbATG8a via AIM to trigger autophagy) and NbBeclin1-C (which interacts with NIb) are both required for NbBeclin1-mediated NIb degradation. Consistent with the requirement of GDD for the NIb-NbBeclin1 interaction, NbBeclin1-FL failed to degrade NIb-ΔGDD-YFP (Fig. 7d). The effect of NbBeclin1, NbBeclin1-N and NbBeclin1-C on TuMV replication was analyzed. It was surprising that NbBeclin1-C failed to degrade NIb protein, but it was still able to suppress viral RNA replication (Fig. 7e). The expression of NbBeclin1 and NbBeclin1-C decreased viral RNA replication to approximately 8% and 25%, respectively (Fig. 7e). The fact that NbBeclin1-C is responsible for the NbBeclin1 and NIb interaction (Fig. 6a and Supplementary Fig. 8a–e) prompted us to consider the possibility that the interaction between NbBeclin1 and NIb may also interrupt viral replication independent of autophagy-mediated NIb degradation. Serial deletions of NbBeclin1-C were created and tested for the interaction with NIb by Y2H assays. As shown in Fig. 7f, the NIb-binding motif was mapped to the N-terminal region (C1) of NbBeclin1-C (strong interaction) or the N-terminal half (C4) of C1 (weak interaction). As expected, both C1 and C4 were unable to mediate NIb degradation (Fig. 7g). Overexpression of C1 or C4 rather than other NbBeclin1-C fragments inhibited viral replication (Fig. 7e), suggesting that NbBeclin1 binding to the RdRp domain of NIb suppresses RdRp activity in an autophagy-independent manner.

### Beclin1 and ATG8a negatively regulate TuMV infection

**Fig. 8:**
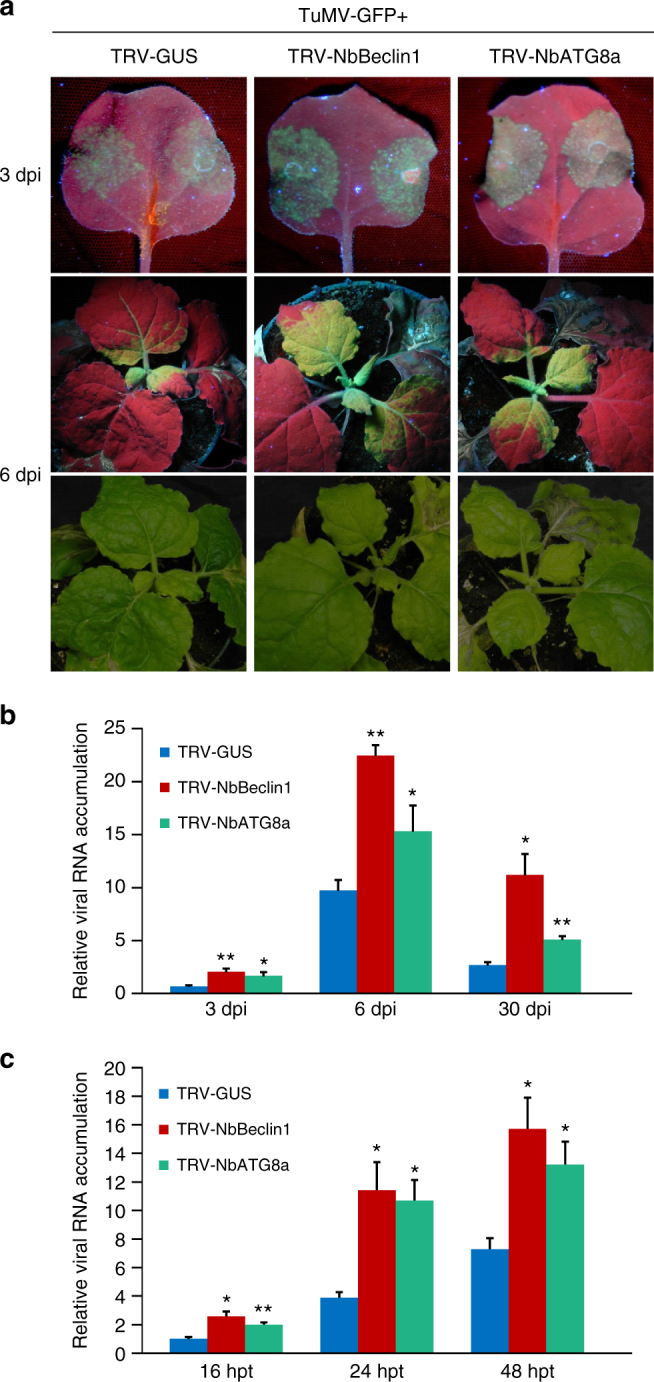
Silencing of *NbBeclin1* or *NbATG8a* promotes TuMV infection in *N*. *benthamiana*. **a** GFP fluorescence and viral symptoms in plants pre-inoculated with TRV1 together with TRV2-GUS (control), TRV2-NbBeclin1, or TRV2-NbATG8a for 7 days and then infected by TuMV-GFP. Plants were photographed under UV light at 3 and 6 dpi and under regular light at 6 dpi. **b** Quantification of TuMV genomic RNA in the above plants. RNA was extracted from TuMV-GFP-inoculated leaves at 3 dpi or systemically infected leaves at 6 dpi and 30 dpi. The values are presented as means of fold change ±SD relative to the control plants (pretreated with TVR1 and TRV2-GUS). Error bars represent SD. Three independent experiments, each consisting of three biological replicates, were carried out. Values from one representative result were used to plot a histogram and were normalized against *NbActin* transcripts in the same sample. The data were analyzed using Student’s *t*-test (two-sided, *P < 0.05, **P < 0.01). **c** Protoplast transfection assay. Protoplasts isolated from *N*. *benthamiana* plants pre-inoculated with TRV1 together with TRV2-GUS, TRV2-NbBeclin1, or TRV2-NbATG8a were transfected with TuMV-GFP. RNA was extracted at 16, 24, and 48 hpt and quantified by qRT-PCR to analyze TuMV genomic RNA accumulations. Values are presented as means ± SD, and error bars represent SD (*n* = 3 biological replicates). The data were analyzed using Student’s *t*-test and asterisks denote significant differences compared to the TuMV-infected protoplasts from control plants pre-treated with TRV1 and TRV-GUS (two-sided, *P < 0.05, **P < 0.01)

Given that NbBeclin1-mediated NIb degradation requires NbATG8a, and NbBeclin1 inhibits RdRp activity by binding to the GDD motif, we next examined the effect of silencing *NbBeclin1* or *NbATG8a* on viral replication. *N*. *benthamiana* plants were pre-inoculated with TRV*-*NbBeclin1 (to silence *NbBeclin1*), TRV-NbATG8a (to silence *NbATG8a*), or TRV-GUS (as a control) for 7 days and then agroinfiltrated with TuMV-GFP. GFP fluorescence in the inoculated leaves (at 3 dpi) and GFP progression in the upper non-inoculated leaves (at 6 and 30 dpi) were clearly enhanced in the *NbBeclin1*- or *NbATG8a*-silenced plants, compared to controls (Fig. 8a and Supplementary Fig. 12). In concordance, higher levels of TuMV genomic RNA were found in the *NbBeclin1*- or *NbATG8a*-silenced plants compared to controls (Fig. 8b). Protoplast transfection assays obtained similar results (Fig. 8c). In contrast, silencing of *NbATG8f* did not significantly affect TuMV infection (Supplementary Fig. 13). Overall, these results suggest that NbATG8a, and not NbATG8f, is required for NbBeclin1-mediated anti-TuMV defense.

To test whether this antiviral mechanism operates in another plant species, *AtATG6*, the ortholog of *NbBeclin1* in Arabidopsis, was cloned. Y2H and BiFC analyses showed strong interactions between NIb and AtATG6 (Supplementary Fig. 14a,b). We obtained an *atg6* heterozygous mutant (SALK_109281) from the Arabidopsis Biological Resource Center (ABRC) and generated AtATG6 overexpression transgenic Arabidopsis plants (35S:Myc-AtATG6) (Supplementary Fig. 15) for a TuMV infection assay. Compared to TuMV-infected wild-type Col-0, which showed typical symptoms as described^39,40^, the symptoms in 35S:AtATG6 were delayed for 3–4 days and were attenuated with mild chlorosis and slight stunting (Supplementary Fig. 14c). In contrast, *atg6* plants showed more severe symptoms, including leaf necrosis and severe stunting (Supplementary Fig. 14c). TuMV RNA levels were significantly reduced in the 35S:AtATG6 plants but elevated in the *atg6* mutants (Supplementary Fig. 14d). Similar results were obtained from the protoplast transfection assay (Supplementary Fig. 14e). We also obtained an Arabidopsis *atg8a* knockout mutant from ABRC and generated *AtATG8a* overexpression lines. Consistently, we found that knockout of *AtATG8a* enhanced viral RNA replication and symptom development, while overexpression of *AtATG8a* supressed viral infection (Supplementary Fig. 16).

### NIb degradation mediated by Beclin1 requires ATG2

To further examine whether other key autophagy pathway genes participate in suppression of viral infection, we obtained Arabidopsis knockdown mutants of genes *PI3K* and *VPS15* and Arabidopsis knockout mutants of genes *ATG2*, *ATG5*, and *ATG7* and also knocked down these genes in *N*. *benthamiana* plants using the TRV vector. When *PI3K* or *VPS15* was knocked down, the Arabidopsis or *N*. *benthamiana* plants became more susceptible (Supplementary Fig. 17), consistent with the results from *Beclin1*-deficient plants (Supplementary Fig. 14c,d). Knockout of *AtATG2* in Arabidopsis enhanced TuMV RNA accumulation (Supplementary Fig. 18). In contrast, knockout of *AtATG5* and *AtATG7* did not affect TuMV infection (Supplementary Fig. 18). Similar results were observed when these genes were silenced in *N*. *benthamiana* (Supplementary Fig. 13). Western blot analyses revealed that Beclin1-mediated degradation of NIb was compromised in *ATG2*-deficient plants but not in *ATG5*- or *ATG7*-deficient plants (Supplementary Fig. 7). These data suggest that Beclin1-mediated NIb degradation requires ATG2 but independent of ATG5 and ATG7. Overexpression of *NbBeclin1* in these *ATG*-deficient *N*. *benthamiana* plants (including *NbATG8a*-, *NbATG8f*-, *NbATG2*-, *NbATG5*-, or *NbATG7*-silenced plants) inhibited TuMV replication (Supplementary Fig. 7d), supporting that NbBeclin1 inhibits viral infection not only through NIb degradation (likely via ATG2/NbATGa-dependent autophagy) but also through degradation-independent suppression mechanisms.

### NbBeclin1 targets other viral RdRps to restrict RNA viruses

**Fig. 9:**
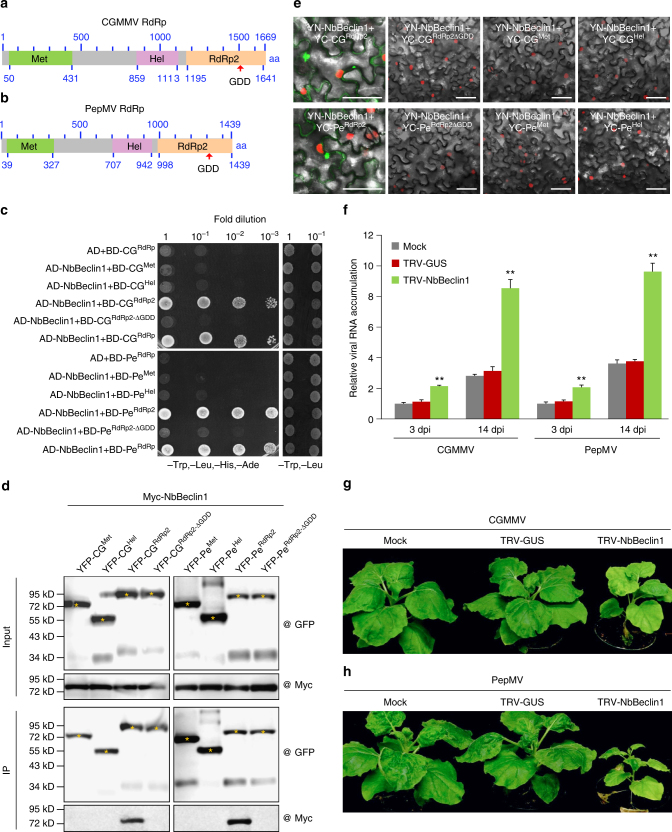
NbBeclin1 targets RdRps of plant viruses distinct to potyviruses and inhibits viral infection. **a**, **b** Schematic representation of the full-length CGMMV RdRp (**a**) and PepMV RdRp (**b**). The positions of the first and last amino acid residues of each conserved domain are indicated. Met methyltransferase domain, Hel helicase domain, RdRp2 RdRp2 domain. **c** NbBeclin1 interacts with CGMMV (CG) and PepMV (PE) RdRps or their RdRp2 domains but not with their GDD mutants or other domains, i.e., Met and Hel in the Y2H assays. **d** Co-IP analysis of possible interactions of NbBeclin1 with different domains or the GDD mutant of CGMMV and PepMV RdRps in planta. *N*. *benthamiana* leaves were agroinfiltrated with the plasmids indicated. Leaf extracts were incubated with GFP-Trap®_MA magnetic agarose beads (ChromoTek). Samples before (Input) and after (IP) immunopurification were analyzed by immunoblotting using GFP or Myc antibody. Yellow asterisks indicate the expected band sizes. **e** BiFC assays for the interaction of NbBeclin1 with different domains of CGMMV RdRp or PepMV RdRp in H2B-RFP transgenic *N*. *benthamiana* leaves at 48 hpi. Bars = 50 μm. **f** Quantification of CGMMV or PepMV RNA levels by qRT-PCR. The plants were pre-inoculated with buffer (mock), TRV1+TRV2-GUS, or TRV1+TRV2-NbBeclin1 for 7 days. RNA was extracted from CGMMV or PepMV–inoculated or systemically infected leaves at 3 dpi and 14 dpi, respectively. The values are presented as means of fold change ±SD relative to mock-treated plants. Error bars represent SD. Three independent experiments, each consisting of three biological replicates, were carried out. Values from one representative result were used to plot a histogram and were normalized with *NbActin* as the internal reference. The data were analyzed using Student’s *t*-test and double asterisks denote significant differences compared to the CGMMV- or PepMV-infected NbBeclin1-silenced plants from control plants pretreated with mock (two-sided, **P < 0.01). **g**, **h** Symptoms of CGMMV (**g**) or PepMV (**h**) infected plants at 21 dpi. The plants were pre-inoculated with buffer (mock), TRV-GUS, or TRV-NbBeclin1 for 7 days. Mock inoculated with buffer, CGMMV inoculated with CGMMV, PepMV inoculated with PepMV

The conserved GDD motif is present in almost all RdRps from diverse plant and animal viruses. In addition to TuMV NIb (Fig. 2), NbBeclin1 also interacted with NIbs from tested potyviruses including PPV, SMV, and TEV but not with their corresponding GDD deletion mutants (Supplementary Fig. 8g). To address whether NbBeclin1 also targets viral RdRps of viruses other than potyviruses, we cloned RdRps from *Cucumber green mottle mosaic virus* (CGMMV) of the *Tobamovirus* genus, and *Pepino mosaic virus* (PepMV) of the *Potexvirus* genus (Fig. 9a, b). NbBeclin1 strongly interacted with both RdRps (Fig. 9c). In both cases, the interacting domain was mapped to the GDD-containing RdRp2 domain and deletion of the GDD motif abolished the interaction (Fig. 9c). Co-IP and BiFC confirmed the data obtained from the Y2H assay (Fig. 9d, e).

The TRV VIGS vector was used to silence *NbBeclin1*- and the *NbBeclin1*-silenced plants were challenged with CGMMV and PepMV. The increased viral RNA levels of CGMMV or PepMV were detected in the inoculated leaves at 3 dpi and the upper systemically infected leaves at 14 dpi (Fig. 9f). Consistently, the *NbBeclin1*-silenced plants showed enhanced susceptibility to CGMMV and PepMV with more severe symptoms (Fig. 9g, h).

## Discussion

Successful viral infection results from molecular interplays between the invading virus and the host^41–44^. Viral infection activates autophagy, as shown during infection by many animal viruses^15–19,45,46^, and activated autophagy selectively degrades viral proteins as an important part of host immunity. A similar picture is also emerging in plants. As mentioned earlier, two recent publications have provided strong evidence that two ATGs, i.e., NBR1 and ATG8 can function as cargo receptor proteins to selectively interact with viral proteins to mediate their degradation and defend infection from two DNA viruses^21,22^. In this study, we found that infection by TuMV, a plant RNA virus, also significantly upregulated the autophagy pathway in plants (Fig. 1 and Supplementary Fig. 1). A TuMV RNA replication mutant, TuMV-ΔGDD, which could express all TuMV proteins including the replication-defective NIb, failed to activate autophagy and induce the high expression of *ATG*s (Fig. 1). Transient overexpression of individual potyviral proteins alone, including the wild-type NIb, failed to induce autophagy (Supplementary Fig. 2). These data lead us to suggest that autophagy induction is likely triggered by the viral replication process or viral RNA accumulation rather than individual potyviral proteins. We further found that Beclin1 interacted exclusively with NIb to mediate NIb degradation likely via the autophagy pathway (Figs. 2–5 and 7 and Supplementary Figs. 5–7, 11). Silencing of *Beclin1* enhanced viral replication and symptom development, whereas overexpression of *Beclin1* suppressed viral infection (Fig. 8). These data raise a possibility that Beclin1 is a selective receptor against TuMV infection. As a core component of the class III PI3K complex, Beclin1 may regulate autophagy by serving as a scaffold or interaction hub for interacting with diverse protein partners^47^. Genetic lesion studies demonstrate that Beclin1/ATG6 is a conserved requirement in autophagy in plants, humans, and other eukaryotes^48^. Consistent with this study, in their seminal work, Liu and colleagues reported that silencing of *Beclin1* and several other *ATG*s enhances infection by TMV and autophagy is required for the timely HR to restrict TMV infection^20^. It is possible that Beclin1-mediated autophagy inhibits TMV infection via the interaction of Beclin1 and TMV RdRp, and autophagy regulates HR by a different mechanism. In mammalian cells, several viral proteins interact with Beclin1, which has been suggested to be an autophagy counteracting mechanism to promote viral infection^49–52^. It is not clear if any autophagy-mediated degradation also occurs on these Beclin1-interacting viral proteins.

Viral RdRp is absolutely required for positive-sense RNA virus replication and all viral RdRps have a conserved GDD motif^53^. We found that Beclin1 interacted with the RdRp domain of RdRps from all six test RNA viruses and the highly conserved motif GDD was essential for the interaction (Figs. 2, 6, and 9; Supplementary Fig. 8g). Overexpression of the partial NbBeclin1-C fragments (C1 or C4), losing the ability to mediate autophagy-dependent NIb degradation but competent for the interaction with GDD, inhibited TuMV replication (Fig. 7e), suggesting that Beclin1 binding to viral RdRp not only mediates its degradation but also suppresses its replication activity independent of Beclin1-mediated degradation.

ATG8 is a ubiquitin-like protein^54^ central to the autophagy pathway by binding to numerous cargo receptors and decorating autophagosomes^5^. In this study, we found that Beclin1 interacted with ATG8a but not ATG8f, Beclin1-mediated NIb degradation required ATG8a (Figs. 3–5; Supplementary Figs. 7 and 9), and the Beclin1-NIb complex or the Beclin1-NIb-VRC complex were co-localized with autophagosomes (Figs. 3 and 4). Beclin1 interacted with ATG8a using Beclin1’s AIM motif at the N terminus (Fig. 6b, c and Supplementary Fig. 9) and with NIb at its C-terminal region (Fig. 6a and Supplementary Fig. 8a–e). Thus it is very attempting to suggest that Beclin1 is a bridge to guide NIb to autophagosomes for degradation. Moreover, the AIM motif of Beclin1 was necessary for the formation of autophagosomes and induction of autophagy flux (Fig. 6d–f and Supplementary Figure 11a–c), supporting Beclin1 may play roles as an autophagy substrate and a functional receptor.

Silencing and overexpression of *ATG8a* affected plant susceptibility to infection in the same ways as *Beclin1* (Supplementary Fig. 16). These findings revealed the biological importance of ATG8a in Beclin1-mediated selective autophagy in suppressing viral infection. However, knockdown of *ATG8f* did not significantly affect Beclin1-mediated NIb degradation and viral infection (Supplementary Figs 7 and 13). The fact that Beclin1 binds to NbATG8a rather than NbATG8f suggests that ATG8 family proteins may selectively bind to different autophagy receptors.

In addition to ATG8, we also examined the possible involvement of several other core autophagy proteins. Silencing or knockdown of *PI3K* or *VPS15* promoted TuMV infection in *N*. *benthamiana* or Arabidopsis plants (Supplementary Fig. 17), which is not surprising as PI3K and VPS15, together with Beclin1, form the PI3K complex that initiates autophagy^1–5^. Knockout of another core autophagy gene *ATG2*, known to be involved in the early steps of autophagosome biogenesis, also increased plant susceptibility to TuMV infection (Supplementary Figs 13 and 18). In contrast, silencing of *ATG5* or *ATG7* did not affect viral infection (Supplementary Figs 13 and 18). We further found that it is not ATG5/ATG7 but ATG2 that is essential for the functionality of Beclin1-mediated degradation of NIb (Supplementary Fig. 7). The fact that NbBeclin1-mediated NIb degradation is dependent on ATG2 and independent of ATG5/ATG7 leads us to suggest that there is an alternative autophagy pathway in plants. In mammalian cells, it has been shown that *ATG5* or *ATG7* deficiency fails to block the formation of autophagosomes/autolysosomes for autophagy-mediated protein degradation upon induction by certain stressors, and thus macroautophagy can take place via at least two pathways: an ATG5/ATG7-dependent conventional pathway and an ATG5/ATG7-independent non-canonical pathway^55^. Our data raise the possibility that this might also hold true for plants and further study is needed to clarify this non-canonical pathway in plants.

Supporting the concept of the antiviral role of autophagy in potyviral infection, Nakahara and colleagues showed that the calmodulin-like protein in *N*. *tabacum*, rgs-CaM, counterattacks TEV HC-Pro and other VSRs by binding to the dsRNA-binding domain to inhibit its RNAi capacity and the resulting interaction complex is degraded by the autophagy pathway to enhance host antiviral defense^56^. Conversely, rgs-CaM in *N*. *benthamiana* suppresses RNA silencing and promotes geminivirus infection by autophagy-dependent degradation of suppressor of gene silencing 3 (SGS3), a key component of the RNA silencing pathway^57^. Recently, we also found that VPg, the second potyviral VSR, interacts with SGS3 and mediates the degradation of SGS3 and its intimate partner RNA-dependent RNA polymerase 6 (RDR6) via the ubiquitin–proteasome and autophagy pathways, suggesting a possible mechanism by which VPg sabotages host antiviral RNA silencing to promote viral infection^58^. The proviral role of autophagy has also been demonstrated during viral infection in animals^15^. For example, knockdown of *ATG7* or *Beclin1* inhibits the replication of *Hepatitis C virus* (HCV), demonstrating that they are proviral factors in HCV infection^59,60^. These data suggest that, during the co-evolutionary arms race, viruses have also developed strategies to subvert autophagy for their own benefit. Obviously, this increases the complexity of outcomes of induced autophagy in response to viral infection.

**Fig. 10:**
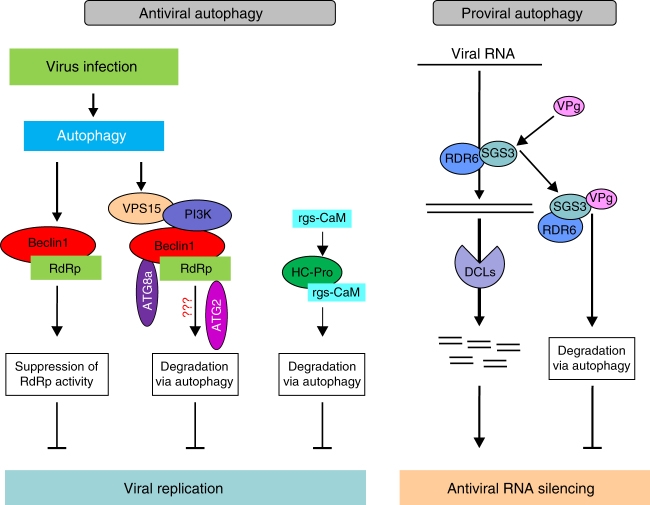
Proposed model for the possible roles of autophagy in the potyviral infection. Infection by positive-sense RNA viruses activates autophagy in plant cells. Beclin1 (ATG6) binds to the GDD motif of the viral RNA-dependent RNA polymerase (RdRp) to inhibit virus replication, which is independent of the autophagy pathway. Beclin1 may also serve as a cargo receptor to interact with the viral RdRp and target the RdRP-containing virus replication complex via the interaction of Beclin1 with other autophagy proteins (e.g., ATG8a) to autophagosomes for degradation. The mechanic details leading to the autophagy-mediated degradation remain to be fully understood. Simultaneously, HC-Pro, a virulence factor and the major potyviral VSR, is hijacked by host rgs-CaM to induce its degradation by the autophagy pathway^56^. Meanwhile, the second potyviral VSR, VPg, mediates the degradation of RNA silencing components SGS3 and RDR6 via the autophagy pathway to suppress antiviral RNA silencing^58^

Based on the discussion above, we propose a model summarizing the possible role of autophagy in potyviral infection (Fig. 10). Viral infection activates autophagy, leading to the accumulation of high levels of ATGs. Beclin1 interacts with NIb at the GDD motif and directly suppresses its RdRp activity to restrict viral infection. The Beclin1–NIb complex is targeted for autophagic degradation possibly through the interaction of Beclin1 and ATG8a via AIM. In addition, the activated autophagy pathway also targets two VSRs, e.g., HC-Pro and VPg^56,57^. As an antiviral mechanism, the former is recognized by rgs-CaM to induce autophagy-dependent degradation^56^. The latter binds to SGS3 to mediate the degradation of SGS3 and RDR6 via autophagy, which promotes viral infection^57^. The mechanism(s) by which autophagy is recruited is not clear. Based on this study, targeting viral RdRp by Beclin1 is likely a general antiviral mechanism in plants. This opens up a novel exciting avenue for the control of plant RNA viruses through upregulation of *Beclin1* and/or *ATG8a* or overexpression of the GDD-binding domain of Beclin1. It would be interesting to determine whether Beclin1 also interacts with RdRps of RNA viruses in mammalian cells and confers broad-spectrum resistance to viral infection therein.

## Methods

### Plant materials and growth conditions

*N*. *benthamiana* and Arabidopsis ecotype Col-0 seedlings were potted in soil and placed in an insect-free growth chamber under constant conditions of 60% relative humidity and a day/night regime of 16 h in the light at 22 °C followed by 8 h at 18 °C in the dark. The transgenic H2B-RFP line was a gift from Michael M. Goodin (University of Kentucky, USA). Arabidopsis mutants were obtained from Arabidopsis Stock Centre (ABRC at Ohio State University, Columbus, Ohio, USA): atg6 (SALK_109281), atg8a (SALK_045344c), vps15 (SALK_004719), pi3k (CS355912), atg2 (SALK_006994C), atg5 (CS39993), and atg7 (CS39995). AtATG6 overexpression (35S:Myc-AtATG6) and AtATG8a overexpression (35S:Myc-AtATG8a) transgene Arabidopsis seeds were obtained by using the flower-dip method^61^.

### Viral strains

The recombinant viruses TuMV, TuMV-GFP, TuMV-ΔGDD, TuMV-CFP-NIb-6K2-mCherry, TuMV-6K2-mCherry, and PPV-GFP-mCherry were previously described^35,36,62^, and PePMV and CGMMV were isolated from tomato and cucumber in a greenhouse in Ontario, Canada.

### Plasmid construction

GenBank accession numbers of genes and viral sequences analyzed in this study are as follows: *NbBeclin1* (AY701316), *NbPI3K* (KX120977), *NbVPS15* (KU561371), *NbATG2* (KU561373), *NbATG9* (KX369399), *NbATG3* (KX369396), *NbATG5* (KX369397), *NbATG7* (KX369398), *NbATG8a* (KX120976), *NbATG8f* (KU561372), *AtATG6* (NM_202746), TuMV (NC002509), TEV (NC001555), SMV (EU871724), PPV (KP998124), *NbActin* (AY179605), and *AtActinII* (AT3G18780). Gateway technology (Invitrogen, Burlington, Ontario, Canada) was used to generate all the plasmid constructs used in this study, unless otherwise stated. Gene sequences were amplified by PCR using Phusion^®^ High-Fidelity DNA Polymerase (New England Biolabs, Pickering, ON, Canada) for cloning purposes. GoTaq^®^ Flexi DNA Polymerase (Promega, Madison, WI, USA) was employed for regular PCR analysis. The full-length TuMV P1, HC-Pro, P3, P3N-PIPO, 6K1, CI, 6K2, VPg, NIa-Pro, NIb and CP, PPV NIb, SMV NIb, and TEV NIb were cloned previously^30,32,33,62–64^. *NbBeclin1*-, *NbATG8a*-, and *NbATG8f*-coding sequence were generated from cDNA derived from *N*. *benthamiana* leaves and *AtATG6*-coding sequence was generated from cDNA derived from Arabidopsis leaves (primers are listed in Supplementary Table 1). The GDD mutants of TuMV NIb and PPV NIb were cloned from the infectious clone TuMV-ΔGDD^30^ and PPV-ΔGDD^36^. Coding cDNA sequences of RdRp and its domains Met, Hel, and RdRp2 of CGMMV (GenBank accession: AB015146) and PepMV (KY031324) were cloned by RT-PCR. CGMMV RdRp2-ΔGDD, PepMV RdRp2-ΔGDD, TEV NIb-ΔGDD, and SMV NIb-ΔGDD were cloned by overlapping PCR using the specific primers (Supplementary Table 1). One AIM that matches the consensus amino acid sequence X-3X-2X-1-W/F/Y-X1X2-L/I/V was located in the N-terminal of NbBeclin1: EESFVVL. The mutant of AIM motif (NbBeclin1-N^ΔAIM^) was produced by replacing EESFVVL with EESAVVA using overlapping PCR primers (Supplementary Table 1). The resulting DNA fragments were purified and transferred into the entry vector pDONR221 (Invitrogen) by recombination using BP Clonase^®^ (Invitrogen) following the standard instruction by the supplier^65^. Insertions in the resulting pDONR clones were verified by DNA sequencing. For Y2H assays, inserts of the resulting intermediate pDONR221 clones were further transferred into modified Gateway-compatible vectors pGADT7-DEST (prey) or pGBKT7-DEST (bait)^66^ by recombination using LR Clonase® (Invitrogen) to yield pGAD-NbBeclin1, -AtATG6, -NbBeclin1-N, -NbBeclin1-C, NbBeclin1-N^ΔAIM^, and pGBK-P1, -HC-Pro, -P3, -P3N-PIPO, -6K1, -CI, -6K2, -VPg, -NIa-Pro, -NIb, -NIb-N, -NIb-M, -NIb-C, NIb-ΔGDD, -CP, -PPV NIb, -PPV NIb-ΔGDD, -SMV NIb, SMV NIb-ΔGDD, -TEV NIb, -TEV NIb-ΔGDD, -NbATG8a, -NbATG8f, CGMMV RdRp, CGMMV Met, CGMMV Hel, CGMMV RdRp2, CGMMV RdRp2- ΔGDD, PepMV RdRp, PepMV Met, PepMV Hel, PepMV RdRp2, and PepMV RdRp2-ΔGDD, respectively. For the BiFC assay and transient expression analysis in plant cells, the full-length coding sequences of NbBeclin1, AtATG6, NbATG8a, NbATG8f, NIb, P3N-PIPO, CGMMV RdRp, CGMMV Met, CGMMV Hel, CGMMV RdRp2, PepMV RdRp, PepMV Met, PepMV Hel, and PepMV RdRp2, the partial coding sequences of NbBeclin1 (NbBeclin1-N and NbBeclin1-C) and NIb (NIb-N, NIb-M and NIb-C), and the mutants of NbBeclin1^ΔAIM^, NbBeclin1-N^ΔAIM^, NIb-ΔGDD, CGMMV RdRp2-ΔGDD, and PepMV RdRp2-ΔGDD were introduced into the BiFC vectors pEarleygate201-YN or pEarleygate202-YC^66^ or binary destination vectors pEarleyGate101 (YFP in the C terminal), pEarleyGate104 (YFP in the N terminal), pEarleyGate102 (CFP in the C terminal)^67^, or pBA-Flag-Myc4 (Myc tag in the N terminal)^68^ to generate the corresponding plant expression vectors, respectively. To construct a TRV-based recombinant VIGS vector containing *NbBeclin1*, *NbATG8a*, *NbATG8f*, *NbPI3K*, *NbVPS15*, *NbATG2*, *NbATG5*, or *NbATG7*, a partial fragment of each gene was generated by PCR amplification with the respective primer pair (Supplementary Table 1) and then cloned into the pTRV2 vector using different restriction enzyme sites listed in Supplementary Table 1.

### Agroinfiltration and viral inoculation

For transient expression analysis in *N*. *benthamiana* leaves, constructs were generated in Gateway-compatible binary vectors and transformed into *A*. *tumefaciens* strain GV3101 via electroporation. The 4–5 leaf stage wild-type *N*. *benthamiana* or H2B transgene *N*. *benthamiana* plants were used for *Agrobacterium-*mediated transient expression with minor modifications of our previous procedures^62–64^. For viral infection analysis, *Agrobacterium* cultures carrying different viral infectious virus clone(s) were infiltrated into *N*. *benthamiana* or Arabidopsis leaves and inoculated plants were photographed with a Canon 400D digital camera at different time periods. The negative control plants were agroinfiltrated with infiltration buffer as mock inoculations. The TuMV infection assay was repeated at least three times. For the TRV-VIGS assay, *Agrobacterium* cultures harboring pTRV1 and pTRV2-VIGS (TRV2-GUS, TRV2-NbBeclin1, TRV2-NbATG8a, TRV2-NbATG8f, TRV2-NbPI3K, TRV2-NbVPS15, TRV2-NbATG2, TRV2-NbATG5, or TRV2-NbATG7) were re-suspended in infiltration buffer (10 mM MgCl_2_ and 10 mM MES (pH5.6 and 100 μM acetosyringone) and mixed at a 1:1 ratio. After 3 h incubation at room temperature, the mixed *Agrobacterium* cultures were infiltrated into leaves of *N*. *benthamiana* plants at the 4–5 leaf stage. *N*. *benthamiana* plants infiltrated with TRV-based VIGS vectors targeting phytoene desaturase as an indicator, and silenced phenotype appeared in the upper leaves at 7 dpi. TRV-GUS- or TRV-VIGS-treated plants were agroinfiltrated with TuMV-GFP, CGMMV, or PepMV infectious clone at 7 dpi.

### Y2H, BiFC, subcellular localization

Y2H was performed following the Clontech yeast protocol handbook. In brief, yeast cells (strain Y2H Gold, Clontech catalog number: 630498) carrying the co-transformed plasmids were plated onto a selective medium lacking tryptophan and leucine (SD/-Trp-Leu) to confirm the right transformation and a high-stringency selective medium lacking tryptophan, leucine, histidine, and adenine (SD/-Trp-Leu-His-Ade) to analyze the interaction. BiFC and subcellular localization experiments were performed as described^32,35,62–64^. At 36–72 h after infiltration, 1–2 cm^2^ leaf explants were excised for examining fluorescence in epidermal cells by confocal microscopy (Leica Microsystems, Wetzlar, Germany), equipped with a 63× water-corrected objective in multitrack mode. Light emitted at 680–720 nm was used to record chlorophyII autofluorescence. CFP was excited at 458 nm and captured at 470–500 nm, YFP was excited at 514 nm and captured at 565–585 nm, GFP was excited at 488 nm and captured at 510–550 nm, and mCherry or RFP was excited at 543 nm and captured at 590–630 nm. The sequential scanning mode was applied for co-imaging of different fluorescent proteins. Collected images were analyzed with the Leica Application Suite for Advanced Fluorescence (LAS AF, version 2.35) software (Leica Microsystem). Unless otherwise indicated, confocal images reported in the same figure panel were taken and processed with the same settings except otherwise indicated.

### Protoplast isolation and TuMV replication assay

Mesophyll protoplasts were isolated and transfected essentially as described^69^. In brief, mesophyll protoplasts were prepared from 4-week-old wild type, *atg6* (SALK_109281), 35S:Myc-AtATG6, *atg8a* (SALK_045344c), or 35S:Myc-AtATG8a Arabidopsis leaves or TRV-GUS-, TRV-NbBeclin1-, and TRV-NbATG8a-silenced newly emerged *N*. *benthamiana* leaves as described previously^69^. About 1×10^5^ protoplasts were transfected with 20 µg pCambia2300-TuMV-GFP plasmid in 40% PEG 4000 in 0.8 M mannitol and 1 M CaCl_2_ at room temperature for 20 min. Transformed protoplasts were then washed and resuspended in W5 buffer (154 mM NaCl, 125 mM CaCl_2_, 5 mM KCl, and 2 mM MES, pH 5.7) and incubated for the virus replication. At 16, 24, and 48 h posttransfection, the protoplasts were harvested for the extraction of RNA. The protoplast transfection assay was repeated at least three times, and one representative result was used to plot a histogram.

### RNA extraction and qRT-PCR analysis

Total RNA was extracted from mock (*Agrobacterium*-carrying empty vector)-, TuMV-ΔGDD-, or TuMV-infiltrated *N*. *benthamiana* leaves or *Agrobacterium*-carrying empty vector (Vec), NIb-YFP, Myc-NbBelin1 or Myc-NbBeclin1- and NIb-YFP-infiltrated *N*. *benthamiana* leaves or TRV-VIGS-treated *N*. *benthamiana* leaves or TuMV-GFP-, CGMMV-, or PepMV-infiltrated TRV-VIGS-treated *N*. *benthamiana* leaves or mock, TuMV-ΔGDD, TuMV, TuMV-GFP, CGMMV, or PepMV systemically infected Arabidopsis, *N*. *benthamiana*, or TRV-VIGS-treated *N*. *benthamiana* leaves at different time periods using the RNeasy Plant Mini Kit (Qiagen) and treated with DNase I following the manufacturer’s instructions. cDNA synthesized from reverse transcription of RNA samples was used to determine the mRNA expression levels of target genes as well as for quantifying TuMV accumulation levels at the date indicated. *NbActin* or *AtActin II* was used as an internal control for *N*. *benthamiana* and Arabidopsis, respectively. First-strand cDNA was synthesized from 1 µg total RNA by using Oligo(dT)12–18 primer or specific primers and SuperScript® III reverse transcriptase (Invitrogen) following the recommended protocol. A list of primers used in this study is provided (Supplementary Table 1). qRT-PCR was conducted and analyzed as described previously^57,70^.

### Chemical treatments and TEM

Phosphate-buffered saline containing dimethyl dulfoxide (DMSO; control) or an equal volume of DMSO with 5 mM 3-MA, 100 μM E64d, or 1 μM Con A (Sigma) for inhibition of autophagy was infiltrated into leaves 8–12 h before samples were collected. For TEM observation, detailed information has been described previously^57^. Upper non-inoculated leaves of *N*. *benthamiana* plants infected with buffer (mock), TuMV, and TuMV-ΔGDD at 7 dpi or agro-infiltrated *N*. *benthamiana* leaves with mock, NbBeclin1, NbBeclin1^ΔAIM^, NbBeclin1-N, NbBeclin1-N^ΔAIM^, or NbBeclin1-C at 60 hpi were pretreated with 10 mM 3-MA for 8 h and then were cut into small pieces (1 × 4 mm^2^). The treatments and the examination of the sampled tissues were performed as described^57^.

### Immunoblotting and immunoprecipitation

Total protein was extracted from infiltrated leaf patches or mock-, TuMV-, and TuMV-ΔGDD-infiltrated or systemically infected *N*. *benthamiana* leaves as described previously^32,33,57^. Immunoblotting was performed with the primary rabbit polyclonal antibodies: GFP polyclonal antibody (1:5000; catalog number: ab6556), Myc polyclonal antibody (1:5000; catalog number: ab9106), and Beclin1 polyclonal antibody (1:1000; catalog number: ab62557) obtained from Abcam (Massachusetts, USA), ATG8 polyclonal antibody obtained from Millipore (1:2000; catalog number: ABC974, Millipore, Massachusetts, USA), and HA polyclonal antibody obtained from Sigma (1:10,000; catalog number: H6908, Sigma-Aldrich Canada Co., Oakville, ON, Canada), followed with goat anti-rabbit (1:10,000; catalog number: ab6721) secondary antibody conjugated to horseradish peroxidase (Abcam, Massachusetts, USA). Blotted membranes were washed thoroughly and visualized using chemiluminescence according to the manufacturer’s protocol (ECL; GE Healthcare). Immunoprecipitation was done on protein exacts from *N*. *benthamiana* leaves at 2 dpi by using GFP-Trap®_MA (Chromotek) according to the manufacturer’s instructions with some modifications. Briefly, about 1 g of leaf tissues were ground in liquid nitrogen and extracted with 2 ml lysis buffer [10% glycerol, 25 mM Tris HCl (pH 7.5), 1 mM EDTA, 150 mM NaCl, 2% w/v PVPP, 10 mM DTT, 1× EDTA-free protease inhibitor cocktail (Roche), 0.1% IGEPAL CA-630 (Sigma)]. After incubation on ice for 30 min with gentle shaking, the mixtures were centrifuged at 3000 × *g* at 4 °C for 10 min. The supernatants were then centrifuged with full speed at 4 °C for 10 min. Extracts were passed through a 0.45 µm filter and then incubated with GFP-Trap®_MA beads (ChromoTek) for 3 h at 4 °C with gentle shaking. The precipitations were washed four times with ice-cold immunoprecipitation buffer (10% glycerol, 25 mM Tris HCl (pH 7.5), 1 mM EDTA, 150 mM NaCl, 1× EDTA-free protease inhibitor cocktail (Roche), 0.1% IGEPAL CA-630) at 4 °C and analyzed by immunoblotting with Myc, HA, or GFP antibodies. Uncropped images of immunoblots are shown in Supplementary Figs. 19 through 26.

### Data availability

The authors declare that all data supporting the findings of this study are available in the manuscript and its Supplementary Information files or are available from the corresponding author upon request.

## Electronic supplementary material

Supplementary Information (PDF 4692 kb)
