# Supplementary material for: Beclin1 restricts RNA virus infection in plants through suppression and degradation of the viral polymerase
Source: Nat Commun. 2018 Mar 28;9:1268. doi: 10.1038/s41467-018-03658-2 (PMC5871769; doi:10.1038/s41467-018-03658-2)
Supplement: Supplementary file 1 — Supplementary Information (PDF 4692 kb) [file 41467_2018_3658_MOESM1_ESM.pdf]

SUPPLEMENTARY INFORMATION

Beclin1 restricts RNA virus infection in plants through suppression and degradation of the viral polymerase

Li et al.

Supplementary Figures 1–26.

Supplementary Table 1.

Correspondence should be addressed to A.W. (e-mail: Aiming.Wang@AGR.GC.CA).

Supplementary Information

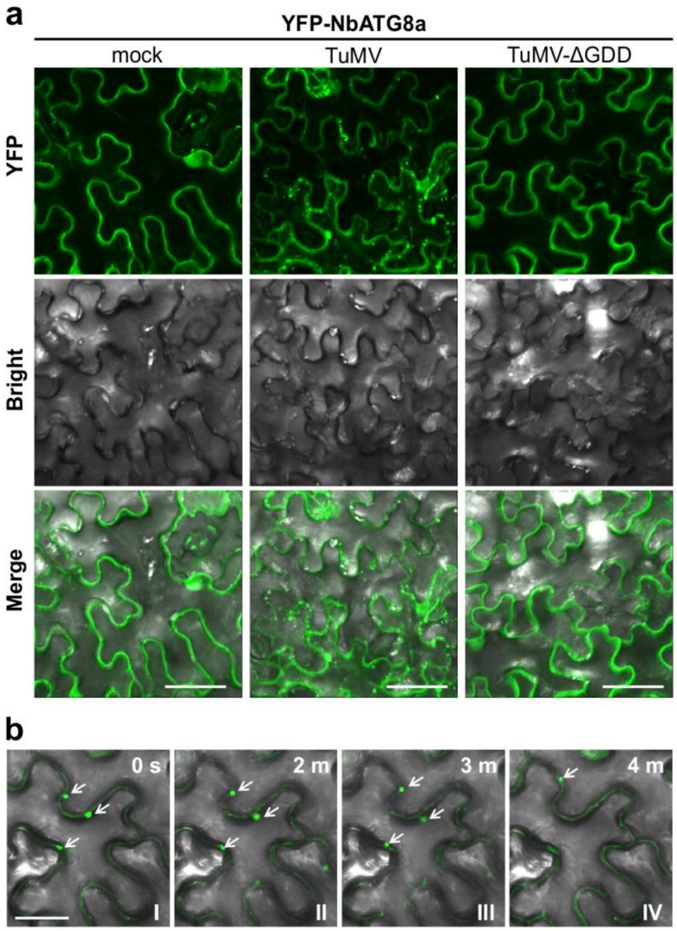

Supplementary Figure 1. Representative Z-stack and time-lapse images of YFP-NbATG8a in *N. benthamiana* leaf cells. (**a**) Confocal micrographs showing *N. benthamiana* leaf cells expressing YFP-NbATG8a together with an empty vector (mock), or a TuMV infectious clone (TuMV), or a TuMV replication-defective mutant (TuMV-ΔGDD) at 60 hours post infiltration (hpi). Bars, 50 μm. A maximum projection image was constructed from 40 consecutive images. Note: some quickly mobile autophagosomes formed a moniliform distribution in Z-stacks pictures in TuMV-infected leaves, as Z-stacks pictures contained a series of continued pictures, and the quickly mobile spots would show in these pictures repeatedly in different positions. (**b**) Time-lapse imaging of YFP-NbATG8a puncta in TuMV-infected *N. benthamiana* leaves. These four images were obtained at the same spots in the same leaf area photographed at four time points: 0, 2, 3 and 4 minutes (m). The movement of typical YFP-NbATG8a puncta is indicated by white arrows. Bars, 50 μm.

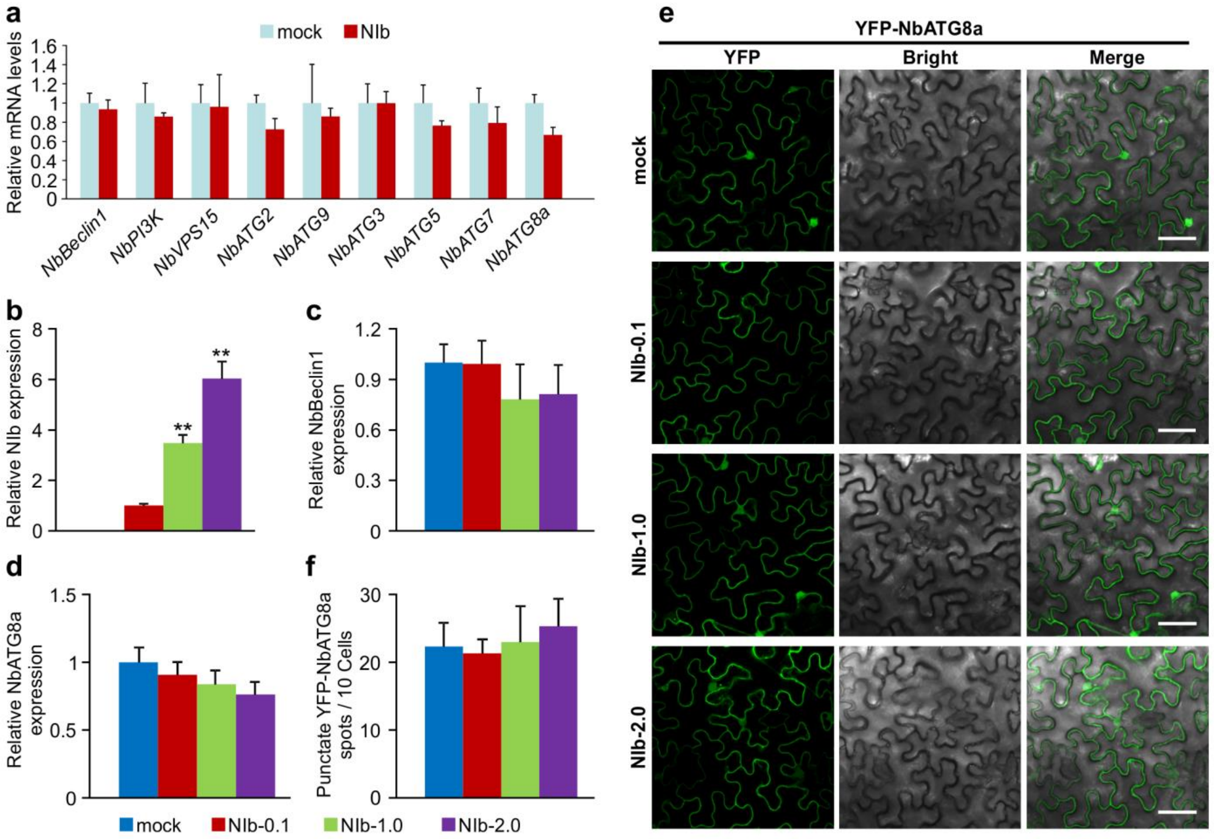

Supplementary Figure 2. Transient expression of individual potyviral proteins alone fails to induce autophagy. NIb was used as an example. (**a**) *N. benthamiana* leaves were infiltrated with agrobacterial cultures containing an empty vector (mock) or TuMV NIb (NIb). Total RNAs were extracted from infiltrated zones from three pools of plants at 60 hpi. Relative mRNA levels of *NbBeclin1*, *NbPI3K*, *NbVPS15*, *NbATG2*, *NbATG9*, *NbATG3*, *NbATG5*, *NbATG7* and *NbATG8a* were analyzed by qRT-PCR with specific primers. The values obtained from mock-infiltrated plants at 60 hpi were arbitrarily set as 1. The values ± standard deviation (SD) relative to the corresponding mock values are presented, and Error bars represent SD (n=3 biological replicates). **(b-d)** The effect of different expression levels of NIb (**b**) on the expression level of *NbBeclin1* (**c**) and *NbATG8a* (**d**). *N. benthamiana* leaves were infiltrated with different concentrations of agrobacterial culture carrying an empty vector (mock, OD_{600}=1.0) and NIb (NIb-0.1, OD_{600}=0.1; NIb-1.0, OD_{600}=1.0; NIb-2.0, OD_{600}=2.0). The relative expression levels were determined by qRT-PCR at 60 hpi. Error bars indicate standard deviation (SD). Double asterisks indicate a significant difference of NIb expression (Student's *t* test, $P < 0.01$) between NIb-0.1 and NIb-1.0 or NIb-2.0-inflitrated leaves. (**e**) Confocal images showing *N. benthamiana* leaf cells agroinfiltrated with YFP-NbATG8a together with mock and three different concentrations of NIb. Infiltrated *N. benthamiana* leaves were examined at 60 hpi. Bars, 50 µm. (**f**) The average number of YFP-NbATG8a spots per 10 cells. Infiltration experiments were repeated three times and 60 cells in total were counted for punctate spots. Values represent the mean spots± SD.

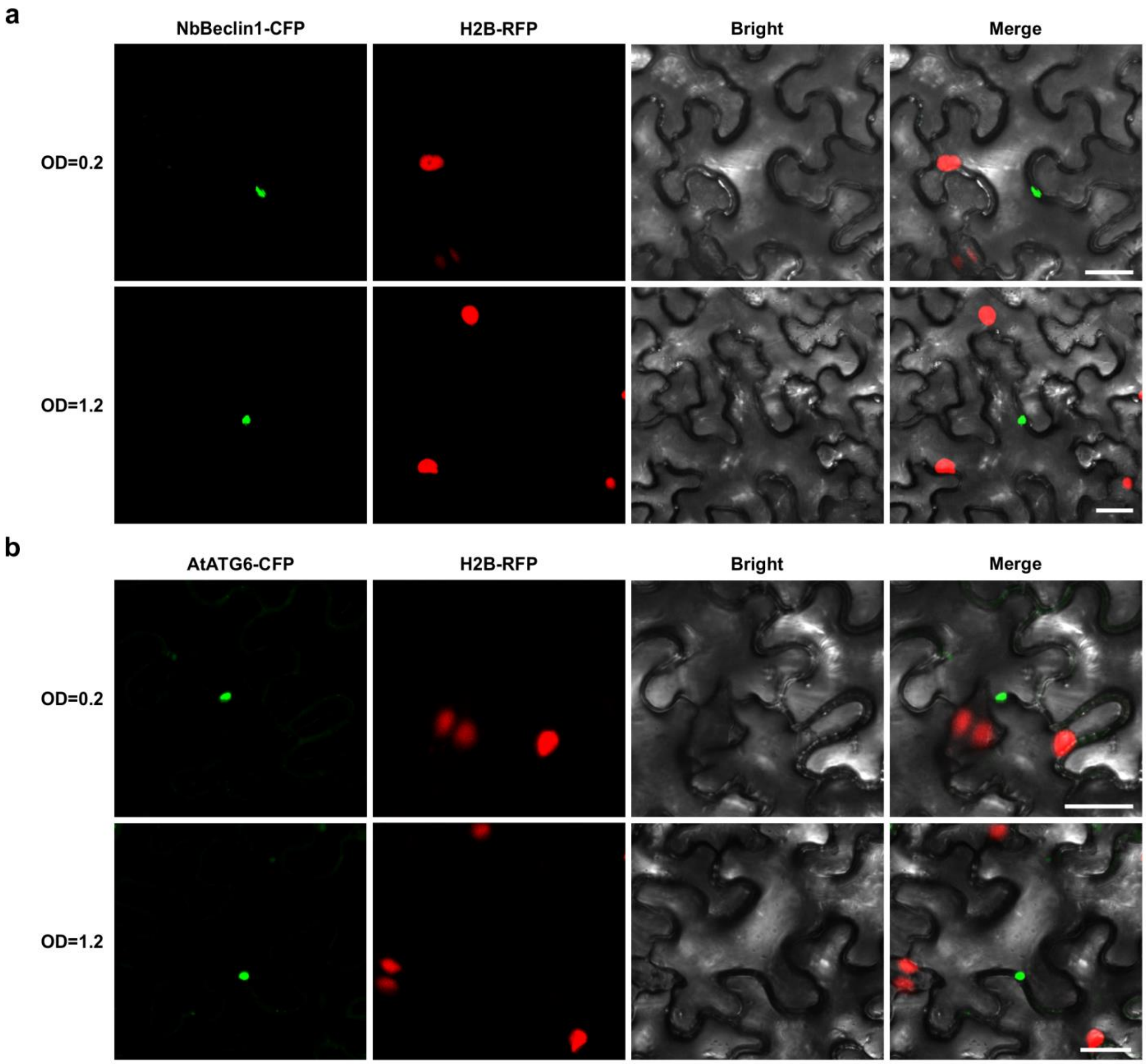

Supplementary Figure 3. Confocal micrographs showing cells from leaves of H2B-RFP transgenic *N. benthamiana* expressing NbBeclin1-CFP, AtATG6-CFP or NIb-YFP or co-expressing NIb-YFP and NbBeclin1-CFP. (**a, b**) H2B-RFP transgenic *N. benthamiana* leaves were infiltrated with agrobacterial cultures containing NbBeclin1-CFP (**a**) or AtATG6:CFP (**b**) (OD_{600}=0.2 or OD_{600}=1.2). The infiltrated leaf patches were observed by confocal microscopy at 48 hpi. Bars = 25 µm

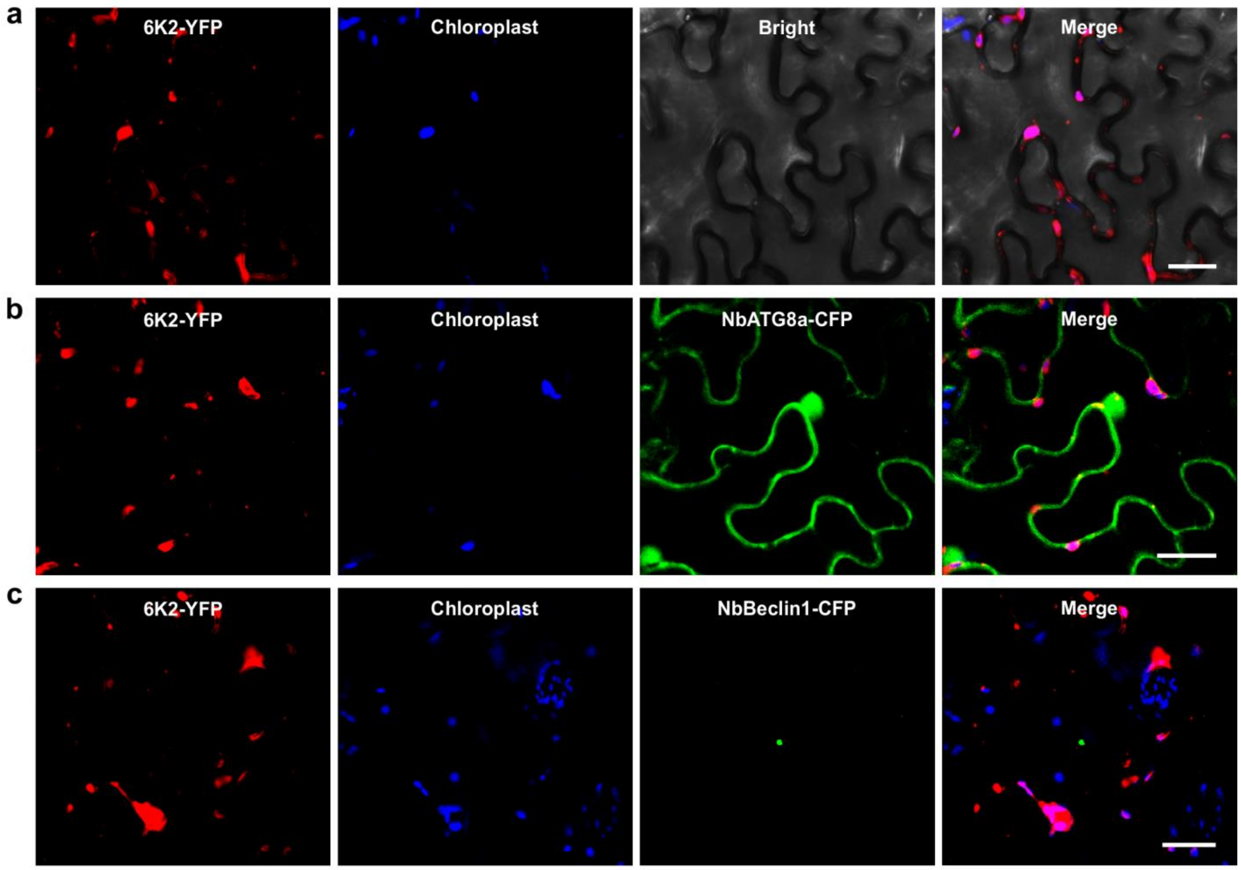

Supplementary Figure 4. NbATG8a or NbBeclin1 does not co-localize with TuMV 6K2-YFP in the absence of viral infection. (**a**) 6K2-YFP was transiently expressed alone in *N. benthamiana* leaf cells. (**b**) Transient co-expression of 6K2-YFP and NbATG8a-CFP. (**c**) Transient co-expression of 6K2-YFP and NbBeclin1-CFP. All confocal images in this figure were taken at 48 hpi. The pictures were obtained using the same setting. Bars = 25 µm.

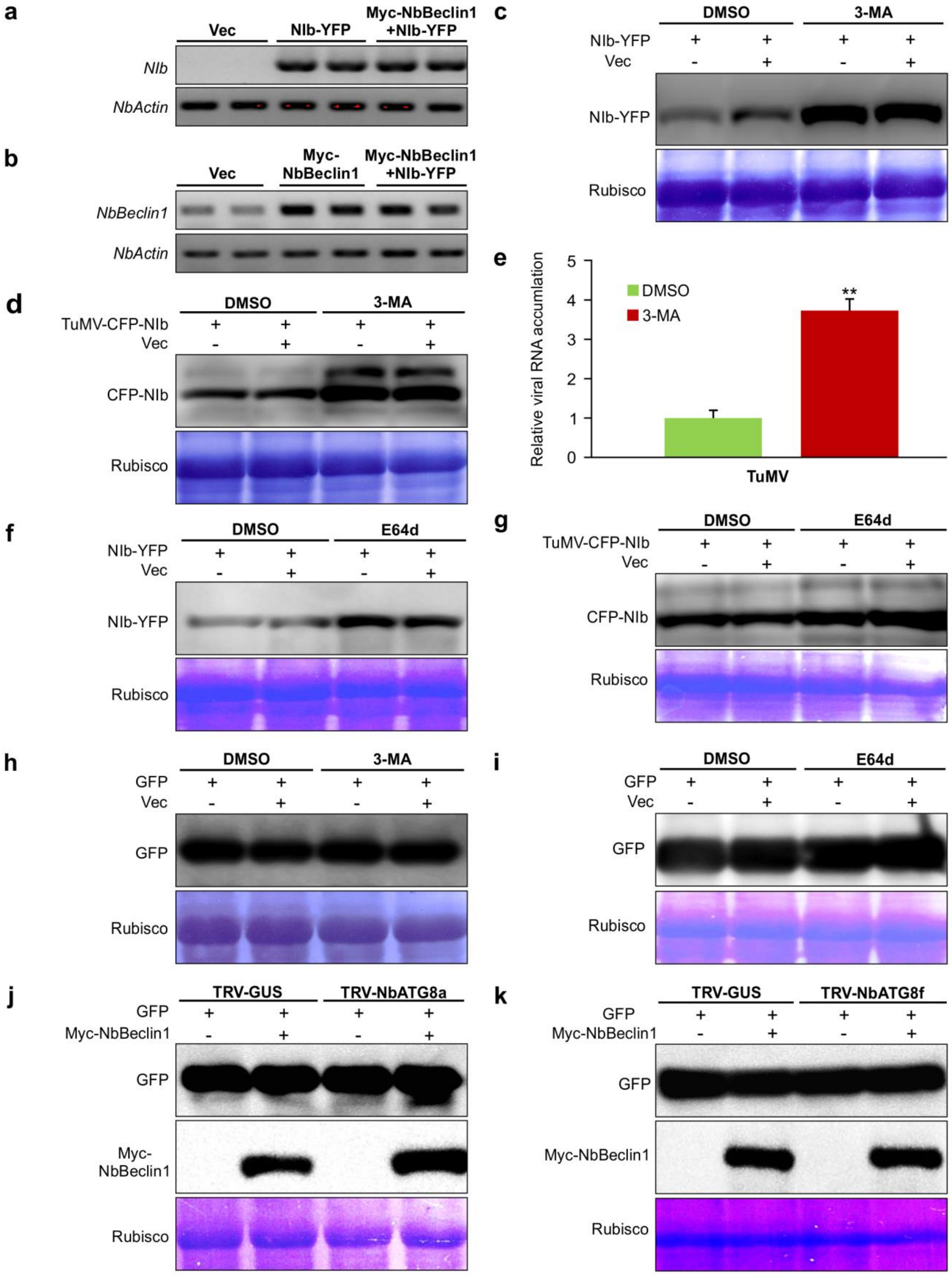

Supplementary Figure 5. Degradation of NIb and VRCs is sensitive to inhibitors of autophagy. (**a,b**) RT-PCR was used for analysis of *NIb* and *NbBeclin1* transcripts in *N. benthamiana* leaves agroinfiltrated with an empty vector (Vec), NIb-YFP, Myc-NbBeclin1 or

NIb-YFP together with Myc-NbBeclin1 at 48 hpi. The specific primers for *NIb*, *NbBeclin1* and *NbActin* are listed in Supplementary Table 1. *NbActin* expression was used as an internal control. (**c, d**) The effects of the autophagy inhibitor 3-MA on the accumulation level of NIb-YFP (**c**) or CFP-NIb derived from viral expression (**d**). Total proteins were isolated from *N. benthamiana* leaves agroinfiltrated with NIb-YFP alone or with Vec (**c**), or the TuMV-CFP-NIb infectious clone alone or with Vec (**d**) and, at 60 hpi, treated with DMSO (Control) or 3-MA (5 mM) for 10 h. Immunoblotting was performed with GFP antibody. (**e**) Quantification of TuMV RNA levels by qRT-PCR. Total RNA was extracted from *N. benthamiana* leaves inoculated with TuMV for 60 hpi, followed by treatment with DMSO or 3-MA for 10 h. The values show means ± SD (n=3 biological replicates) relative to the DMSO-treated plants. The data were analyzed using Student's *t* test and asterisks denote statistically significant difference between two treatments (two-sided, $^{**}P < 0.01$). **(f,g)** Total proteins were extracted from *N. benthamiana* leaves agroinfiltrated with NIb-YFP alone or with Vec (f), or TuMV-CFP-NIb alone or with Vec (**g**), followed by treatment with DMSO (control) or E64d (100 uM) for 10 h. (**h,i**) DMSO, 3-MA (5 mM) or E64d (100 uM) treated samples were harvested from plants agroinfiltrated with GFP alone, or with an empty vector (Vec). (**j,k)** The effect of silencing of *NbATG8a* or *NbATG8f* on NbBeclin1-mediated degradation of GFP. *N. benthamiana* plants were pre-treated with relevant TRV-recombinant vectors for 14 days and then infiltrated with GFP alone or with Myc-NbBeclin1. Immunoblotting was performed with GFP or Myc antibody. Coomassie Brilliant Blue R-250 (CBB)-staining of Rubisco large subunit serves as a loading control (**h-k**).

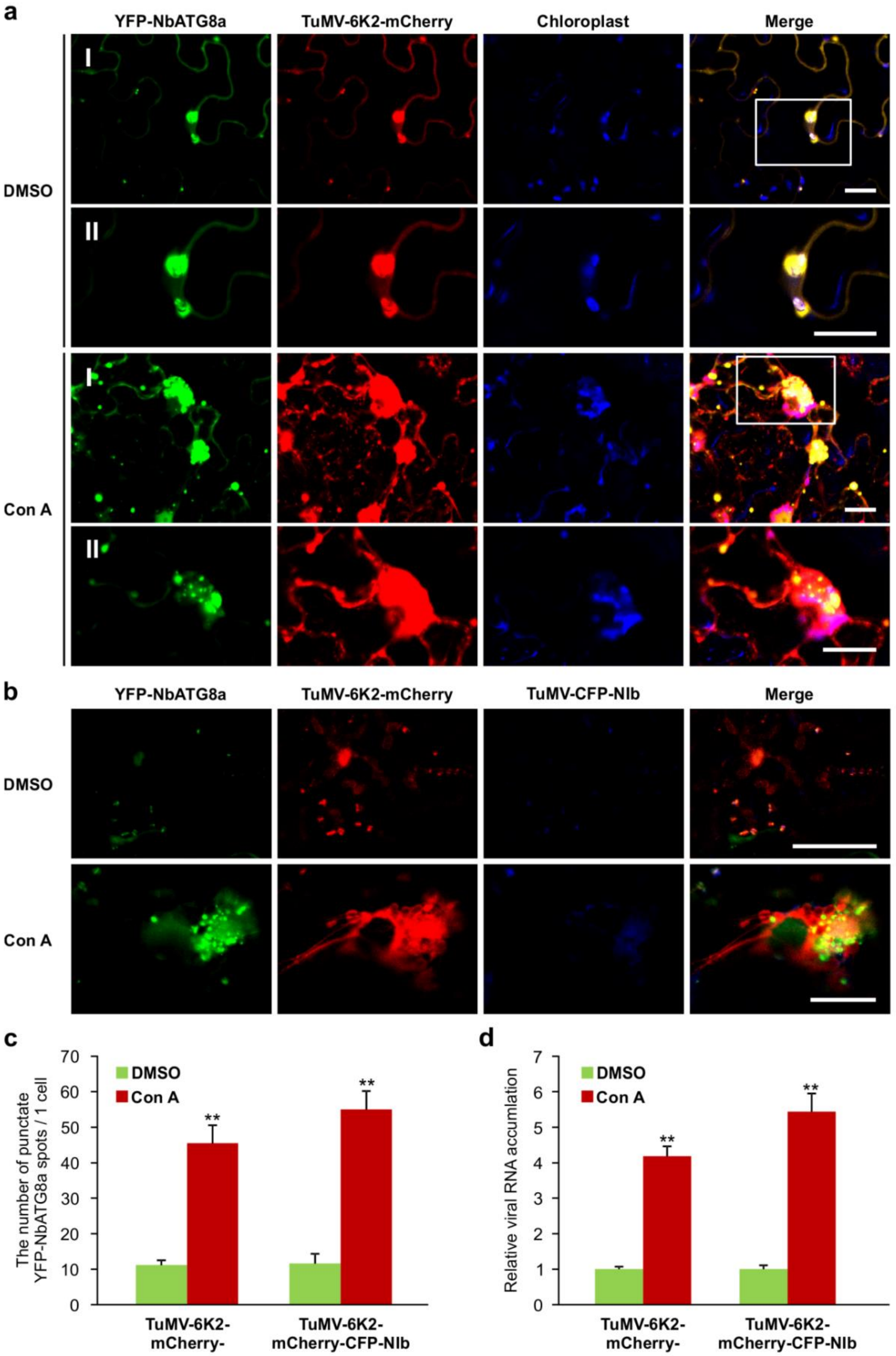
a
YFP-NbATG8a
TuMV-6K2-mCherry
Chloroplast
Merge
DMSO
Con A
I
II
b
YFP-NbATG8a
TuMV-6K2-mCherry
TuMV-CFP-NIb
Merge
DMSO
Con A
c
The number of punctate YFP-NbATG8a spots / 1 cell
DMSO
Con A
**
TuMV-6K2-mCherry-
TuMV-6K2-mCherry-CFP-NIb
d
Relative viral RNA accumulation
DMSO
Con A
**
TuMV-6K2-mCherry-
TuMV-6K2-mCherry-CFP-NIb

Supplementary Figure 6. Supplementary Figure 6. Concanamycin A (Con A) treatment increases the number of autophagosomes aggregated around VRC in the cytoplasm and facilitates viral RNA accumulations. (**a,b**) YFP-NbATG8a and NbBeclin1 were transiently expressed in leaf cells infected by TuMV-6K2-mCherry (a) or TuMV-6K2-mCherry-CFP-NIb (b) at 72 hpi. DMSO or Con A was applied to the infiltrated leaves 12 h before confocal observation. The corresponding region in the white box in the panel I of (a) is magnified in the panel II. Bars, 25 µm. Note: the confocal settings for the bottom images (panel II of the Con A treatment) were adjusted for a better view of autophagosomes and the VRC. (**c**) The number of autophagosomes was counted at 72 hpi. Infiltration experiments (a,b) were repeated three times and 60 cells in total were counted for punctate spots. Values represent the mean spots ± SD / 1 cell. (**d**) Quantification of TuMV RNA levels by qRT-PCR. Total RNA was extracted from *N. benthamiana* leaves infiltrated with as indicated in (a) and (b) at 72 hpi. The values are presented as means ± SD (n=3) relative to the treatment of DMSO. Asterisks indicate significant differences in the number of YFP-NbATG8a (c) or the fold change of viral RNA levels (d) between DMSO and Con A treatments. (c, d, Student's *t* test, two-sided, $^{**}P < 0.01$).

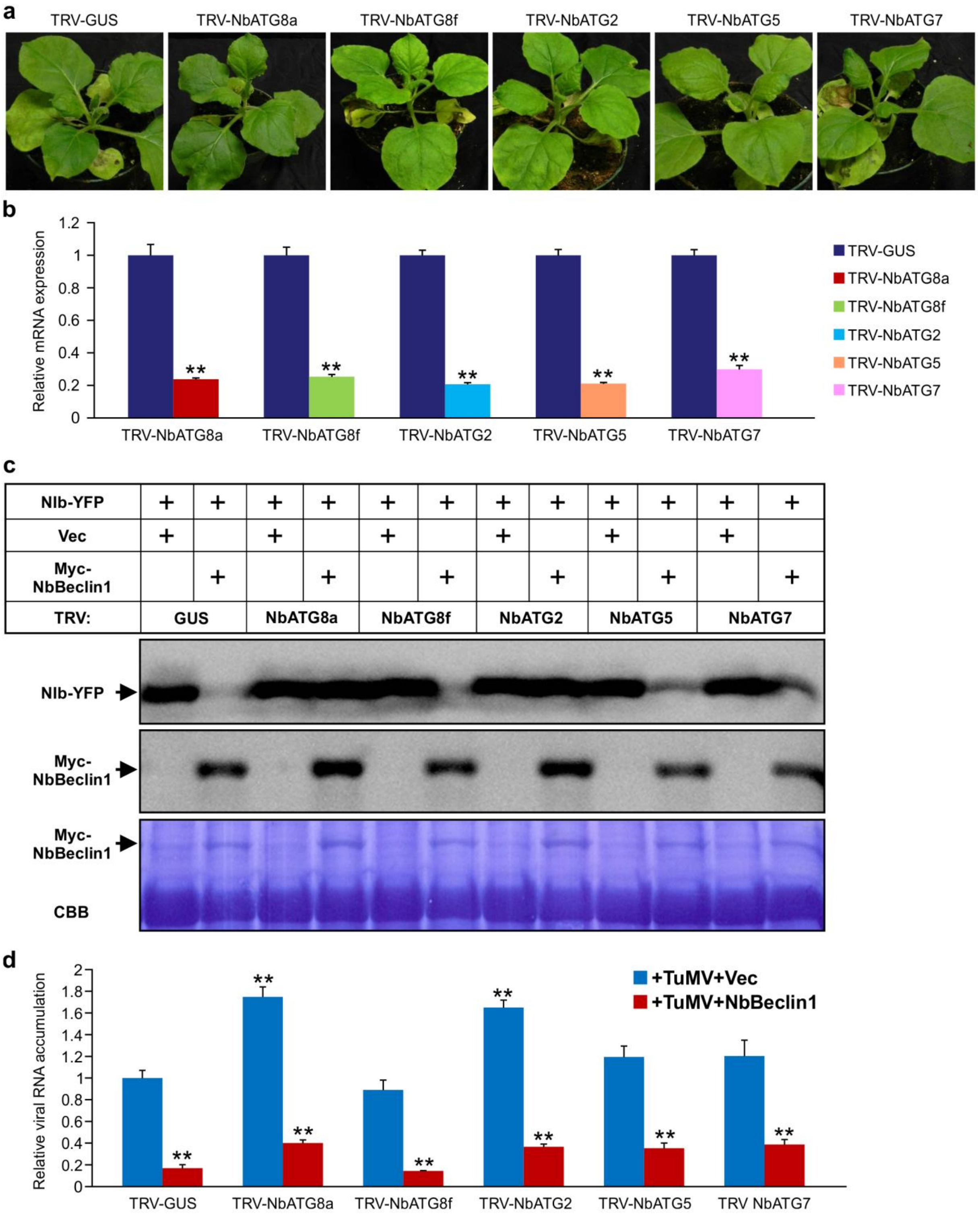

Supplementary Figure 7. Knock-down of *NbATG8f, NbATG5 or NbATG7* does not obviously block NbBeclin1-mediated the degradation of NIb. (a) The phenotype in TRV-GUS, TRV-NbATG8a, TRV-NbATG8f, TRV-NbATG2, TRV-NbATG5 or TRV-NbATG7-

treated plants at 14 dpi. A 265 bp cDNA fragment of *NbATG8a,* a 327 bp cDNA fragment of *NbATG8f,* a 307 bp cDNA fragment of *NbATG2,* a 313 bp cDNA fragment of *NbATG5,* or 324 bp cDNA fragment of *NbATG7* was cloned into RNA2 of the TRV VIGS vector. *N. benthamiana* plants at the 4-5 leaf stage were agroinfiltrated with pTRV1 and pTRV2-GUS, pTRV1 and pTRV2-VIGS (*NbATG8a*, *NbATG8f*, *NbATG2*, *NbATG5* or *NbATG7*). No striking developmental phenotypes were observed in plants treated with pTRV1 and pTRV2-VIGS, compared with plants treated with pTRV1 and pTRV2-GUS. **(b)** Silencing of *NbATG8a, NbATG8f, NbATG2, NbATG5 or NbATG7* was confirmed in newly emerged leaves 14 dpi by qRT-PCR. Double asterisks indicate significant differences (P < 0.01) between the two treatments (two-sided, Student's *t* test). (**c**) The effect of the silencing of *NbATG8a, NbATG8f, NbATG2, NbATG5 or NbATG7* on NbBeclin1-mediated degradation of NIb-YFP. *N. benthamiana* plants were pre-treated with the relevant TRV vectors for 21 days and then agroinfiltrated with NIb-YFP and empty vector (Vec) or Myc-NbBeclin1. Total protein was extracted from infiltrated leaves at 3 dpi. Immunoblotting was performed using GFP or Myc antibody. CBB-stained Rubisco large subunit was used as a loading control. (**d**) Quantification of TuMV RNA levels by qRT-PCR in NbATG8a, NbATG8f, NbATG2, NbATG5 or NbATG7-silenced *N. benthamiana* plants when TuMV was co-expressed with empty vector (Vec) or NbBeclin1. *N. benthamiana* plants were pre-treated with the relevant TRV vectors for 8 days and then agroinfiltrated with TuMV with Vec or NbBeclin1 in the newly emerged leaves. Total RNA was extracted from infiltrated leaves at 60 hpi. Values were normalized against NbActin transcripts in the same sample, which showed means of fold change ± SD relative to the control plants (pre-treated with TRV1 and TRV2-GUS). The data were analyzed using Student's *t* test and asterisks denote significant differences when compared to TRV-GUS-treated plants. Double asterisks indicate significant differences (P < 0.01).

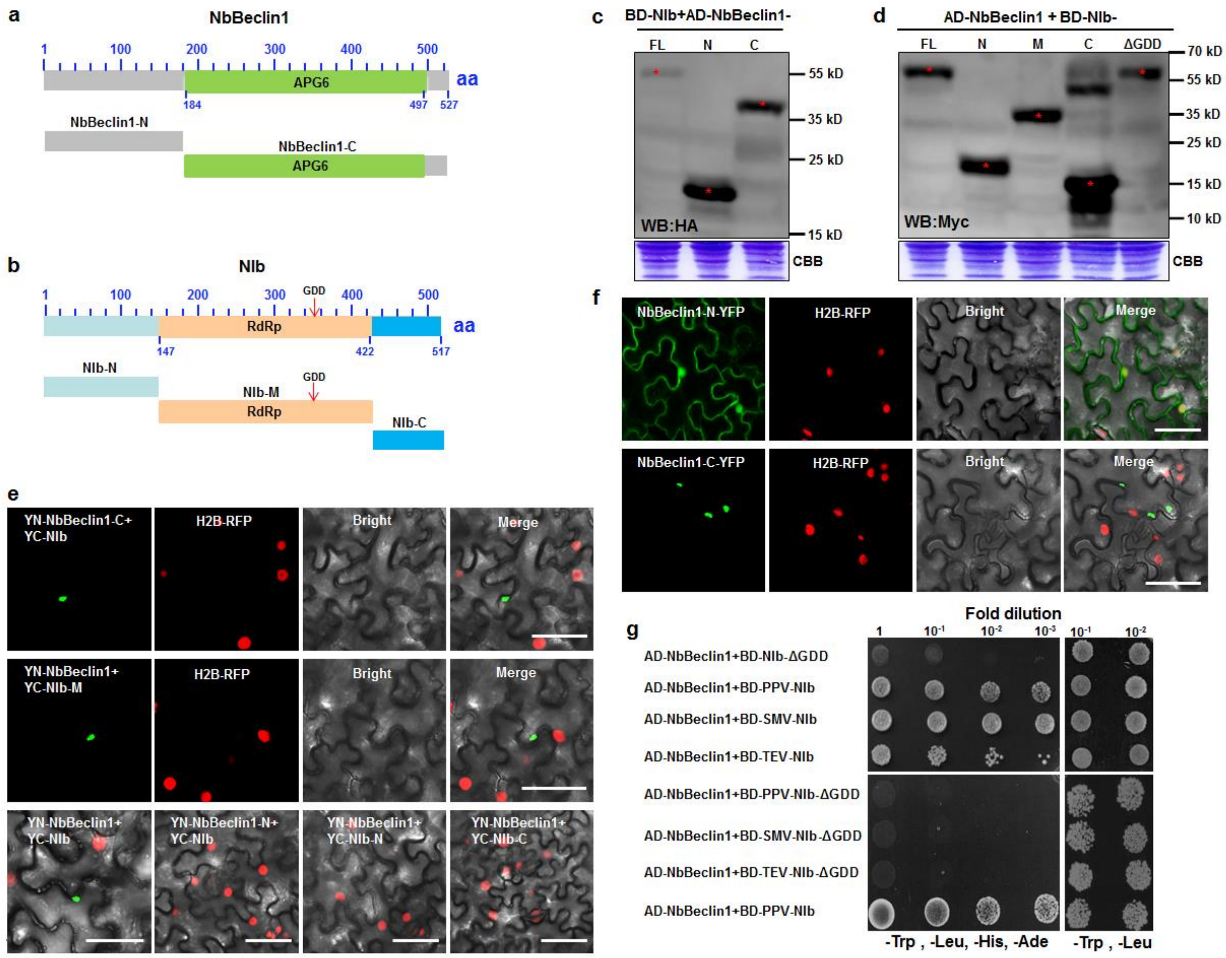

Supplementary Figure 8. Expression of NbBeclin1, NIb and their corresponding truncated mutants in yeast, BIFC analysis of NbBeclin1 and NIb interactions, subcellular localization of NbBeclin1-N and –C, and Y2H analysis of the possible interaction between NbBeclin1 and potyviral NIbs and between NbBeclin1 and the GDD mutants of potyviral NIbs. (a) Schematic representation of NbBeclin1 full-length (FL) and truncated proteins. The positions of the first and last amino acid (aa) residues are indicated. Based on the conserved domain prediction (http://www.ncbi.nlm.nih.gov), NbBeclin1 may be divided into two fragments: the N-terminal domain (183 aa) of unknown function (NbBeclin1-N) and the C-terminal APG6 domain (NbBeclin1-C). APG6, autophagy protein Apg6. **(b)** Schematic representation of NIb full-length (FL) and truncated proteins. NIb may be divided into three fragments: the N-terminal domain (NIb-N), the RdRp domain (NIb-M), and the C-terminal domain (NIb-C). RdRp, RNA-dependent RNA polymerase. The red arrow indicates the position of GDD. **(c,d)** Western blotting (WB) analysis of total protein extracts from yeast cells transformed with the indicated plasmids. The recombinant proteins derived from AD and BD vectors are HA- and Myc-tagged, respectively. Antibodies against HA- (@HA) and Myc (@Myc)-tags were applied. CBB-staining of the total proteins serves as a loading control. Red asterisks indicate that the detected proteins are of expected sizes. **(e)** BiFC assays on the interaction between NIb and NbBeclin1 truncated

proteins and between NbBeclin1 and NIb truncated proteins in H2B-RFP transgenic *N. benthamiana* leaves at 48 hpi. **(f)** Subcellular localization of NbBeclin1 truncated proteins in H2B-RFP transgenic *N. benthamiana* leaf cells at 48 hpi. Bars = 50 μm (**e-f**). **(g)** Y2H assays to detect possible interactions between NbBeclin1 and TuMV NIb GDD mutant (NIb-ΔGDD), and between NbBeclin1 and NIbs from PPV, SMV, and TEV, and between NbBeclin1 and corresponding NIb GDD mutants.

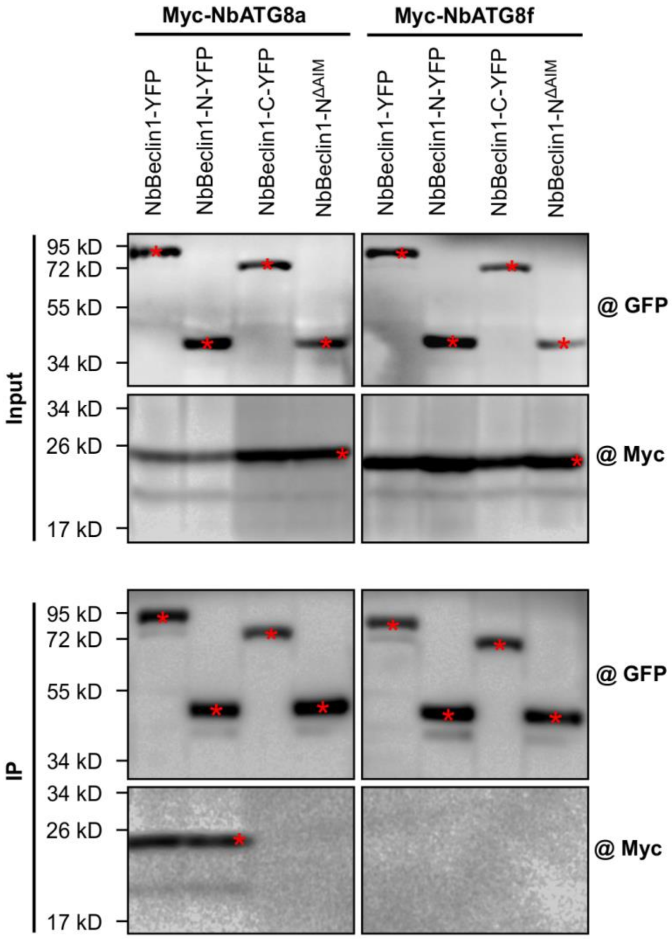

Supplementary Figure 9. Co-IP analysis of NbBeclin1 and NbATG8a, and NbBeclin1 and NbATG8f *in planta*. *N. benthamiana* leaves were infiltrated with *A. tumefaciens* cells harboring plasmids indicated. Leaf extracts were incubated with GFP-Trap®_MA magnetic agarose beads (ChromoTek). Samples before (Input) and after (IP) immunopurification were analyzed by immunoblotting using GFP or Myc antibody. Red asterisks indicate that the detected proteins are of expected sizes.

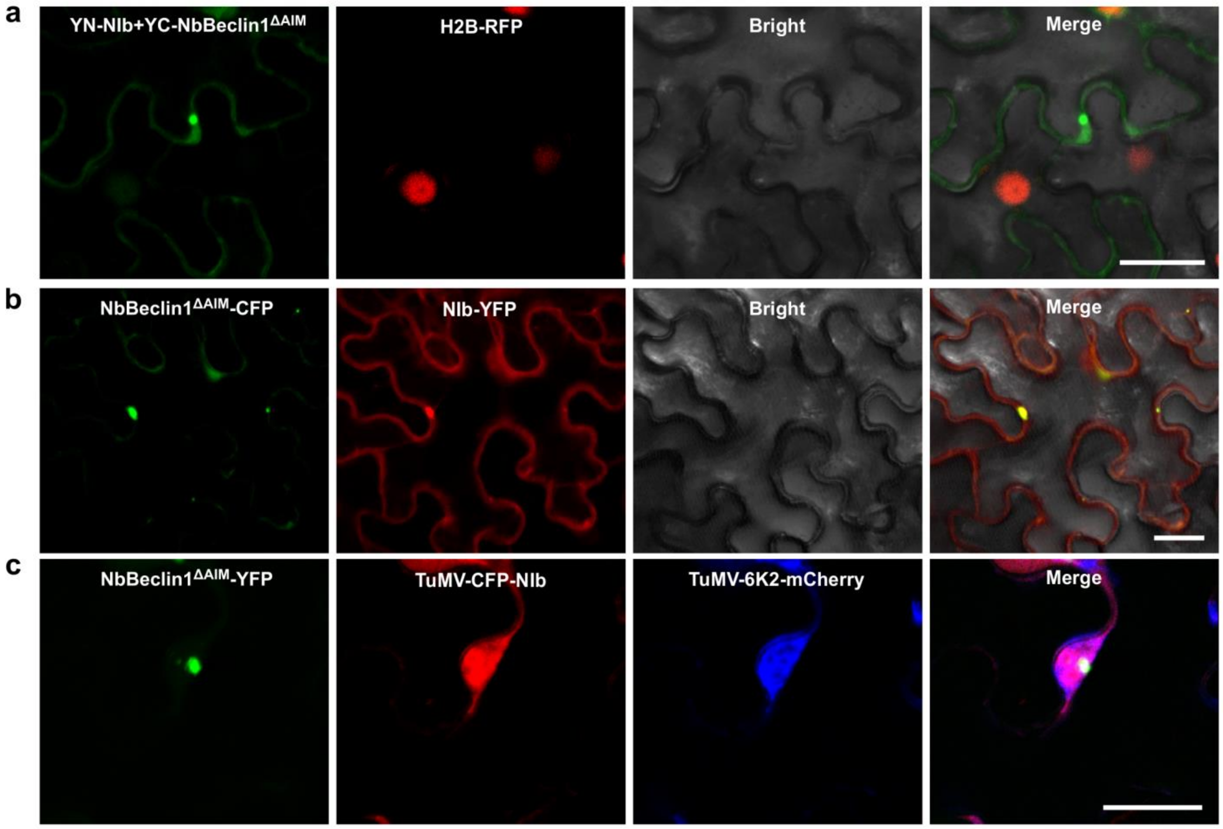

Supplementary Figure 10. The NbBeclin1 AIM is not required for the interaction with NIb and VRC. (**a**) BIFC assay of the NbBeclin1 AIM mutant (NbBeclin1$^{\Delta AIM}$) and NIb in H2B-RFP transgenic plants at 48 hpi. (**b**) Co-localization of NIb-YFP with NbBeclin1$^{\Delta AIM}$-CFP in *N. benthamiana* leaf cells by confocal microscopy at 48 hpi. (**c**) NbBeclin1$^{\Delta AIM}$-YFP was transiently expressed in leaf cells infected by TuMV-6K2-mCherry-CFP-NIb. NbBeclin1$^{\Delta AIM}$ -YFP co-localized with CFP-NIb and 6K2-mCherry-stained aggregations. Bars, 25 μm.

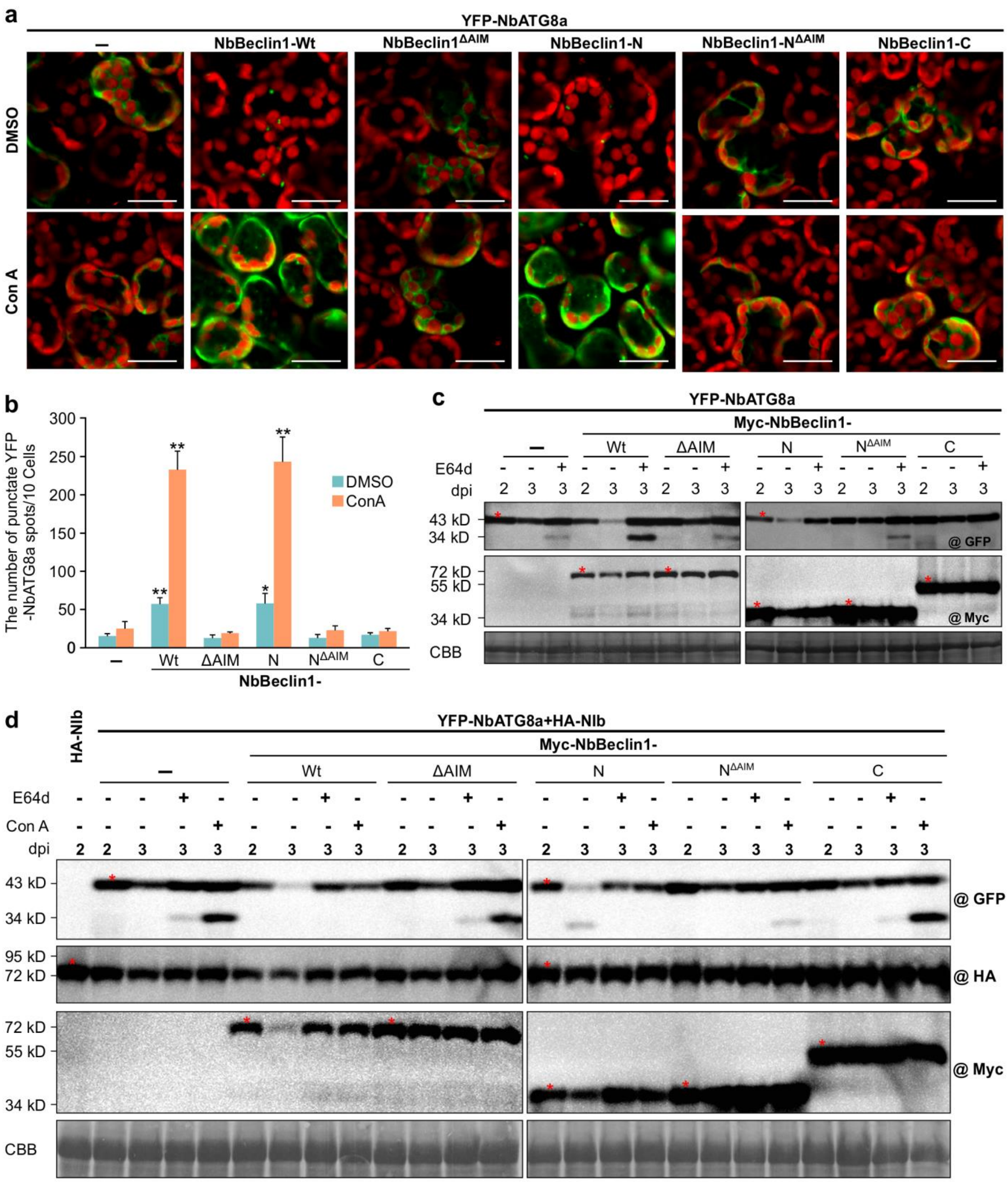

Supplementary Figure 11. The N-terminus of NbBeclin1 containing the AIM is required for the formation of autophagosomes and for NbATG8a degradation, but it is not sufficient for the degradation of NIb. (**a, b**) Concanamycin A (Con A) treatment increases the number of autophagosomes in wild type NbBeclin1(NbBeclin1-Wt) or NbBeclin1-N expressing samples. *N. benthamiana* leaves expressing YFP-NbATG8a alone (-), or co-expressing YFP-NbATG8a with

NbBeclin1-Wt, NbBeclin1$^{\Delta AIM}$, NbBeclin1-N, NbBeclin1-N$^{\Delta AIM}$ or NbBeclin1-C. Infiltrated leaves were treated with Con A or DMSO after 36 hpi, and the number of autophagosomes was counted at 12 h after treatment. Infiltration experiments were repeated three times and 60 cells in total were counted for punctate spots. Values represent the mean spots ± SD. Asterisks indicate significant differences (Student's *t* test, two-sided, $**P < 0.01$, $*P < 0.05$). (**c**) E64D treatment suppressed autophagy-mediated YFP-NbATG8a degradation in NbBeclin1or NbBeclin1-N expressing samples. Infiltrated leaves were harvested from the *N. benthamiana* leaves agroinfiltrated with plasmids indicated at 2 dpi, 3 dpi, or 3 dpi with E64d treatment (E64d was treated onto the infiltrated leaves in advance 12 h). E64d treatment obviously increased YFP-NbATG8a protein levels when YFP-NbATG8a was co-expressed with NbBeclin1-Wt or NbBeclin1-N at 3 dpi, suggesting that the expression of NbBeclin1-Wt or NbBeclin1-N stimulates autophagy. The membranes were probed with GFP (the upper row), or Myc antibodies (the middle row). CBB-staining of Rubisco large subunit serves as a loading control. (**d**) E64D or Con A treatment blocks autophagy-mediated the degradation of YFP-NbATG8a and NIb in NbBeclin1-Wt expressing samples. Immunoblotting of total protein extracts from the *N. benthamiana* leaves agroinfiltrated with plasmids indicated at 2 dpi, 3 dpi, 3 dpi with E64d treatment, or 3 dpi with Con A treatment (E64d or Con A was treated to the infiltrated leaves in advance 12 h). The membranes were probed with GFP, HA or Myc antibodies. CBB-staining of Rubisco large subunit serves as a loading control. Red asterisks (**c**, **d**) indicate that the detected proteins are of expected sizes.

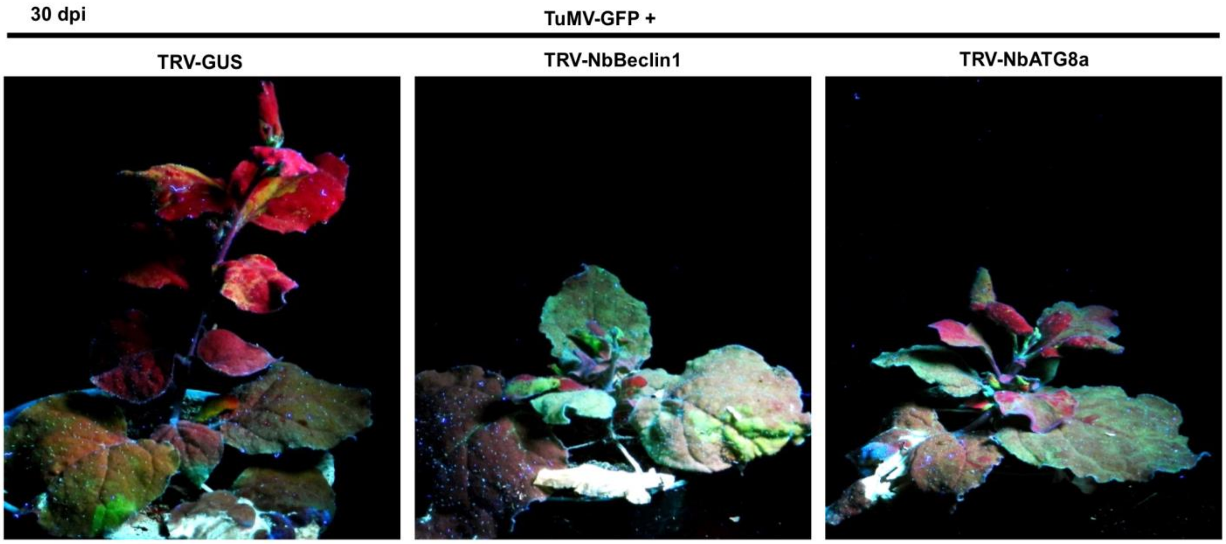

Supplementary Figure 12. GFP fluorescence in leaves of TuMV-GFP infected plants pre-treated with TRV1 together with TRV-GUS, TRV-NbBeclin1, or TRV-NbATG8a. Plants were photographed under UV light at 30 dpi with TuMV-GFP.

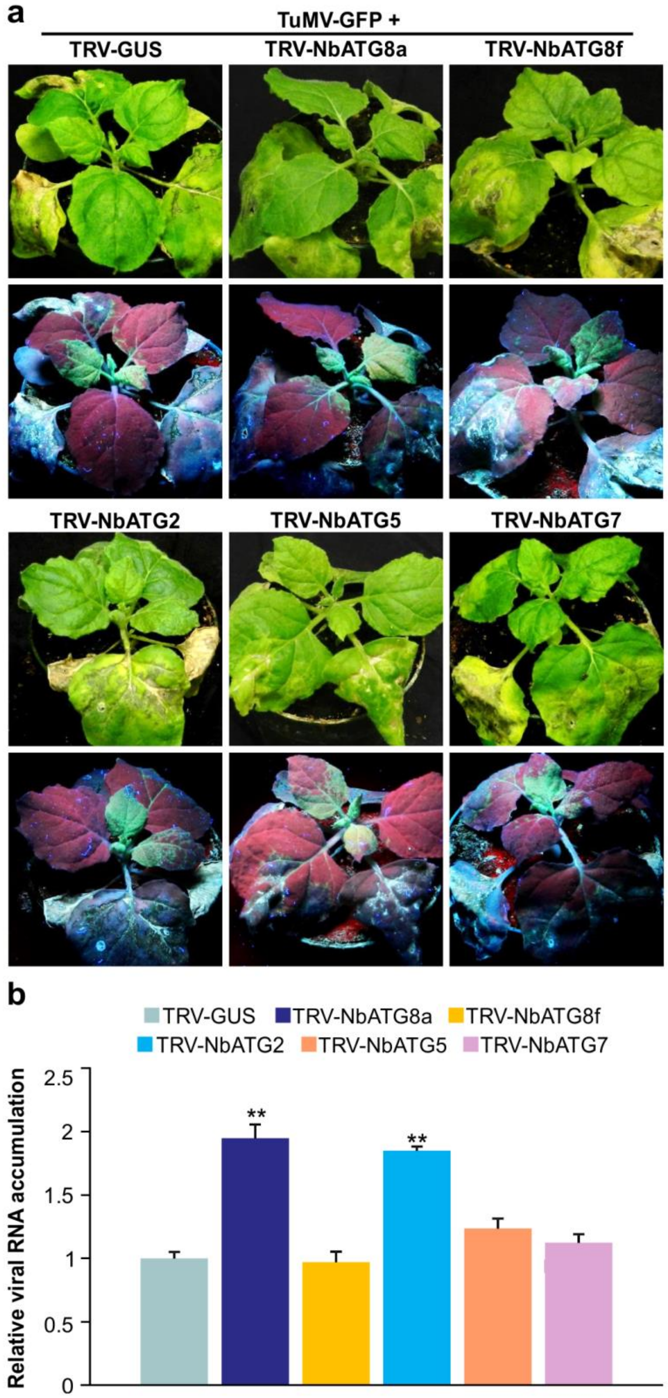

Supplementary Figure 13. Knock-down of *NbATG8f, NbATG5 or NbATG7* does not significantly affect TuMV infection. (a) TuMV symptoms and GFP fluorescence in plants pre-inoculated with TRV1 together with TRV2-GUS (control), TRV2-NbATG8a, TRV2-NbATG8f, TRV2-NbATG2, TRV2-NbATG5 or TRV2-NbATG7 for 7 days and then infected by TuMV-GFP. Plants were photographed under regular light or UV at 6 dpi. (**b**) Relative genomic RNA levels in the above plants. RNA was extracted from TuMV-GFP-infected leaves at 6 dpi. The values are presented as means ± SD (n=3) relative to the control plants (pre-treated with TRV1

and TRV2-GUS) and were normalized against *NbActin* transcripts. Data were analyzed using Student's *t* test and asterisks denote significant differences compared to the TuMV RNA level in the TRV-GUS-treated plants (** $P < 0.01$).

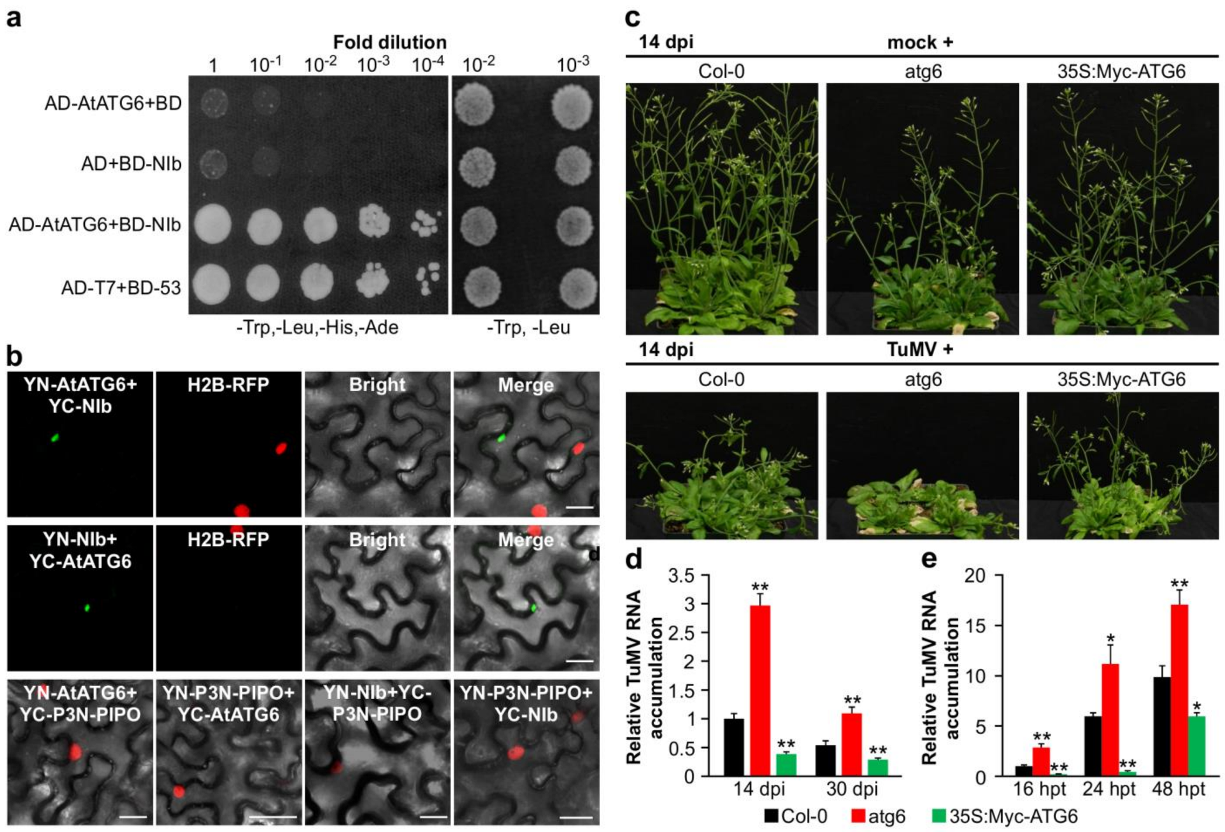

Supplementary Figure 14. AtATG6 inhibits TuMV infection. (**a**) Y2H assays. AtATG6 interacted with NIb. (**b**) Confirmation of the interaction of NIb and AtATG6 by BiFC in H2B-RFP transgenic *N. benthamiana* leaves at 48 hpi. Yellow fluorescence (green) was observed as a consequence of the complementation of the YN and YC tagged with AtATG6 and NIb. Nuclei of tobacco leaf epidermal cells are indicated by expression of the H2B-RFP transgene (red). Bars, 50 μm. (**c**) Symptoms of TuMV-infected wild type (Col-0), *atg6* mutant and 35S:AtATG6 Arabidopsis plants. Images were taken at 14 dpi. Mock, inoculated with buffer; TuMV, inoculated with TuMV. (**d**) Quantification of TuMV RNA levels by qRT-PCR. RNA was extracted from TuMV systemically infected leaves at 14 and 30 dpi. The values are shown as means ± SD relative to Col-0 and *NbActin* were used as the internal reference. Three independent experiments, each consisting of three biological replicates, were carried out. Values from one representative result were used to plot a histogram. The data were analyzed using Student's *t* test and double asterisks denote significant differences compared to the TuMV-infected *atg6 or* 35S:AtATG6 Arabidopsis plants from Col-0 plants infected by TuMV (two-sided, $**P < 0.01$). (**e**) Quantification of TuMV RNA levels by qRT-PCR. RNA was extracted from wild type Col-0, atg6 and 35S:Myc-AtATG6 Arabidopsis protoplasts transfected with TuMV-GFP at 16, 24 and 48 hpt. Arabidopsis protoplast transformation experiments were repeated independently at least three times, and results from one representative experiment was shown. Values represent the mean ± SD relative to Col-0 and were normalized with *AtActin II* as the internal reference. The data were analyzed using Student's *t* test and asterisks denote significant differences to the TuMV RNA level in wild type (Col-0) plants (two-sided, $*P < 0.05$, $**P < 0.01$).

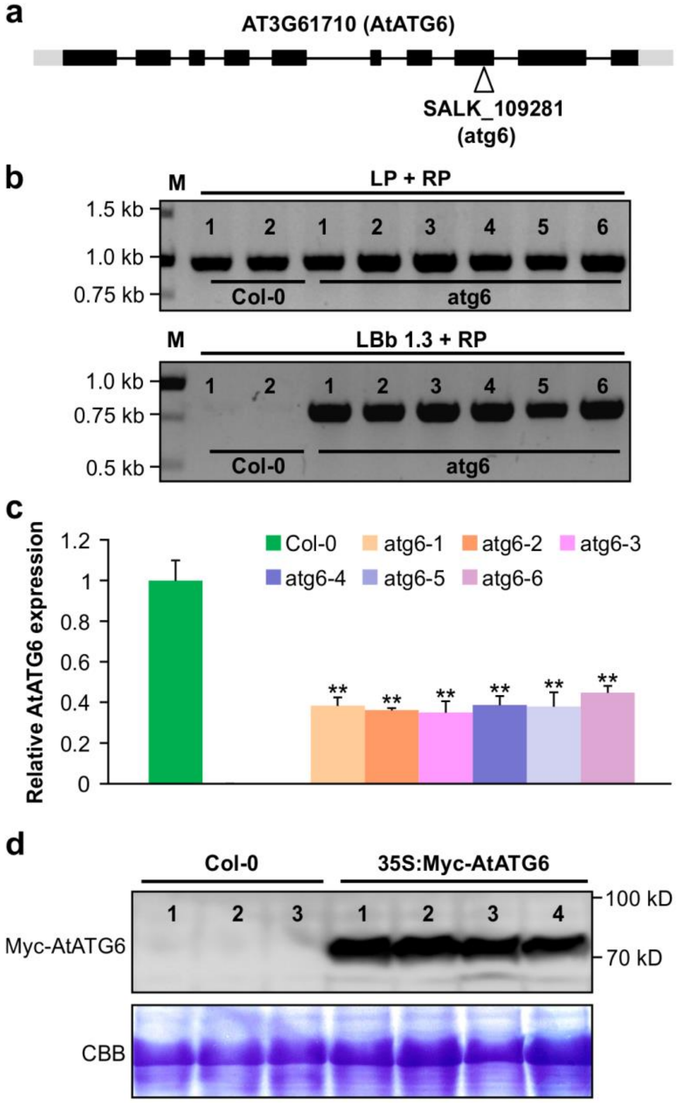

Supplementary Figure 15. Confirmation of *atg6* T-DNA insertion and *ATG6* overexpression lines. (**a**) Schematic diagram of the *AtATG6* gene and T-DNA insertion sites (triangles) in *Arabidopsis* T-DNA insertion mutants. Exons and introns are indicated by dark boxes and lines, respectively. Gray boxes represent 5' and 3' untranslated regions. (**b**) Screening for heterozygote *atg6* T-DNA insertion lines. PCR was conducted using genomic DNA from wild type (Col-0) and *atg6* (SALK_109281) Arabidopsis plants. Two gene-specific primers (LP+RP) were used to detect wild type and heterozygous *atg6* genotype. A T-DNA specific primer and a gene- specific primer (LBb 1.3+RP) were used to amplify a single PCR fragment from heterozygous *atg6* genotype. The results are consistent with the pattern of heterozygous genotype for all T-DNA lines. LP, left genomic primer; RP, right genomic primer; LBb 1.3, Left border primer of the T-DNA insertion. (**c**) qRT-PCR analysis of *AtATG6* expression in Col-0 and heterozygous *atg6* Arabidopsis plants. qRT-PCR was performed using cDNA derived from leaf tissues of Col-0 and *atg6* Arabidopsis plants with *AtATG6* specific primers. The expression level of *AtActin II* was used as an internal control. Values represent means relative to *AtATG6*

transcripts in Col-0 Arabidopsis plants. Double asterisks indicate highly significant differences (two-sided, $^{**}P < 0.01$) between WT and *atg6* Arabidopsis plants (Student's *t* test). (**d**) Immunoblotting detection of the Myc-AtATG6 protein in 35S:Myc-AtATG6 transgenic Arabidopsis plants. CBB-stained Rubisco large subunit was used as a loading control.

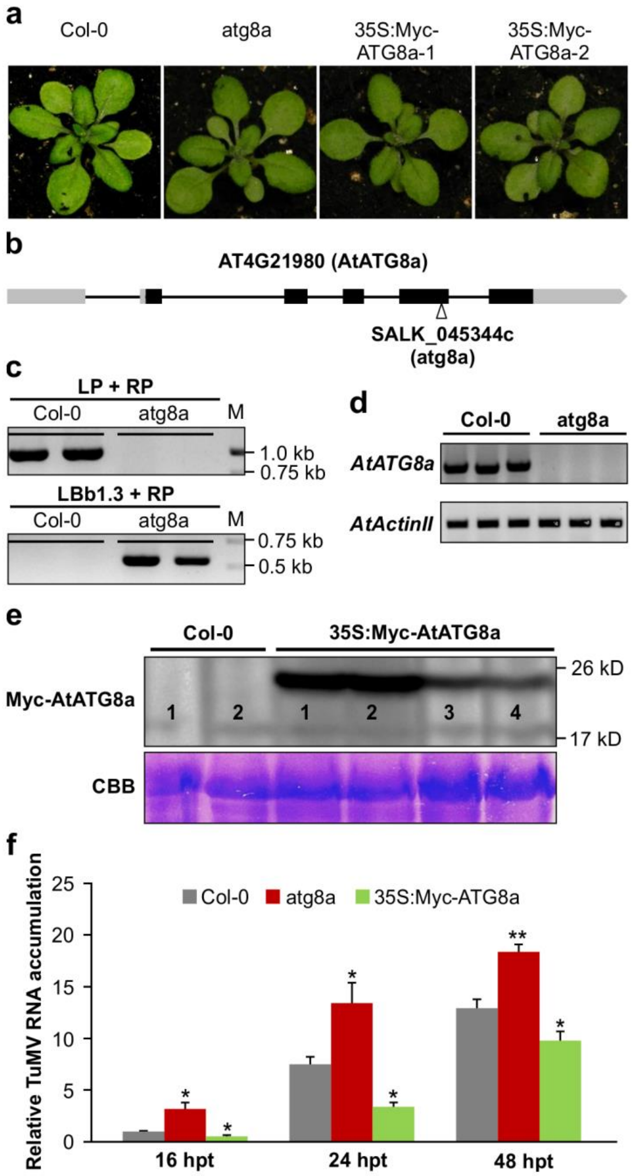

Supplementary Figure 16. AtATG8a suppresses TuMV replication. (**a**) Phenotypes of Col-0, *atg8a* mutant, and *AtATG8a* overexpression (35S:Myc-ATG8-1 and 35S:Myc-ATG8-2) Arabidopsis plants. Images were taken 20 days after seeding. (**b**) Schematic diagram of the *AtATG8a* gene and T-DNA insertion sites (triangles) in Arabidopsis T-DNA insertion mutants. Exons and introns are indicated by dark boxes and lines, respectively. Gray boxes represent 5' and 3' untranslated regions. (**c**) Confirmation of homozygous *atg8a* T-DNA insertion lines. PCR was conducted using genomic DNA from Col-0 and *atg8a* (SALK_045344c) Arabidopsis plants. Two gene-specific primers (LP+RP) were used to detect wild type genotype. A T-DNA specific primer and a gene-specific primer (LBb 1.3+RP) were used to detect the T-DNA insertion. LP, left genomic primer; RP, right genomic primer; LBb 1.3, Left border primer of the T-DNA insertion. (**d**) RT-PCR analysis of *AtATG8* expression in Col-0 and *atg8* Arabidopsis plants. *AtActin II* gene was used as an internal control. (**e**) Immunoblotting detection of the Myc-AtATG8a protein in T1 35S:Myc-AtATG8a transgenic Arabidopsis plants. CBB-stained Rubisco

large subunit was used as a loading control. (**f**) qRT-PCR analyzed TuMV RNA levels. RNA was extracted from Col-0, *atg8a* and 35S:Myc-AtATG8a Arabidopsis protoplasts transfected with TuMV-GFP at 16, 24 and 48 hpt. Values represent the mean ± SD relative to Col-0 and were normalized with *AtActin II* as the internal reference (n=3). The data were analyzed using Student's *t* test and asterisks denote significant differences compared to the TuMV RNA level in Col-0 plants (two-sided, *$P < 0.05$, ** $P < 0.01$).

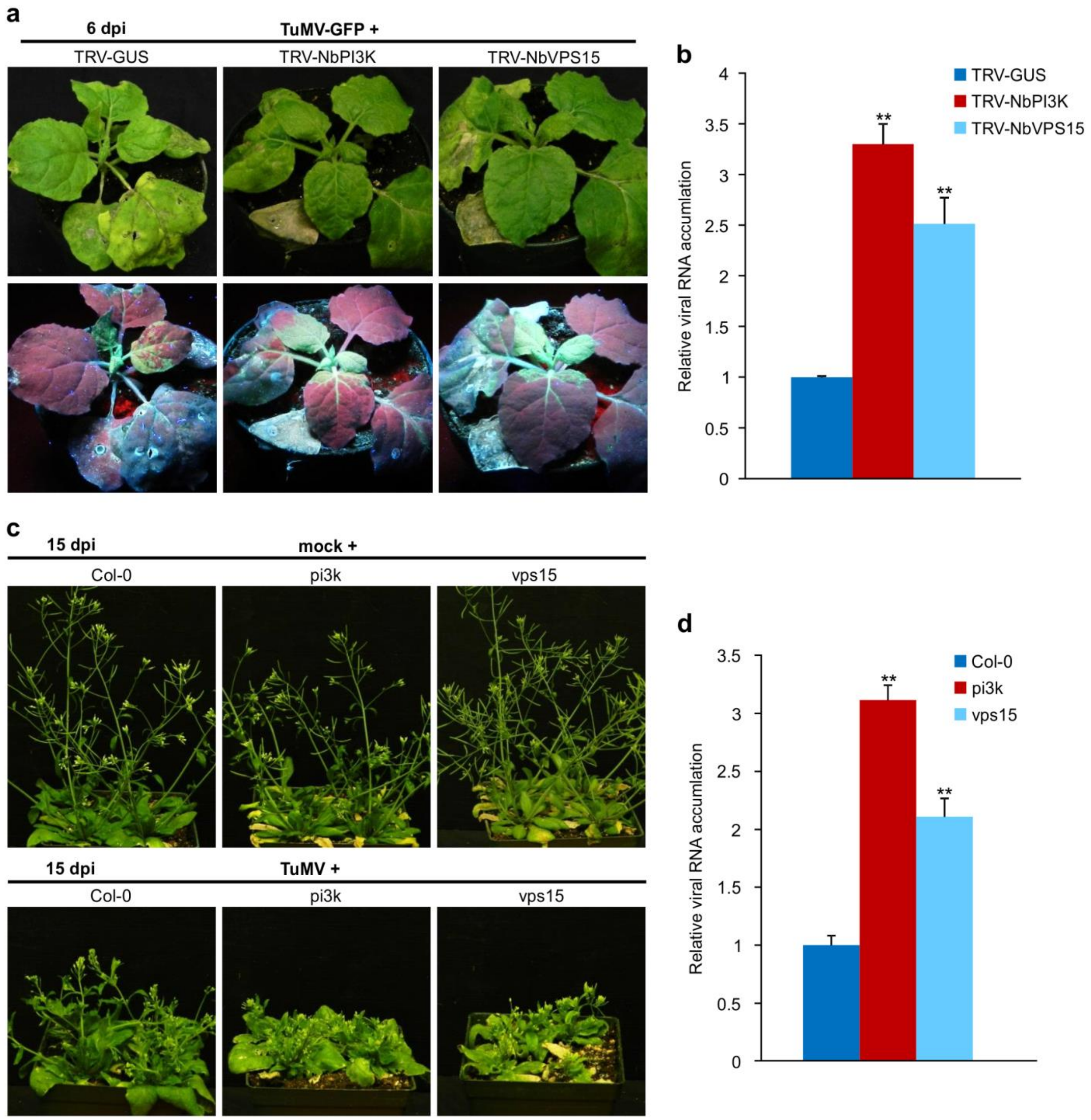

Supplementary Figure 17. Silencing of *PI3K* or *VPS15* enhances TuMV RNA accumulation. (**a**) Viral symptoms and GFP fluorescence in plants pre-inoculated with TRV1 together with TRV2-GUS (control), TRV2-NbPI3K or TRV2-NbVPS15 for 7 days and then infected by TuMV-GFP. Plants were photographed under regular light and UV light at 6 dpi. (**b**) Quantification of TuMV genomic RNA. RNA was extracted from TuMV-GFP systemically infected leaves at 20 dpi. The values are presented means ± SD relative to the control plants (TRV-GUS).Three independent experiments, each consisting of three biological replicates, were performed. Values from one representative result were used to plot a histogram and were normalized against *NbActin* transcripts. The data were analyzed using Student's *t* test (two-sided, * $P < 0.05$, ** $P < 0.01$). (**c**) Symptoms of TuMV-infected wild type (Col-0), *pi3k* and *vps15*

mutant Arabidopsis plants. Images were taken at 15 dpi. Mock, inoculated with buffer; TuMV, inoculated with TuMV. (**d**) Quantification of TuMV RNA levels by qRT-PCR. RNA was extracted from TuMV systemically infected leaves at 20 dpi. The values are shown means ± SD relative to the Col-0. Error bars represent SD (n=3). The data were analyzed using Student's *t* test and double asterisks denote highly significant differences compared to the TuMV RNA level in Col-0 plants (two-sided, $^{**}P < 0.01$).

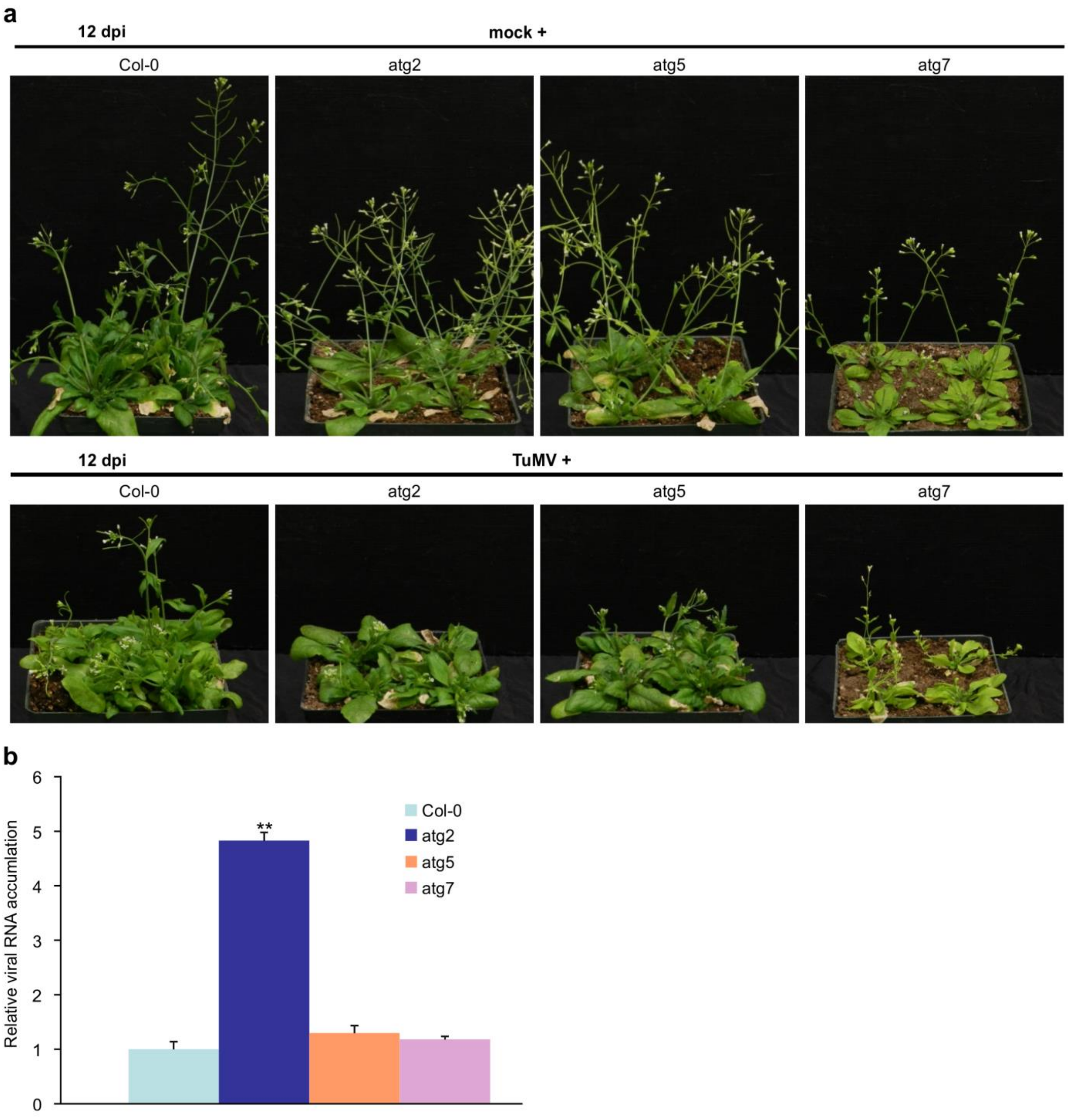

Supplementary Figure 18. Knock-out of *ATG5 or ATG7* does not significantly affect TuMV RNA accumulation. (a) Symptoms of TuMV-infected wild type (Col-0), *atg2*, *atg5,* and *atg7* mutant Arabidopsis plants. Photos were taken at 12 dpi. Mock, inoculated with buffer; TuMV, inoculated with TuMV. (**b**) Quantification of TuMV RNA levels by qRT-PCR. RNA was extracted from TuMV systemically infected leaves at 12 dpi. The values are shown as means ± SD (n=3) relative to Col-0 and were normalized against *AtActin II* transcripts. The data were analyzed using Student's *t* test and double asterisks denote a highly significant difference compared to the TuMV RNA level in Col-0 plants (two-sided, ** $P < 0.01$).

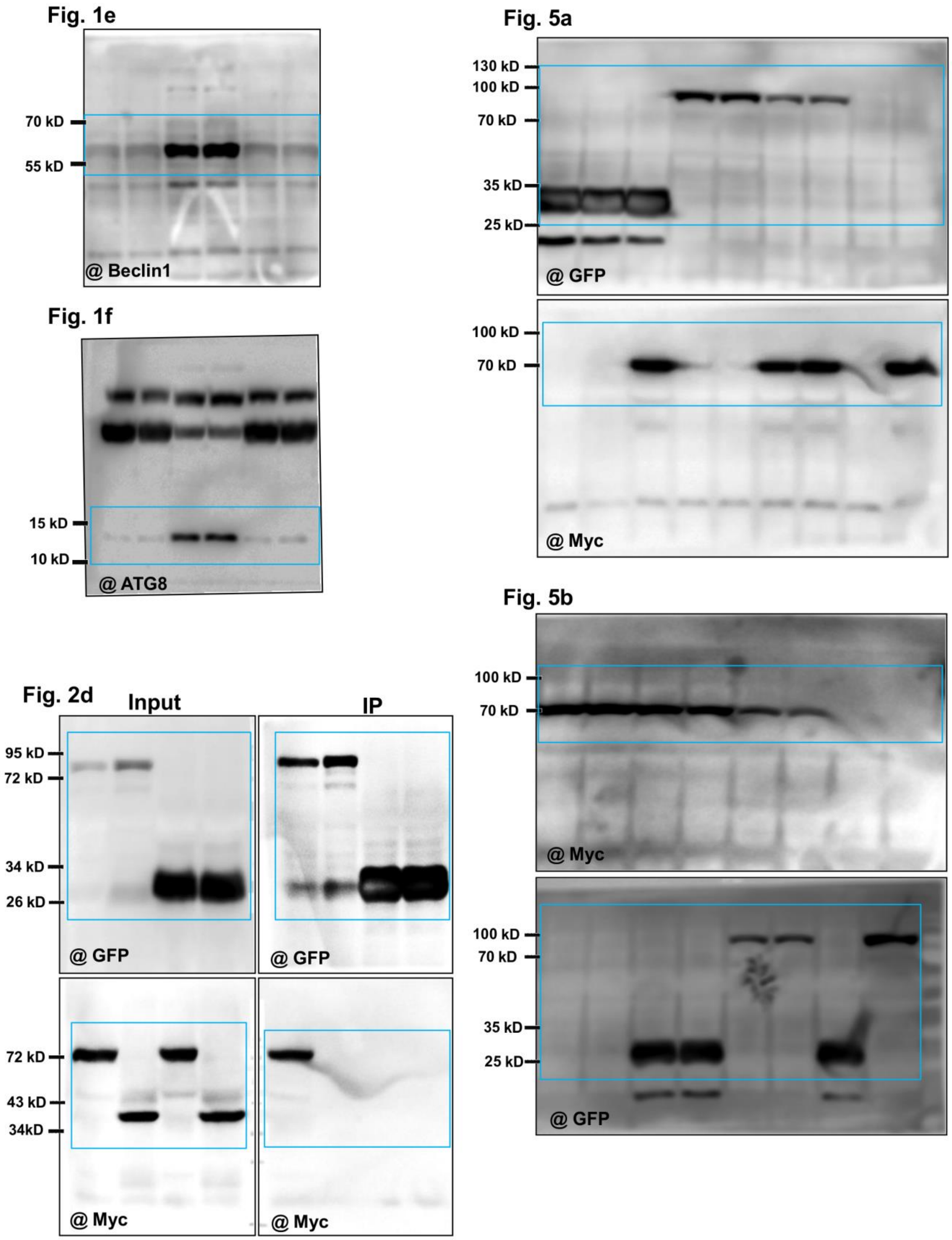

Supplementary Figure 19. Original immunoblot images shown in Figs. 1e, 1f, 2d, 5a and 5b. The molecular weight markers and the antibodies used are indicated on the images.

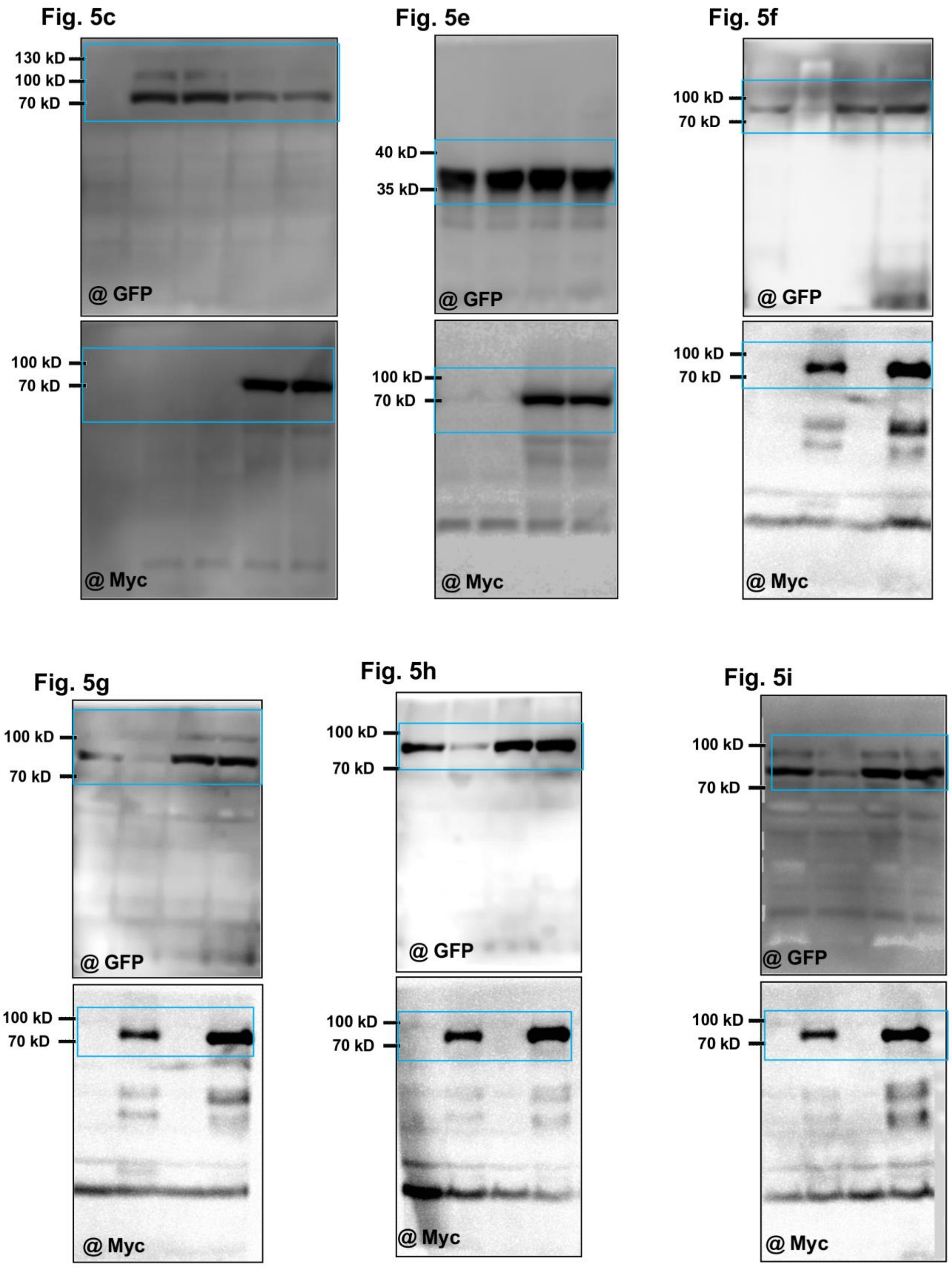

Supplementary Figure 20. Original images for immunoblots shown in Fig. 5c, e, f, g, h, i. The molecular weight markers and the antibodies used are indicated on the images

Fig. 7b

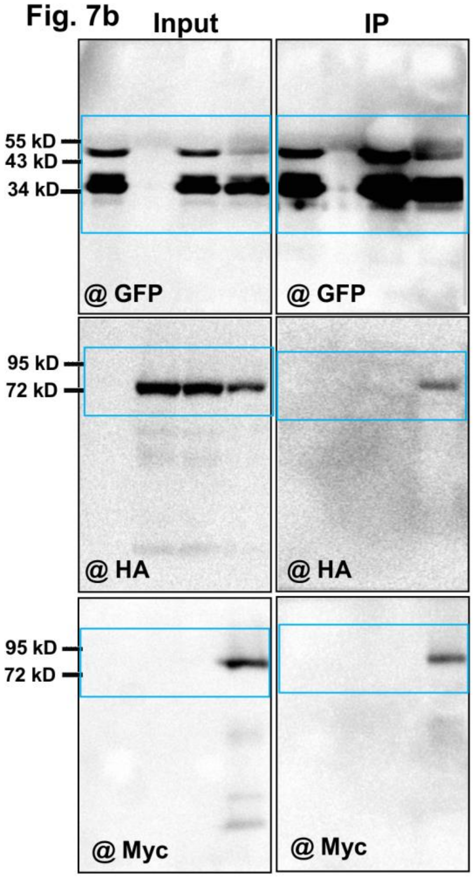

Fig. 7c

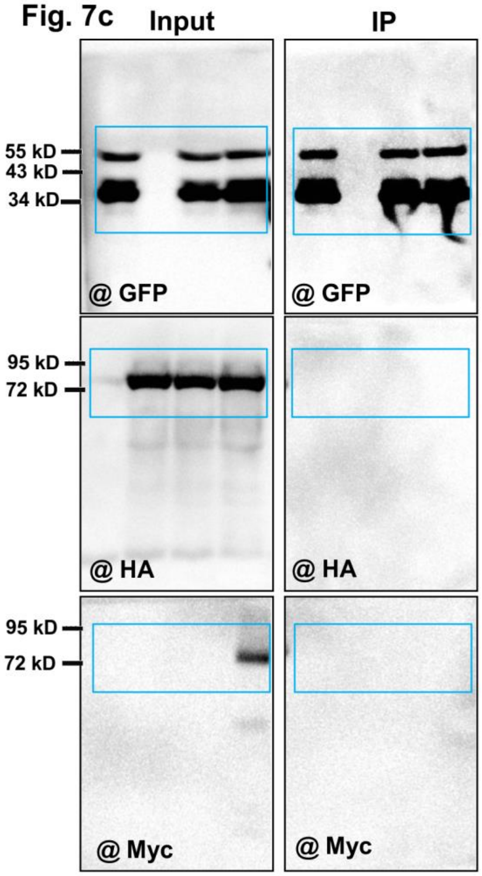

Fig. 7d

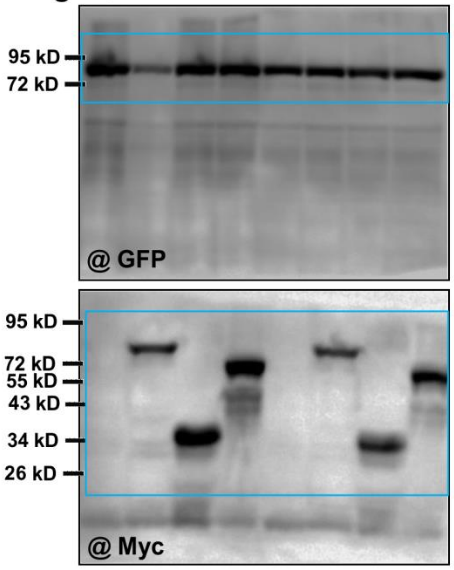

Fig. 7g

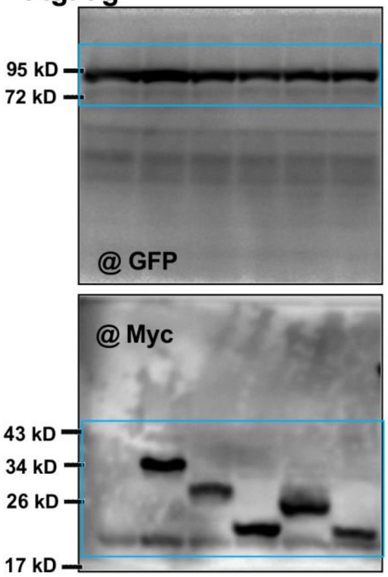

Supplementary Figure 21. Original images for immunoblots shown in Figs. 7b, c, d, g. The molecular weight markers and the antibodies used are indicated on the images.

Fig. 9d

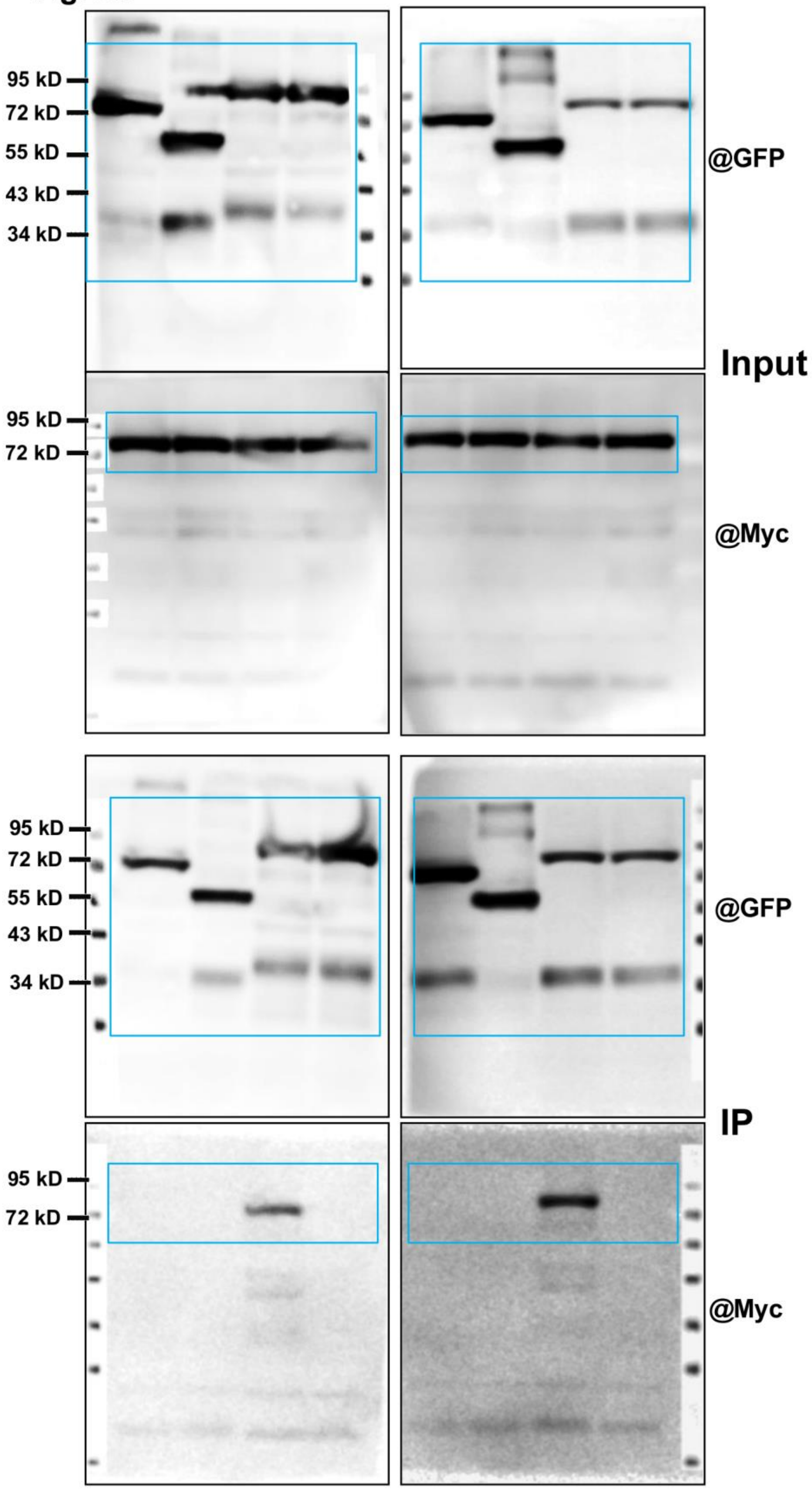

Supplementary Figure 22. Original images for Fig. 9d. The molecular weight markers and the antibodies used are indicated on the images.

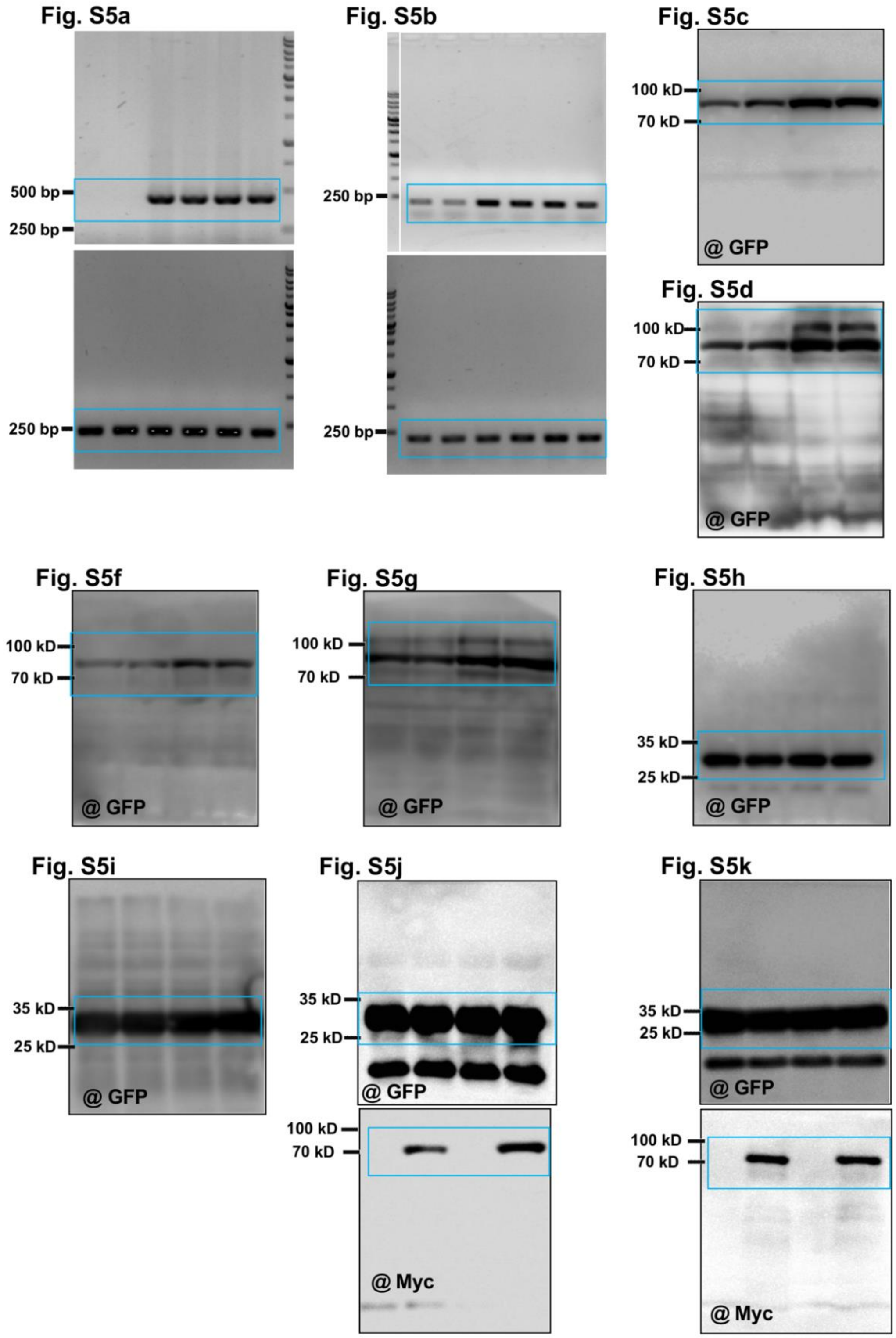

Supplementary Figure 23. Original images for Supplementary Figs. 5a through 5d, and 5f through 5k. The molecular weight markers and the antibodies used are indicated on the images**.**

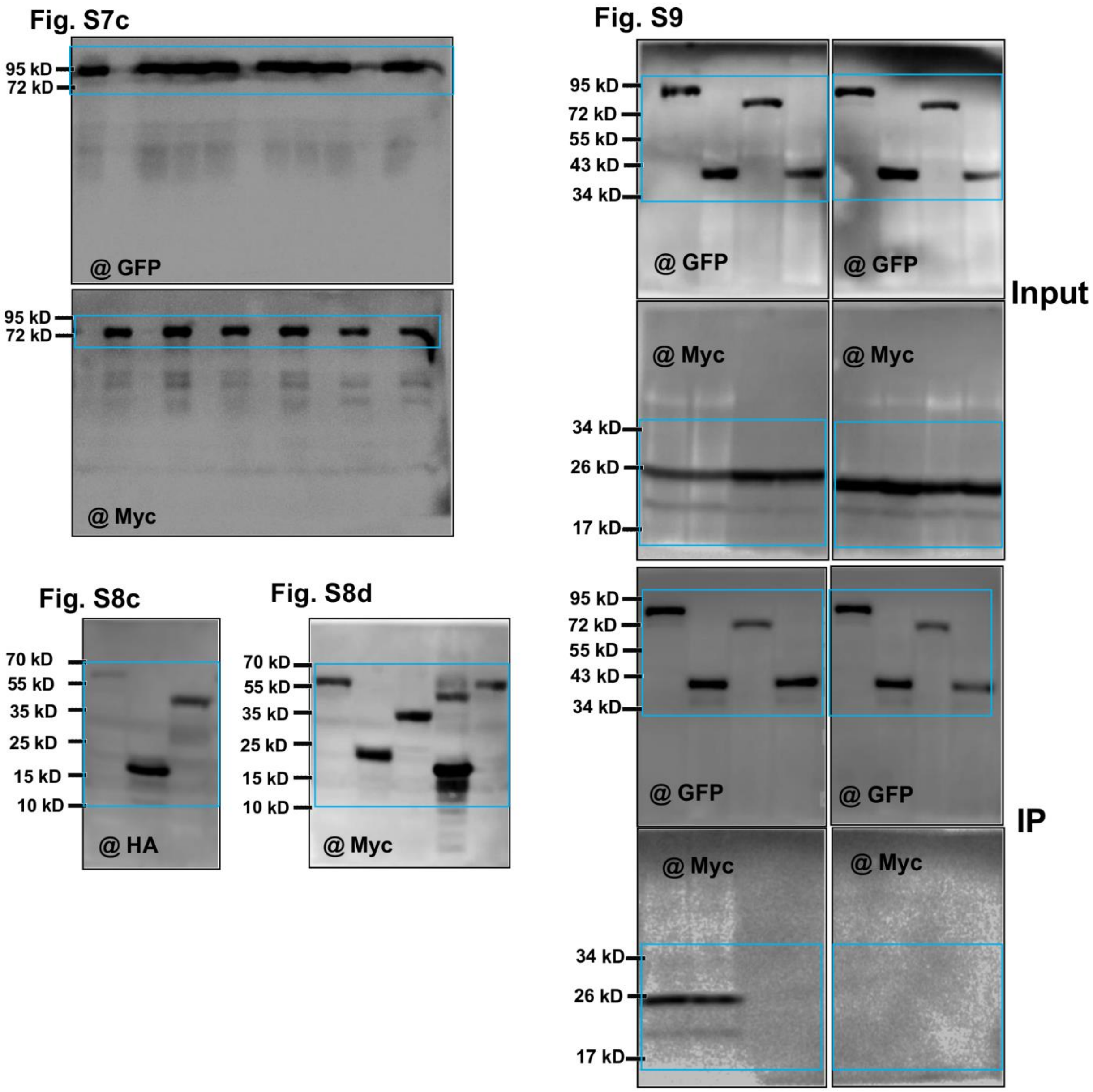

Supplementary Figure 24. Original images for immunoblots shown in Supplementary Figs. 7c, 8c, 8d, and 9. The molecular weight markers and the antibodies used are indicated on the images.

Fig. S11c

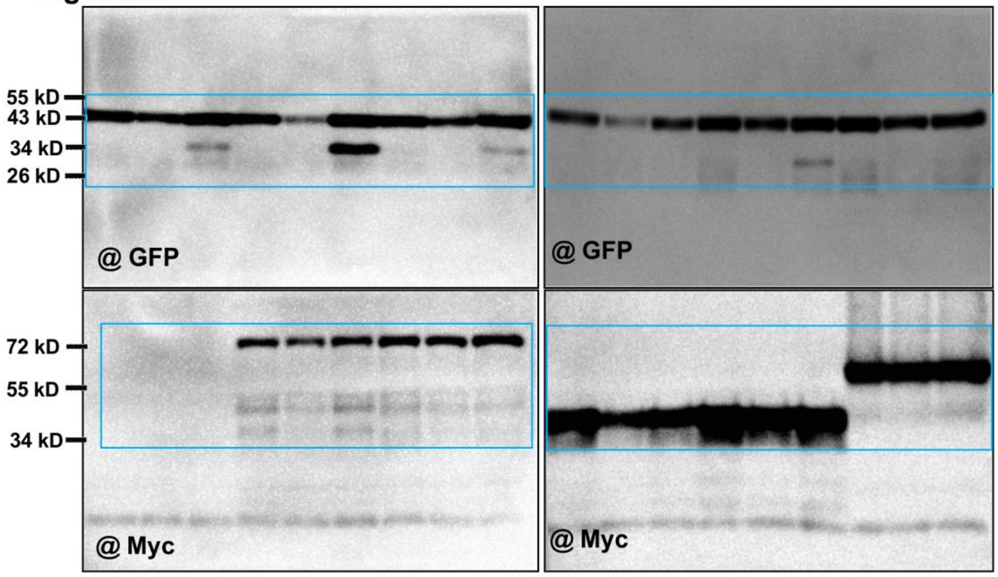

Fig. S11d

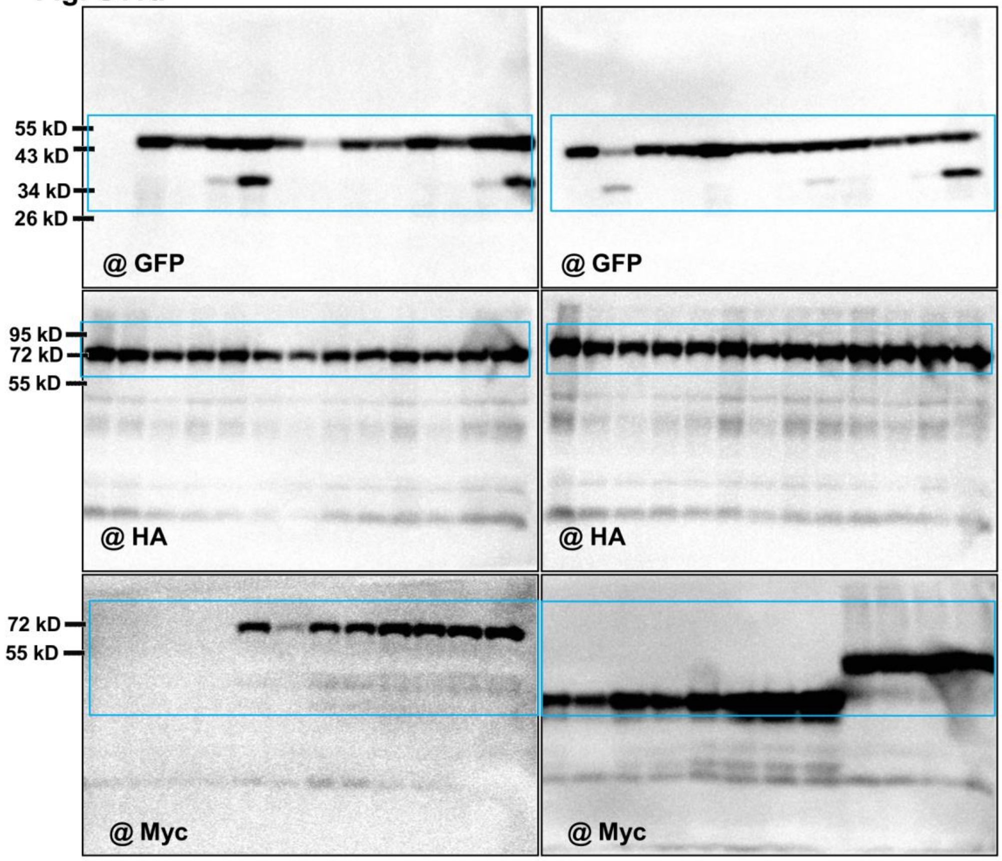

Supplementary Figure 25. Original images for for immunoblots shown in Supplementary Figs. 11c and 11d. The molecular weight markers and the antibodies used are indicated on the images.

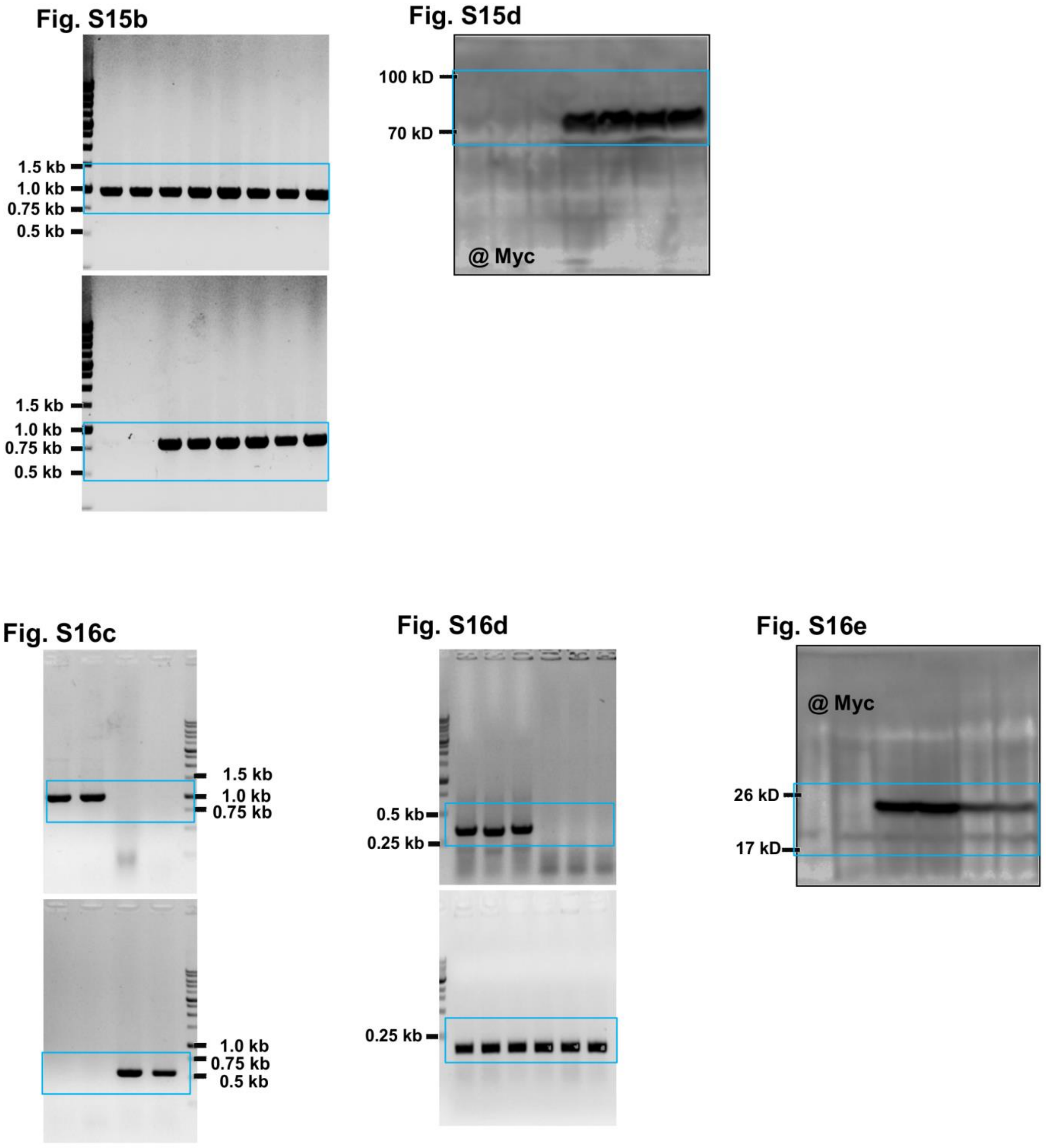

Supplementary Figure 26. Original images for Supplementary Figs. 15b, 15d, 16c, 16d and 16e. The molecular weight markers and the antibodies used are indicated on these images.

Supplementary Table 1: Primers used in this study

**Prime name**	**Sequence (5’ – 3’)**
TuMV-CP-qPCR-F	TGGCTGATTACGAACTGACG
TuMV-CP-qPCR-R	CTGCCTAAATGTGGGTTTGG
AtACTIN2-qPCR-F	CACCACAACAGCAGAGCGGGA
AtACTIN2-qPCR-R	TCCCACAAACGAGGGCTGGA
NbActin-qPCR-F	AAAGACCAGCTCATCCGTGGAGAA
NbActin-qPCR-R	TGTGGTTTCATGAATGCCAGCAGC
NbBeclin1-qPCR-F	GACCTGCGTAAAGGAGTTTGCTGAC
NbBeclin1-qPCR-R	CCAACAAACCAGTAGAGCACCCAC
NbPI3K-qPCR-F	AGCTGTGCTGGTTACTCCGTCATC
NbPI3K-qPCR-R	GTACTGACTTTCTGCTCCACCCATA
NbVPS15-qPCR-F	ACCTGCCTTGATCATCCACATGTTTGG
NbVPS15-qPCR-R	CACCGTGACATACTCCATGTTCATGAC
NbATG2-qPCR-F	GATGAAAACAAAAATGTTAAGGGTCGC
NbATG2-qPCR-R	TCTAGATACCCGAGTTCCACAATCAGG
NbATG9-qPCR-F	TGCATATAGTCAGCGTACATGAATGATG
NbATG9-qPCR-R	GCAATTGGCTCAACATTCAAGCTCTCACC
NbATG3-qPCR-F	GAATACTATCCAGTCAATCTCCTCATAC
NbATG3-qPCR-R	TCCAGTGAGCCACACACGAGGGGTTTG
NbATG5-qPCR-F	GAAGCTTATCTCCGAATCTCGTCTAAGC
NbATG5-qPCR-R	CCAACTTTCAACTGCAGGTGCATCTTG
NbATG7-qPCR-F	AGGTCTCGATGTCTAATCCTCTACGCCAG
NbATG7-qPCR-R	AATCAAATCAGACAAATGTCTGCAATCCTG
NbATG8a-qPCR-F	CACCCACTTGAAAGGCGACAGGC
NbATG8a-qPCR-R	GCCTTCTCAGCACTAAGCTTTATTCTC
AtATG6-qPCR-F	GATCGAAAAGGACAAAGTATTGGGGTATTC
AtATG6-qPCR-R	GAACCAGCCGCTGATATATTAGAAGGC
NbBeclin1-gw-F	GGGGACAAGTTTGTACAAAAAAGCAGGCTTCATGACGAAAAATAGCAGCAGTAG
NbBeclin1-gw-R	GGGGACCACTTTGTACAAGAAAGCTGGGTCAGATTGAAACTTGGTATTAGTTG
AtATG6-gw-F	GGGGACAAGTTTGTACAAAAAAGCAGGCTTCATGAGGAAAGAGGAGATTCC
AtATG6-gw-R	GGGGACCACTTTGTACAAGAAAGCTGGGTCAGTTTTTTTACATGAAGGCTTACTAG
NbATG8a-gw-F	GGGGACAAGTTTGTACAAAAAAGCAGGCTTCATGGCCAAAAGCTCCTTCAAATTG
NbATG8a-gw-R	GGGGACCACTTTGTACAAGAAAGCTGGGTCGAACGATCCGAATGTATTCTCTCC
NbATG8f-gw-F	GGGGACAAGTTTGTACAAAAAAGCAGGCTTCATGGCAAAGAGTTCATTCAAG
NbATG8f-gw-R	GGGGACCACTTTGTACAAGAAAGCTGGGTCCACCAAGTTAAAGTCCCCAAATG
NbBeclin1-C-gw-F	GGGGACAAGTTTGTACAAAAAAGCAGGCTTCATGCCGTTGTGTCTTGAATGCATG
NbBeclin1-N-gw-R	GGGGACCACTTTGTACAAGAAAGCTGGGTCTTGCTCAATCTGGGTCTGCG
PPV-NIb-gw-F	GGGGACAAGTTTGTACAAAAAAGCAGGCTTCATGTCCAAAACTACACATTGGCTC
PPV-NIb-gw-R	GGGGACCACTTTGTACAAGAAAGCTGGGTCTTGGTGCACAACAACGTTGGAC
NbBeclin1-N-AIM-F	GCTGTCGTTGCCCCTCCACCAGCTGCTTCA
NbBeclin1-N-AIM-R	TGGAGGGGCAACGACAGCTGATTCTTCCATAGCCTT
TRV2-NbBeclin1-BamH-F	CGGGATCCGCCTTCTTCTTCATACAATGGCTC
TRV2-NbBeclin1-Xho1-R	CCGCTCGAGGAGCTTTGGTCCAACTTTCCTG
TRV2-NbATG8a-BamH-F	CGGGATCCCTCCTTCAAATTGGAACACCCAC
TRV2-NbATG8a-Xho1-R	CCGCTCGAGCATCATGGCAGCTGTGGGAG
TRV2-NbATG8f-BamH-F	CGGGATCCTACCCCGATAGGATTCCGG
TRV2-NbATG8f-Xho1-R	CCGCTCGAGGTTAAAGTCCCCAAATGTGTTTTC
NbATG8f-qPCR-F	CTTGAGAAGAGGCGTGCTGAAGC
NbATG8f-qPCR-R	ATCTGTTGGTGGTAGGACATTATCAAC
TRV2-NbATG2-BamH-F	CGGGATCCCCAATCGTCGAAGGATAATCTTAGA
TRV2-NbATG2-Xho1-R	CCGCTCGAGTCCTGTTGATCGCTGTGCATTATTCTG
TRV2-NbATG5-BamH-F	CGGGATCCCCTGGAAATATTTTAACACCTTGTG

TRV2-NbATG5-Xho1-R	CCGCTCGAGGTATTCTACCTGTTTTAGCTGGTCC
TRV2-NbATG7-BamH-F	CGGGATCCCAAGGTTGGCTGTATCAGCTGCTG
TRV2-NbATG7-Xho1-R	CCGCTCGAGCAAGACCTTCTGCTTCCACTGCTGG
TRV2-NbPI3K-BamH-F	CGGGATCCTGGCAACTGGACACGATGAGG
TRV2-NbPI3K-Xho1-R	CCGCTCGAGGGCGGTGGAAAGGGCTTAGG
TRV2-NbVPS15-BamH-F	CGGGATCCGCTGAAGGTTCTTATTTTAATACTC
TRV2-NbVPS15-Xho1-R	CCGCTCGAGTCGGGTACTAAGACGATCGTGCAG
LBb1.3-F	ATTTTGCCGATTTCGGAAC
m-Atg6-F	TGTCACCACTCCCTTACCATC
m-Atg6-R	TTGGCCAATAAGGAAACAGTG
mAtg8a44c-LP	TTTTCTGGGTTTGTTTCTCCC
mAtg8a44c-RP	TCATTAGCCCCAAAATCAATG
PePMV-Met-gw-F	GGGGACAAGTTTGTACAAAAAAGCAGGCTTCATGTCTCGTGTTAGAAACACTTTAG
PePMV-Met-gw-R	GGGGACCACTTTGTACAAGAAAGCTGGGTCTGTGGATATAAGCTGTCTAACTTTTG
PePMV-Hel-gw-F	GGGGACAAGTTTGTACAAAAAAGCAGGCTTCATGGTTGTCATACATGGTTGTGG
PePMV-Hel-gw-R	GGGGACCACTTTGTACAAGAAAGCTGGGTCATTGATAAAATGGATAGAATCCAC
PePMV-RdRp2-gw-F	GGGGACAAGTTTGTACAAAAAAGCAGGCTTCATGAACGGCAACAACTTAGAGAAA
PePMV-RdRp2-gw-R	GGGGACCACTTTGTACAAGAAAGCTGGGTCATGTGTTGCTTTTGGGGGGCTC
PePMV-RdRp2-mGDD-F	CAGTGAAACAAGTTTACGCTATGGCTCTTGACGGAGTGGTCATG
PePMV-RdRp2-mGDD-R	CATGACCACTCCGTCAAGAGCCATAGCGTAAACTTGTTTCACTG
PePMV-qPCR-F	GCTTATAGCTCAGATGTTAAGAATAAC
PePMV-qPCR-R	GGACAACAACTGTACAGCAGTCATTATTG
CGMMV-Met-gw-F	GGGGACAAGTTTGTACAAAAAAGCAGGCTTCATGTTTTCTCGTGTGGTCAGCACA
CGMMV-Met-gw-R	GGGGACCACTTTGTACAAGAAAGCTGGGTCCGGTACGTTCCACTCAGATTTC
CGMMV-Hel-gw-F	GGGGACAAGTTTGTACAAAAAAGCAGGCTTCATGACATTAGTTGACGGAGTGCCGG
CGMMV-Hel-gw-R	GGGGACCACTTTGTACAAGAAAGCTGGGTCAGTATAATACACCATTGCCTT
CGMMV-RdRp2-gw-F	GGGGACAAGTTTGTACAAAAAAGCAGGCTTCATGTTGAGGGACAACGAATTTAAC
CGMMV-RdRp2-gw-R	GGGGACCACTTTGTACAAGAAAGCTGGGTCAAGACTACGGAAAAGGCGCTTATC
CGMMV-RdRp2-mGDD-F	GTTTTAAAGCTAGTTTTTGTTCGCTGATCTACCTTCCTAAG
CGMMV-RdRp2-mGDD-R	CTTAGGAAGGTAGATCAGCGAACAAAAACTAGCTTTAAAAC
CGMMV-qPCR-F	AGGCTAACCTTGAGTAATTTAGATCCGG
CGMMV-qPCR-R	GTTATCTACTATAGAAATCGACATGTTAG
q-AtATG8a-F	ACCAGCTGATCTAACAGTGGGACA
q-AtATG8a-R	CCAAAAGTGTTCTCTCCACTGTAAG
AtATG8a-gw-F	GGGGACAAGTTTGTACAAAAAAGCAGGCTTCATGATCTTTGCTTGCTTGAAATTC
AtATG8a -gw-R	GGGGACCACTTTGTACAAGAAAGCTGGGTCAGCAACGGTAAGAGATCCA
SMV-NIb-gw-F	GGGGACAAGTTTGTACAAAAAAGCAGGCTTCATGGGGAGAAAGGAAAGATGGGTTTTG
SMV-NIb -gw-R	GGGGACCACTTTGTACAAGAAAGCTGGGTCTTGTAAGGACACTGATTCACAAC
SMV-NIb-mGDD-F	GTTTTTCGCCAATATCATTCTTGCAGT
SMV-NIb-mGDD-R	ACTGCAAGAATGATATTGGCGAAAAAC
TEV-NIb-gw-F	GGGGACAAGTTTGTACAAAAAAGCAGGCTTCATGGGGGAGAAGAGGAAATGGGTCGTG
TEV-NIb-gw-R	GGGGACCACTTTGTACAAGAAAGCTGGGTCCTGAAAATAAAGATTCTCAGTCGTTGG
TEV-NIb-mGDD-F	TGTGTATTACGTCAATCTATTGATTGCC
TEV-NIb-mGDD-R	GGCAATCAATAGATTGACGTAATACACA
